# DNA sensing via the cGAS/STING pathway activates the immunoproteasome and adaptive T‐cell immunity

**DOI:** 10.15252/embj.2022110597

**Published:** 2023-03-13

**Authors:** Xinyuan Wang, Huabin Zhang, Yuqin Wang, Laylan Bramasole, Kai Guo, Fatima Mourtada, Thomas Meul, Qianjiang Hu, Valeria Viteri, Ilona Kammerl, Melanie Konigshoff, Mareike Lehmann, Thomas Magg, Fabian Hauck, Isis E Fernandez, Silke Meiners

**Affiliations:** ^1^ Comprehensive Pneumology Center (CPC), Member of the German Center for Lung Research (DZL) University Hospital, Ludwig‐Maximilians University, Helmholtz Zentrum München Munich Germany; ^2^ State Key Laboratory of Respiratory Disease, Guangzhou Institute of Respiratory Health, The First Affiliated Hospital of Guangzhou Medical University Guangzhou Medical University Guangzhou China; ^3^ Neurosurgical Research, Department of Neurosurgery, University Hospital and Walter‐Brendel‐Centre of Experimental Medicine, Faculty of Medicine Ludwig‐Maximilians‐University Munich Germany; ^4^ The Second Affiliated Hospital of Guangzhou Medical University Guangzhou China; ^5^ Research Center Borstel/Leibniz Lung Center Borstel Germany; ^6^ Airway Research Center North (ARCN), Member of the German Center for Lung Research (DZL) Borstel Germany; ^7^ Institute of Experimental Medicine Christian‐Albrechts‐University Kiel Kiel Germany; ^8^ Research Unit Lung Repair and Regeneration, Helmholtz Zentrum München, German Research Center for Environmental Health, Member of the German Center of Lung Research (DZL) University Hospital Grosshadern, Ludwig‐Maximilians‐University Munich Germany; ^9^ Division of Pulmonary, Allergy and Critical Care Medicine, School of Medicine University of Pittsburgh School of Medicine Pittsburgh PA USA; ^10^ Division of Pediatric Immunology and Rheumatology, Department of Pediatrics, Dr. von Hauner Children's Hospital University Hospital, Ludwig‐Maximilians‐Universität München Munich Germany; ^11^ Department of Medicine V University Hospital, LMU Munich Munich Germany

**Keywords:** CD8^+^ T cells, cGAS/STING, fibrosis, immunoproteasome, mitochondria, Immunology

## Abstract

The immunoproteasome is a specialized type of proteasome involved in MHC class I antigen presentation, antiviral adaptive immunity, autoimmunity, and is also part of a broader response to stress. Whether the immunoproteasome is regulated by DNA stress, however, is not known. We here demonstrate that mitochondrial DNA stress upregulates the immunoproteasome and MHC class I antigen presentation pathway via cGAS/STING/type I interferon signaling resulting in cell autonomous activation of CD8^+^ T cells. The cGAS/STING‐induced adaptive immune response is also observed in response to genomic DNA and is conserved in epithelial and mesenchymal cells of mice and men. In patients with idiopathic pulmonary fibrosis, chronic activation of the cGAS/STING‐induced adaptive immune response in aberrant lung epithelial cells concurs with CD8^+^ T‐cell activation in diseased lungs. Genetic depletion of the immunoproteasome and specific immunoproteasome inhibitors counteract DNA stress induced cytotoxic CD8^+^ T‐cell activation. Our data thus unravel cytoplasmic DNA sensing via the cGAS/STING pathway as an activator of the immunoproteasome and CD8^+^ T cells. This represents a novel potential pathomechanism for pulmonary fibrosis that opens new therapeutic perspectives.

## Graphical abstract

The DNA‐sensing cGAS/STING pathway regulates immunoproteasome function and MHC class I antigen presentation to promote T‐cell activation that may underlie pathology of idiopathic pulmonary fibrosis.

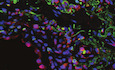

## Introduction

Immunoproteasomes are specialized proteasomes that contain three inducible catalytic subunits, namely LMP2, MECL‐1, and LMP7. They are constitutively present in immune cells (Basler *et al*, 2013). In nonimmune cells, these catalytic immunoproteasome subunits are transcriptionally induced upon inflammatory signaling (e.g., interferon (IFN) α, β, or γ, LPS, TNFα) or virus infection and rapidly assemble into immunoproteasomes (Sijts & Kloetzel, 2011; Murata *et al*, 2018, 201). Immunoproteasomes hydrolyze intracellular proteins with different cleavage specificities and/or proteolytic rates compared with the standard proteasome that is the constitutively expressed type of proteasome in all nonimmune cells (Huber *et al*, 2012; Basler *et al*, 2013; Harshbarger *et al*, 2015; Winter *et al*, 2017). Immunoproteasome function has been shown to be crucial for antiviral immune responses. Their induction in virus‐infected cells by IFNγ allows degradation of viral proteins and generation of antigenic peptides for presentation on MHC class I molecules and subsequent activation of cytotoxic CD8^+^ T cells (Sijts & Kloetzel, 2011). Immunoproteasome activity is thus crucial for effectively combating virus infections as demonstrated in genetic mouse models (Fehling *et al*, 1994; Van Kaert *et al*, 1994; Sijts & Kloetzel, 2011; Kincaid *et al*, 2012). Moreover, immunoproteasome function has been implicated in multiple autoimmune diseases possibly due to its key role in T‐cell differentiation (Muchamuel *et al*, 2009; Basler *et al*, 2015, 2017). Specific inhibition of the immunoproteasome effectively counteracts experimental autoimmune diseases (Basler *et al*, 2015; Kammerl & Meiners, 2016; Morozov & Karpov, 2018). Immunoproteasome activation also emerges as part of a broader response to stress such as protein and oxidative stress and the unfolded protein response (Seifert *et al*, 2010; Raynes *et al*, 2016; Yun *et al*, 2016; Studencka‐Turski *et al*, 2019). Whether the immunoproteasome is also regulated by DNA stress, however, has not been analyzed.

We here demonstrate that mtDNA and cytoplasmic DNA in general induces the immunoproteasome in parenchymal cells via the cGAS/STING/type I interferon pathway resulting in autoreactive CD8^+^ T‐cell activation. DNA stress‐induced cGAS/STING signaling thus not only triggers innate but also triggers adaptive immune responses. Moreover, we provide evidence for an activation of this pathway, which we term cGAS/STING‐induced adaptive immune response, in aberrant epithelial cells from patients with idiopathic pulmonary fibrosis (IPF). In addition, we demonstrate the activation of tissue resident and circulating CD8^+^ T cells in cohorts of IPF patients. Our data thus link induction of the immunoproteasome by DNA stress to cell‐intrinsic and chronic activation of autoreactive CD8^+^ T cells via the cGAS/STING pathway suggesting a novel pathomechanism for pulmonary fibrosis.

## Results

We investigated the activation of the immunoproteasome by mitochondrial dysfunction using mouse embryonic fibroblasts (MEFs) that express a proofreading‐deficient mitochondrial DNA polymerase gamma (*PolgA* ^mut/mut^). This defect results in the accumulation of random point mutations in their mtDNA that causes dysfunction of the respiratory chain in the absence of oxidative stress (Trifunovic *et al*, 2004; Meul *et al*, 2020). We performed our experiments with either three lines of *PolgA* ^+/+^ or *PolgA* ^mut/mut^ cells to reduce clonal differences between the cells (Edgar *et al*, 2009; Meul *et al*, 2020). *PolgA* ^mut/mut^ cells are fully viable, maintain an intact mitochondrial structure and network, and show no signs of cellular stress (Meul *et al*, 2020).

### Activation of the immunoproteasome in respiration defective mtDNA mutant cells

**Figure 1:**
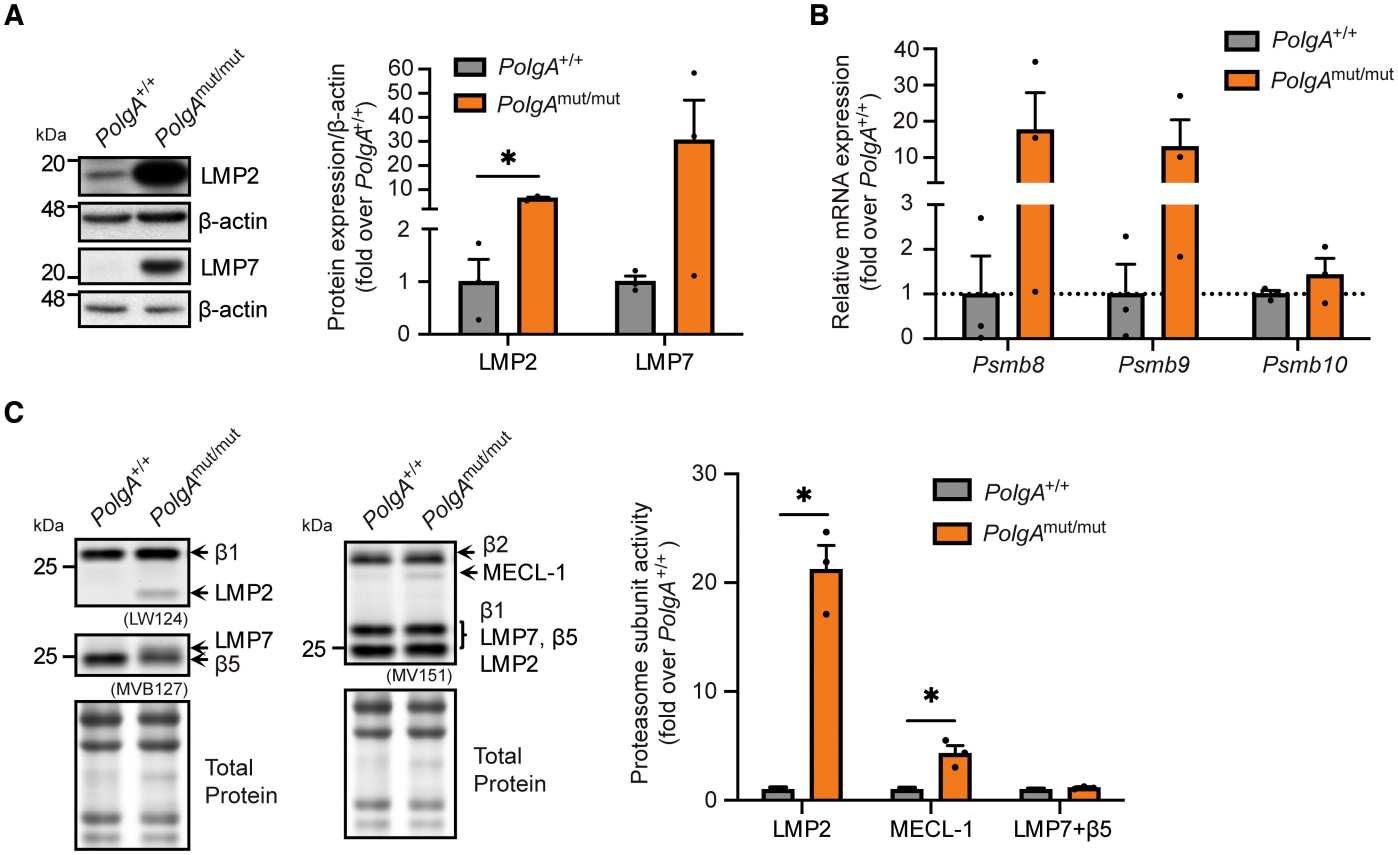
Activation of mtDNA stress in *PolgA* ^mut/mut^ cells Western blot analysis of the immunoproteasome subunits LMP2, LMP7 and β‐actin (loading control) in three *PolgA* ^*+/+*^ and three *PolgA* ^mut/mut^ cell lines.RT‐qPCR analysis of immunoproteasome subunits *Psmb8*, *Psmb9*, *Psmb10* in three different *PolgA* ^+/+^ and three *PolgA* ^mut/mut^ cell lines.Activity assay for the catalytic subunits of the proteasome using the pan activity‐based probe (ABP) MV151, the β1/LMP2 specific LW124, and the β5/LMP7 specific MVB127 ABPs in three *PolgA* ^*+/+*^ and three *PolgA* ^mut/mut^ cell lines. Resolution of β5/LMP7 in the SDS gel is hampered as both subunits are of similar molecular weight and thus do not separate in our gels. Total protein staining was used as a loading control. Data information: Data are shown as the mean ± SEM of three biological replicates and were analyzed with two‐tailed unpaired Student's *t*‐test. Asterisks indicate significance as *P < 0.05. Source data are available online for this figure.

In *PolgA* ^mut/mut^ cells, we noticed an unexpected activation of the immunoproteasome. Two catalytic subunits of the immunoproteasome, that is, LMP2 and LMP7 (gene names *Psmb9* and *Psmb8*, respectively), were strongly upregulated both on the protein (Fig 1A) and RNA level (Fig 1B). To demonstrate incorporation of the immunoproteasome subunits into active proteasome complexes, we made use of a set of three activity‐based probes, which specifically label the active catalytic subunits of the proteasome. As these probes are attached to a fluorophore, covalent binding of the activity‐based probe (ABP) to the respective active site can be detected by separating the labeled proteolytic subunits of the proteasome according to their size in denaturing gels (de Bruin *et al*, 2016; Kammerl *et al*, 2021). Importantly, transcriptional induction of these subunits resulted in the assembly of active immunoproteasome complexes containing all three immunoproteasome subunits, namely LMP2, MECL1, and LMP7 in *PolgA* ^mut/mut^ cells while the immunoproteasome was virtually absent in *PolgA* ^+/+^ cells (Fig 1C).

Our data thus demonstrate activation of the immunoproteasome in cells with mitochondrial dysfunction.

### Activation of mtDNA stress in *PolgA* ^mut/mut^ cells

**Figure 2:**
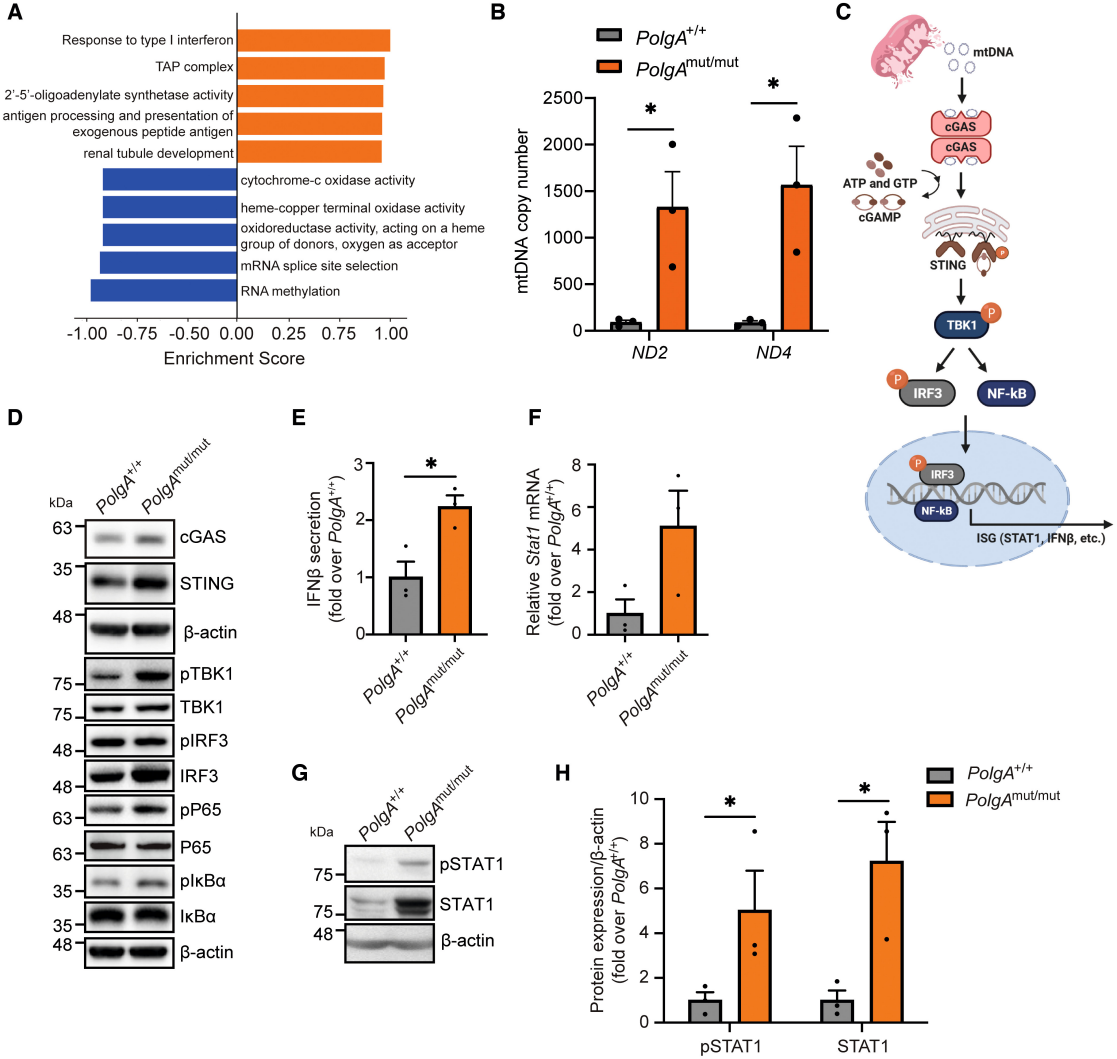
Activation of the cGAS/STING pathway in *PolgA* ^mut/mut^ cells Gene annotation enrichment analysis showing the top five upregulated (orange) and downregulated (blue) GO terms in a data set obtained from proteomic analysis of total protein extracts of *PolgA* ^mut/mut^ (*n* = 4) and *PolgA* ^+/+^ (*n* = 3) cells.Absolute quantification of the cytoplasmic mtDNA genes *Nd2* and *Nd4* in *PolgA* ^*+/+*^ and *PolgA* ^mut/mut^ cell lines.Scheme of cGAS/STING pathway activation upon mtDNA sensing (Created with BioRender.com).Representative Western blots for the analysis of the cGAS/STING signaling pathway in *PolgA* ^*+/+*^ and *PolgA* ^mut/mut^ cells with β‐actin serving as loading control.Secreted IFNβ in supernatants from three distinct *PolgA* ^*+/+*^ and *PolgA* ^mut/mut^ cell lines. The 15.81 pg/ml detected by ELISA in *PolgA* ^mut/mut^ cell supernatants corresponds to 18.97 IU/ml recombinant mouse (rm) IFN‐β according to the R&D Systems cytokine conversion table (https://www.rndsystems.com/cn/resources/technical‐information/unit‐conversion‐table).RT‐qPCR analysis of *Stat1* in three different *PolgA* ^*+/+*^ and three *PolgA* ^mut/mut^ cell lines.Western blot analysis of pospho (Tyr701)‐STAT1 and STAT1 with β‐actin as loading control in *PolgA* ^*+/+*^ and *PolgA* ^mut/mut^ cells.Densitometric analysis of pSTAT1 and STAT1 expression normalized to β‐actin and set to 1 in three *PolgA* ^*+/+*^ or three *PolgA* ^mut/mut^ cell lines. Data information: Data are shown as the mean ± SEM of three biological replicates and were analyzed with two‐tailed unpaired Student's *t*‐test. Asterisks indicate significance as *P < 0.05. Source data are available online for this figure.

To investigate the potential mechanism of immunoproteasome induction in cells with respiratory dysfunction, we performed proteomic analysis of total protein extracts of *PolgA* ^mut/mut^ compared with *PolgA* ^+/+^ cells. Unbiased 1D‐enrichment analysis allowed us to identify the most prominently regulated pathways. As shown in Fig 2A and Table EV1, we noted prominent downregulation of proteins involved in mitochondrial metabolism (e.g., oxidase and oxireductase activities) as well as in RNA splicing and methylation. This probably reflects the metabolic reprogramming of *PolgA* ^mut/mut^ cells that we recently characterized in detail (Meul *et al*, 2020). By contrast, three of the five most highly upregulated pathways were related to type I interferon signaling, the transporter associated with antigen processing (TAP) complex, and antigen processing and presentation (Fig 2A).

**Figure EV1:**
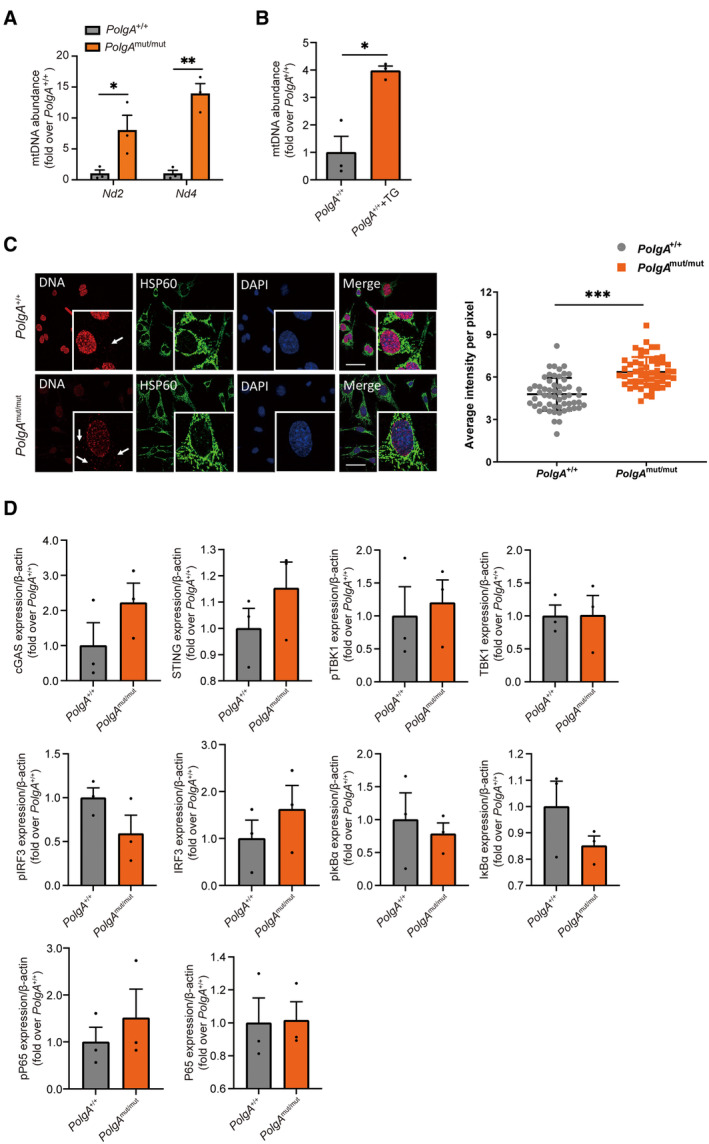
Mitochondrial DNA stress in *PolgA* ^mut/mut^ cells Abundance of cytoplasmic mtDNA genes *Nd2* and *Nd4* in three *PolgA* ^*+/+*^ and three *PolgA* ^mut/mut^ cell lines as biological replicates. Values are given as fold over *PolgA* ^+/+^ control with the mean of normalized *PolgA* ^+/+^ signal set to 1. Single data points are provided to denote the individual cell lines.Cytoplasmic mtDNA genes *Nd2* in *PolgA* ^+/+^ cells treated with 10 μM thapsigargin for 6 h serving as a positive control for the detection of cytosolic mtDNA. Three independent experiments were performed.Detection of cytoplasmic DNA in *PolgA* ^+/+^ and *PolgA* ^mut/mut^ cells by using confocal microscope. Cells were stained with DAPI (blue), anti‐HSP60 (green) and anti‐DNA (red). Arrows indicate red dsDNA signals outside the nucleus. Scale bar on the figures represents 50 μm. Quantification of cytoplasmic DNA was from independent fields (*n* ≥ 6) with 62 *PolgA* ^+/+^ and 55 *PolgA* ^mut/mut^ cells counted.Densitometric analysis of cGAS, STING, pTBK1, TBK1, pIRF3, IRF3, pIκBα, IκBα, pP65, and P65 expression normalized to β‐actin loading control in *PolgA* ^+/+^ and *PolgA* ^mut/mut^ cells from three biological replicates as exemplarily shown in Western blot in Fig 2D. Data information: Data are shown as mean ± SEM and were analyzed with two‐tailed unpaired Student's *t*‐test. Asterisks indicate significance as *P < 0.05, **P < 0.01, ***P < 0.001.

As these pathways are all closely linked to immunoproteasome activation (Sijts & Kloetzel, 2011; Lee & Ashkar, 2018), we hypothesized that *PolgA* ^mut/mut^ cells experience mtDNA stress with mtDNA leaking out of the mitochondria into the cytosol to activate type I interferon signaling (West & Shadel, 2017). PCR analysis for the two mitochondrially encoded genes *Nd2* and *Nd4* in cytoplasmic DNA fractions revealed the presence of cytosolic mtDNA specifically in *PolgA* ^mut/mut^ but not in wild‐type cells as shown by absolute and relative mtDNA quantification (Figs 2B and EV1A). Thapsigargin‐induced mtDNA release into the cytosol was used a positive control in this experiment (Fig EV1B). We also performed staining for double‐stranded (ds) DNA to detect the presence of cytosolic DNA in *PolgA* ^mut/mut^ cells. Co‐staining with anti‐DNA for total DNA, DAPI for nuclear and hsp60 for visualization of the mitochondrial network allowed us to detect weak but distinct dsDNA signals in the cytosol of the cells that were significantly more abundant in *PolgA* ^mut/mut^ cells (Fig EV1C). To investigate whether the presence of mtDNA is essential for immunoproteasome induction, we depleted mtDNA from *PolgA* ^mut/mut^ cells. This approach, however, failed as mtDNA depletion strongly impaired cell growth of *PolgA* ^mut/mut^ but not *PolgA* ^+/+^ cells indicating that the presence of mitochondrial DNA is crucial for cell growth in *PolgA* ^mut/mut^ cells.

**Figure EV2:**
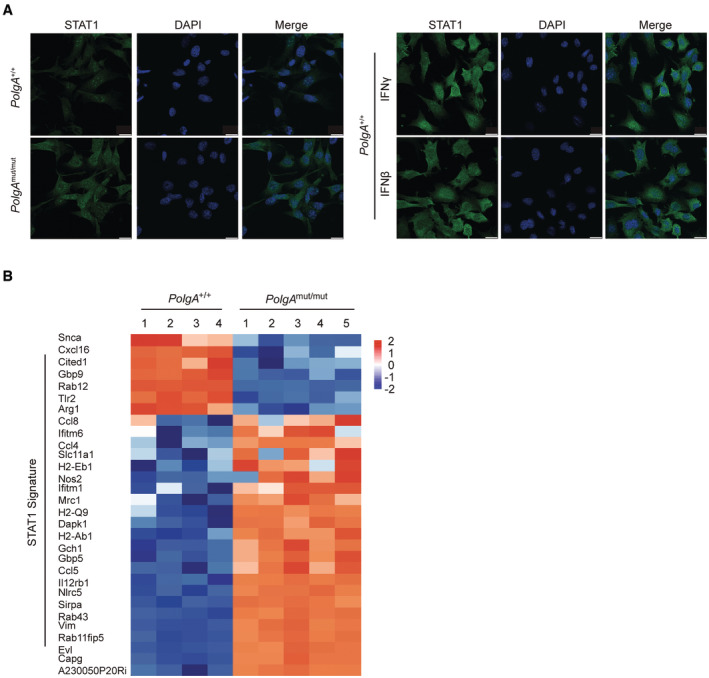
Activation of STAT1 in *PolgA* ^mut/mut^ cells Immunofluorescence analysis of STAT1 in *PolgA* ^+/+^ and *PolgA* ^mut/mut^ cells, as well as in *PolgA* ^+/+^ cells treated with 100 IU/ml IFNβ or 75 IU/ml IFNγ for 24 h, scale bar: 20 μm.Heatmap of mRNA expression values of significantly regulated STAT1‐stimulated genes obtained from RNA seq analysis of *PolgA* ^+/+^ (four independent replicates of the same cell line) and *PolgA* ^mut/mut^ (five independent replicates of the same cell line) cells.

Intracellular mtDNA is sensed by the cGAS/STING pathway that thereby activates innate immune responses via type I interferon signaling (Chen *et al*, 2016; West & Shadel, 2017; Zhang *et al*, 2020) (Fig 2C). We thus investigated the activation of the cGAS/STING pathway in *PolgA* ^mut/mut^ cells by Western blotting for its key components (Figs 2D and EV1D). While we were unable to detect significant changes in the expression of cGAS and STING in the three distinct *PolgA* ^mut/mut^ cell lines compared with our three wild‐type lines due to high clonal variation, levels were slightly higher in *PolgA* ^mut/mut^ cells. The same applied to the levels of phosphorylated—and thus activated—NFκB p65. Expression levels of IκBα were slightly lower in *PolgA* ^mut/mut^ cells (Figs 2D and EV1D). Secretion of IFNβ was upregulated by more than twofold in cells with mtDNA mutations (Fig 2E). Moreover, the transcription factor STAT1 was strongly induced on the transcriptional level (Fig 2F) resulting in higher protein levels of total and phosphorylated STAT1 (Figs 2G and H, and EV2A). Concomitantly, multiple STAT1‐stimulated genes were upregulated in *PolgA* ^mut/mut^ cells (Fig EV2B) as revealed by RNA sequencing. These data suggest that *PolgA* ^mut/mut^ cells experience mtDNA stress with activation of the cGAS/STING signaling pathway, release of IFNβ into the supernatant and STAT1 activation.

### Activation of the immunoproteasome by mtDNA stress via cGAS/STING signaling

**Figure 3:**
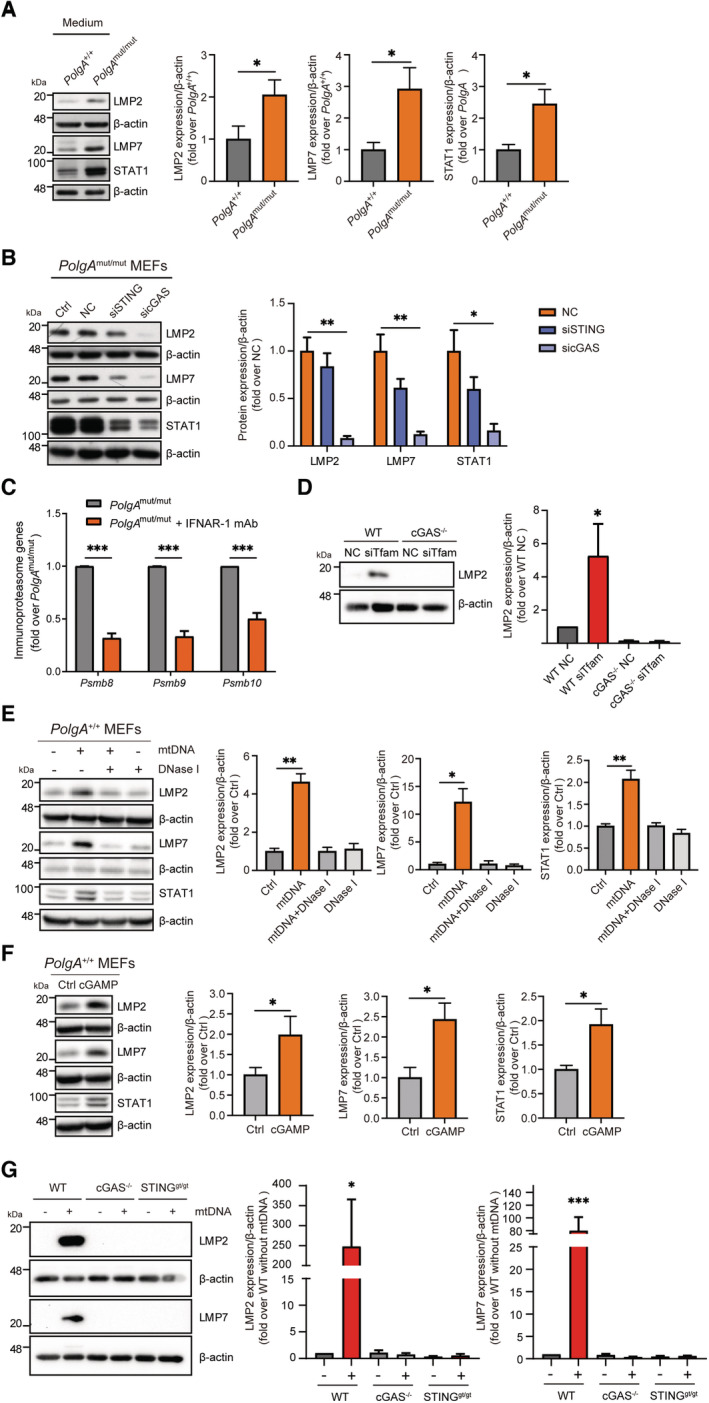
Activation of the immunoproteasome by mtDNA via the cGAS/STING pathway Western blot analysis of LMP2, LMP7, STAT1 and β‐actin (loading control) in *PolgA* ^*+/+*^ cells after incubation with either *PolgA* ^*+/+*^ or *PolgA* ^mut/mut^ cell‐conditioned medium for 48 h. Densitometric analysis of LMP2, LMP7 and STAT1 expression normalized to β‐actin loading control set to 1 in *PolgA* ^*+/+*^ incubated with either *PolgA* ^*+/+*^ or *PolgA* ^mut/mut^ cell‐conditioned medium from three different cell lines.Western blot analysis of LMP2, LMP7, STAT1, and β‐actin (loading control) upon siRNA‐mediated silencing of cGAS or STING in *PolgA* ^mut/mut^ cells compared to untransfected (Ctrl) and non‐sense scrambled transfection control (NC) in three independent experiments.RNA expression of the immunoproteasomal subunits PSMB8‐10 normalized to Rpl19 in *PolgA* ^mut/mut^ cells treated with 0.5 μg/ml IFNAR‐1 antibody for 48 h.Western blot analysis of LMP2 and β‐actin (loading control) in wildtype (WT) and cGAS KO MEFs that had been partially depleted of TFAM by siRNA treatment for 72 h.Western blot analysis of LMP2, LMP7, STAT1, and β‐actin (loading control) in *PolgA* ^*+/+*^ cells transfected with mouse wildtype mtDNA, mtDNA pretreated with DNase I or only DNase I for 48 h. Densitometric analysis of LMP2, LMP7 and STAT1 expression normalized to β‐actin loading control in *PolgA* ^*+/+*^ cells. Values are given as fold over *PolgA* ^*+/+*^ control with the mean of normalized *PolgA* ^*+/+*^ signal normalized to 1.Western blot analysis of LMP2, LMP7, STAT1 and β‐actin (loading control) in *PolgA* ^*+/+*^ cells transfected with cGAMP for 24 h. Densitometric analysis of LMP2, LMP7 and STAT1 expression normalized to β‐actin loading control (Ctrl) set to 1.Protein expression of LMP2, LMP7 and β‐actin (loading control) in wildtype (WT), cGAS KO or STING^gt/gt^ loss‐of‐function MEFs that had been transfected with 200 ng/ml mtDNA for 48 h. Data information: Data are shown as the mean ± SEM from three independent experiments and were analyzed with two‐tailed unpaired Student's *t*‐test. Asterisks indicate significance as *P < 0.05, **P < 0.01, ***P < 0.001. Source data are available online for this figure.

**Figure EV3:**
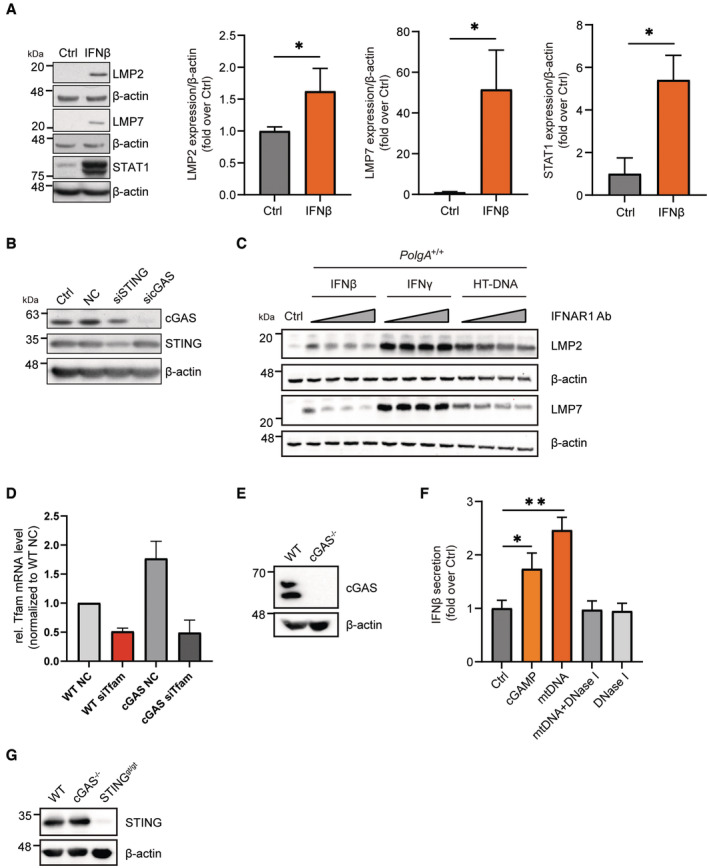
cGAS/STING‐dependent induction of the immunoproteasome upon mitochondrial DNA stress Representative Western blot and densitometric analysis for LMP2, LMP7, STAT1 and β‐actin (loading control) in extracts of *PolgA* ^+/+^ cells treated with 100 IU/ml IFNβ for 48 h in three independent biological experiments.Representative Western blot for cGAS and STING in *PolgA* ^mut/mut^ cells upon siRNA transfection of non‐sense scrambled control (NC), cGAS or STING‐specific siRNAs. Untransfected cells were used as controls (Ctrl). β‐actin served as a loading control.Western blot for LMP2 and LMP7 in *PolgA* ^+/+^ cells pretreated with 0.04 μg/ml, 0.1 μg/ml, 0.2 μg/ml or 0.5 μg/ml IFNAR1 Ab for 24 h and treated with IFNβ, IFNγ or transfected with HT‐DNA for 48 h.RT‐qPCR analysis of TFAM in wild‐type (WT) and cGAS KO MEFs upon silencing of TFAM with siRNA from three independent biological experiments.Representative Western blot for cGAS in wild‐type (WT) and cGAS KO MEFs, β‐actin served as a loading control.ELISA of secreted IFNβ in supernatants from *PolgA* ^*+/+*^ cells transfected with cGAMP, mtDNA, DNase I pretreated mtDNA and DNase I (*n* = 3 independent biological experiments).Representative Western blot for STING in wild‐type (WT), cGAS KO MEFs and STING^gt/gt^ loss‐of‐function MEFs, β‐actin served as a loading control. Data information: All data are shown as mean ± SEM and were analyzed with two‐tailed unpaired Student's *t*‐test. Asterisks indicate significance as *P < 0.05, **P < 0.01.

In a next step, we tested whether the cGAS/STING signaling pathway controls immunoproteasome induction. Conditioned medium from *PolgA* ^mut/mut^ cells upregulated both, the immunoproteasome and STAT1, as shown by Western blot analysis (Fig 3A). This induction was similar to the upregulation of the immunoproteasome subunits LMP2, LMP7, and STAT1 when *PolgA* ^*+/+*^ cells were stimulated with IFNβ (Fig EV3A). To test involvement of the cGAS/STING pathway in immunoproteasome induction in *PolgA* ^mut/mut^ cells, we silenced either cGAS or STING in these cells (Figs 3B and EV3B) and analyzed immunoproteasome and STAT1 expression. LMP2, LMP7, and STAT1 protein levels were markedly downregulated upon cGAS or STING silencing (Fig 3B). Using an antagonizing antibody against the mouse interferon alpha/beta receptor subunit 1 (IFNAR1) to block IFNβ signaling (Fig EV3C), we were able to demonstrate diminished transcriptional induction of the immunoproteasome in *PolgA* ^mut/mut^ cells upon inhibition of IFNβ signaling (Fig 3C).

We also validated the activation of the immunoproteasome upon partial silencing of the mitochondrial master regulator TFAM in *PolgA* ^+/+^ wild‐type cells. The immunoproteasome subunit LMP2 was significantly induced upon partial TFAM depletion compared with controls. Induction was abrogated in cGAS knockout MEFs (Figs 3D, and EV3D and E). Our data thus demonstrate the activation of the immunoproteasome in cells with mitochondrial dysfunction.

To validate whether cytoplasmic mtDNA sensing via the cGAS/STING pathway is sufficient to activate the immunoproteasome, we transfected mtDNA into *PolgA* ^+/+^ cells. The expression of the immunoproteasome and STAT1 was upregulated (Fig 3E) and secretion of IFNβ was increased (Fig EV3F). This effect was completely abrogated by DNase I pretreatment of transfected mtDNA (Figs 3E and EV3F). The immunoproteasome, STAT1, and IFNβ were also upregulated upon direct activation of STING by transfection of the STING agonist cGAMP (Figs 3F and EV3F). We further corroborated these data using MEFs obtained from cGAS knockout (KO) and STING‐mutant mice (Fig EV3E and G). Transfection of mtDNA into these cells failed to upregulate the immunoproteasome in both, cGAS‐depleted or STING‐mutant cells, when compared to wild‐type MEFs (Fig 3G). These results demonstrate that cGAS/STING/type I interferon signaling is key for the induction of the immunoproteasome by mtDNA stress.

### mtDNA stress directly activates autoreactive CD8^+^ T cells via the immunoproteasome/MHC I pathway

**Figure 4:**
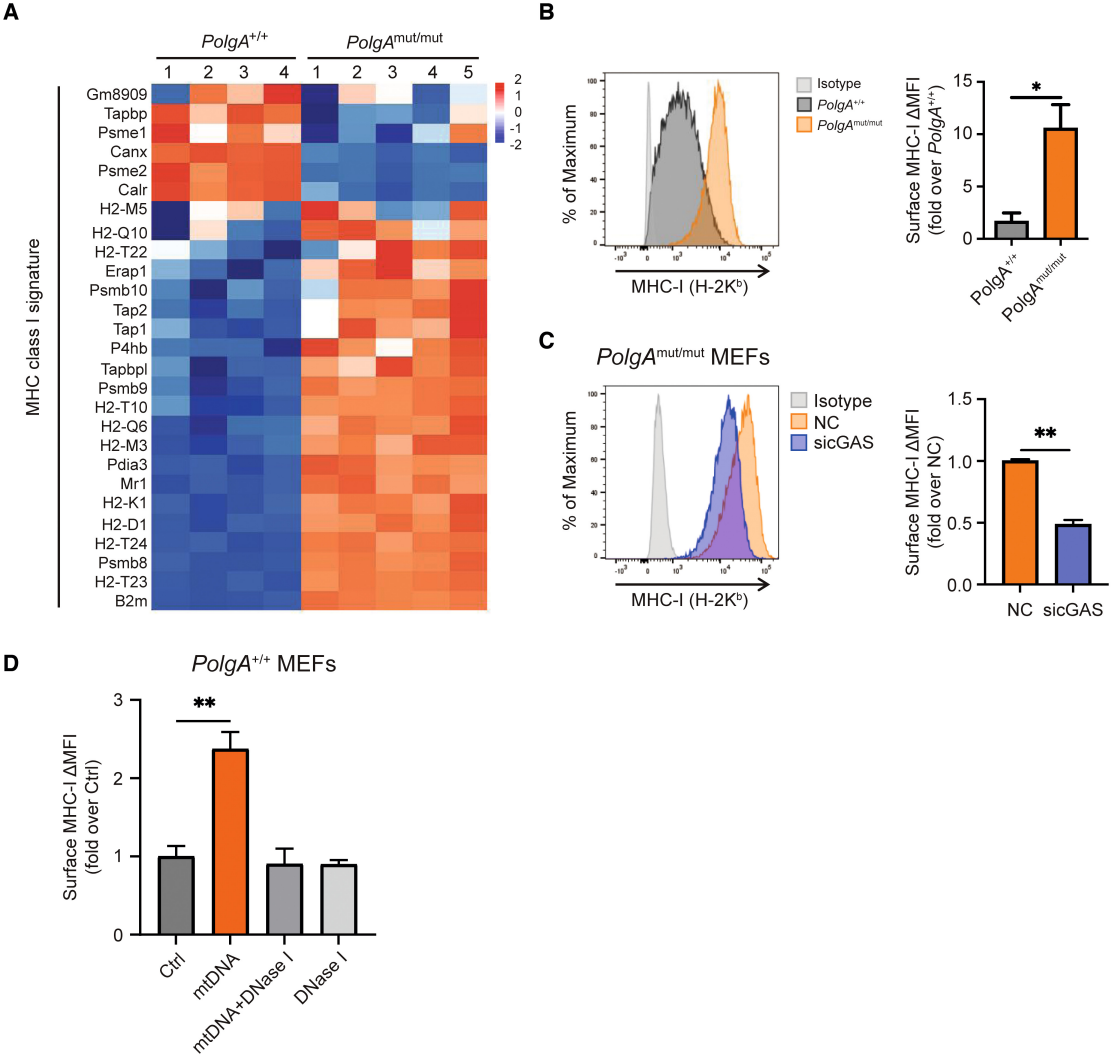
Upregulation of MHC class I antigen presentation pathway in *PolgA* ^mut/mut^ cells and by mtDNA transfection Heatmap of Z scored expression values of significantly regulated genes of the MHC class I antigen presentation pathway from *PolgA* ^*+/+*^ and *PolgA* ^mut/mut^ cells. The different ns represent biological replicates of a single *PolgA* ^*+/+*^ and *PolgA* ^mut/mut^ cell line which were all independently processed.Flow cytometric analysis of MHC‐I H‐2K^b^ surface expression in *PolgA* ^*+/+*^ and *PolgA* ^mut/mut^ cells (*n* = three independent experiments).MHC‐I H‐2K^b^ surface expression upon siRNA‐mediated knockdown of cGAS in *PolgA* ^mut/mut^ cells in three independent experiments.Flow cytometric analysis of MHC‐I H‐2K^b^ surface expression of *PolgA* ^*+/+*^ cells transfected with mtDNA, mtDNA pretreated with DNase I or DNase I alone compared to control (Ctrl) from three independent experiments. Data information: Data are shown as the mean ± SEM from three independent experiments and were analyzed with two‐tailed unpaired Student's *t*‐test. Asterisks indicate significance as *P < 0.05, **P < 0.01. Source data are available online for this figure.

**Figure EV4:**
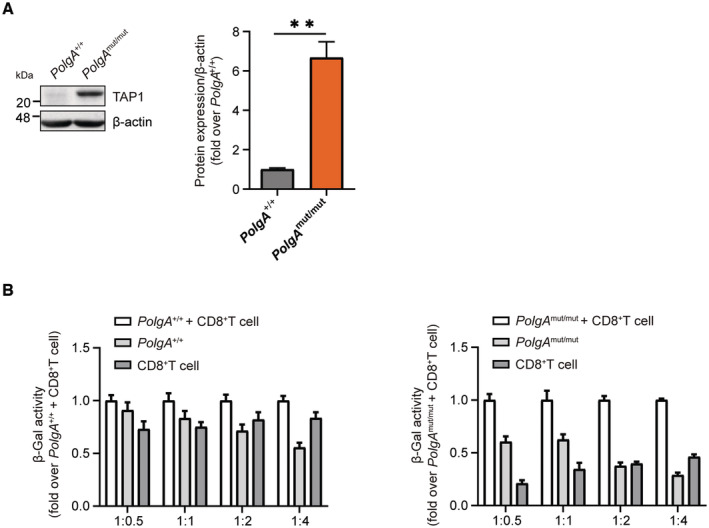
TAP1 expression and CD8^+^ T‐cell reporter activation assay Western blot analysis of TAP1 and β‐actin (loading control) and densitometric analysis from three *PolgA* ^+/+^ and three *PolgA* ^mut/mut^ cell lines. Densitometric analysis shows mean ± SEM of three biological replicates and is presented as fold over mean of *PolgA* ^+/+^ set to 1.CD8^+^ reporter T cell background in co‐culture with *PolgA* ^+/+^ or *PolgA* ^mut/mut^ cells. Data are provided as normalized values to the β‐galactosidase (β‐Gal) activity of co‐cultured MEFs and CD8^+^ T reporter cells. Data show mean ± SEM of three independent biological experiments. Data information: All data were analyzed with two‐tailed unpaired Student's *t*‐test. Asterisks indicate significance as **P < 0.01.

The activation of the immunoproteasome in *PolgA* ^mut/mut^ cells was accompanied by induction of MHC class I antigen presentation pathway as indicated by our proteomic analyses (Fig 2A) and confirmed by RNA sequencing (Fig 4A). Upregulated genes included several MHC class I molecules and peptide transporters. Higher surface expression of the mouse MHC I allele H‐2K^b^ was confirmed in *PolgA* ^mut/mut^ cells by flow cytometry (Fig 4B) and for TAP1 by Western blotting (Fig EV4A). Induction of MHC class I surface expression was dependent on cGAS/STING signaling in these cells. While silencing of cGAS diminished surface expression of H‐2K^b^ in *PolgA* ^mut/mut^ cells (Fig 4C), transfection of mtDNA into *PolgA* ^+/+^ cells upregulated the expression of MHC class I (Fig 4D). This effect was abrogated by DNase I pretreatment of transfected mtDNA (Fig 4D).

**Figure 5:**
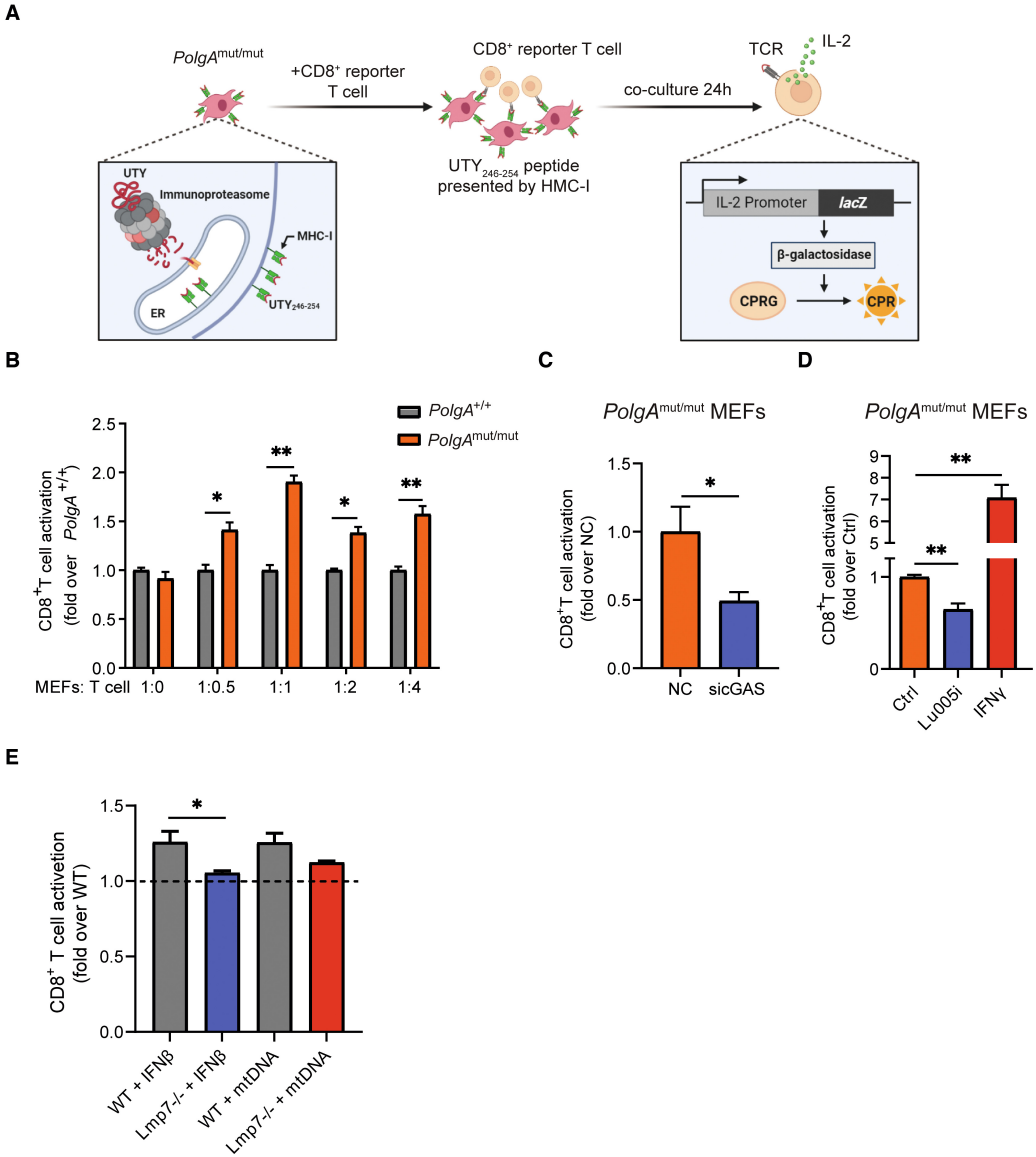
Activation of CD8^+^ T cells by *PolgA* ^mut/mut^ cells depends on the immunoproteasome Scheme of the UTY‐antigen presentation assay detecting β‐galactosidase‐mediated turnover of chlorophenol red‐β‐D‐galactopyranoside (CPRG) into CPR in CD8^+^ reporter T cells (Created with BioRender.com).UTY‐assay of CD8^+^ reporter T cells upon co‐culture with different ratios of *PolgA* ^*+/+*^ or *PolgA* ^mut/mut^ cells. Data are combined results of three independent experiments and normalized to the signal of maximum induction of *PolgA* ^*+/+*^ cells in co‐culture with CD8^+^ reporter T cells.UTY‐assay of CD8^+^ reporter T cells upon co‐culture with *PolgA* ^mut/mut^ MEFs that had been transfected either with scrambled or cGAS‐specific siRNAs. Ratio of co‐cultured cells was 1:2 (MEFs:T cells).UTY‐assay of CD8^+^ reporter T cells upon co‐culture with *PolgA* ^mut/mut^ cells treated with the immunoproteasome inhibitor Lu005i or IFNγ for 48 h. Ratio of co‐cultured cells was 1:2 (MEFs:T cells).UTY‐assay of CD8^+^ reporter T cells upon 1:2 co‐culture (splenocytes:T cells) with splenocytes isolated from either WT or LMP7 KO mice and transfected with mtDNA. Splenocytes were isolated from 4 mice each in two different preparations. Data information: Data are shown as mean ± SEM of at least three independent experiments and were analyzed with two‐tailed unpaired Student's *t*‐test. Asterisks indicate significance as *P < 0.05, **P < 0.01. Source data are available online for this figure.

We next analyzed the functional consequences of mtDNA stress‐induced activation of the immunoproteasome/MHC class I pathway. For that, we employed a CD8^+^ T‐cell activation assay that involves the immunoproteasome‐specific generation of the antigenic peptide UTY_264‐254_ from the Y chromosome‐encoded histone demethylase UTY. This antigenic peptide is bound to the MHC class I H‐2K^b^ allele and presented to a CD8^+^ UTY_264‐254_ reporter T‐cell clone (Fig 5A) (Kammerl *et al*, 2016). Successful recognition of the UTY_264‐254_ peptide on MEFs isolated from male C57BL/6 by the CD8^+^ reporter T cells activates their IL‐2 driven β‐galactosidase‐activity (Fig 5A). Of note, we observed enhanced activation of the CD8^+^ reporter T cells when co‐cultured with *PolgA* ^mut/mut^ cells compared with wild‐type controls (Figs 5B and EV4B). Importantly, silencing of cGAS in *PolgA* ^mut/mut^ cells suppressed the activation of CD8^+^ reporter T cells (Fig 5C). To corroborate the contribution of the immunoproteasome in autoreactive CD8^+^ T‐cell activation, we treated *PolgA* ^mut/mut^ cells with the specific immunoproteasome inhibitor Lu005i (de Bruin *et al*, 2014). The activation of CD8^+^ reporter T cells was reduced in *PolgA* ^mut/mut^ cells upon immunoproteasome inhibition (Fig 5D). Stimulation of the cells with IFNγ served as a positive control to activate CD8^+^ reporter T cells (Fig 5D). We analyzed the critical role of the immunoproteasome for CD8^+^ T‐cell activation by mtDNA using splenocytes obtained from male LMP7 KO and WT mice. For that, splenocytes were isolated from the respective mice, transfected with mtDNA, and then analyzed in the CD8^+^ UTY_264‐254_ reporter T‐cell assay. In contrast to MEFs, splenocytes as immune cells express immunoproteasomal genes already at baseline (Kammerl *et al*, 2016). We thus first tested whether UTY_264‐254_ presentation on the MHC class I H‐2K^b^ allele could be further activated by IFNβ treatment resulting in UTY‐specific CD8^+^ T‐cell activation. Treatment of WT splenocytes with IFNβ slightly but significantly activated the CD8^+^ reporter activity, while this was abrogated in LMP7 KO cells (Fig 5E). mtDNA transfection of WT cells similarly activated CD8^+^ T cells, while LMP7 KO cells showed lower levels of activation, which was, however, still significantly higher in the transfection control (Fig 5E).

Taken together, our results thus demonstrate that mtDNA sensing via the cGAS/STING pathway activates adaptive immune responses, that is, CD8^+^ T cells involving the immunoproteasome.

### mtDNA stress activates the immunoproteasome/MHC class I pathway in primary mouse and human lung cells

**Figure 6:**
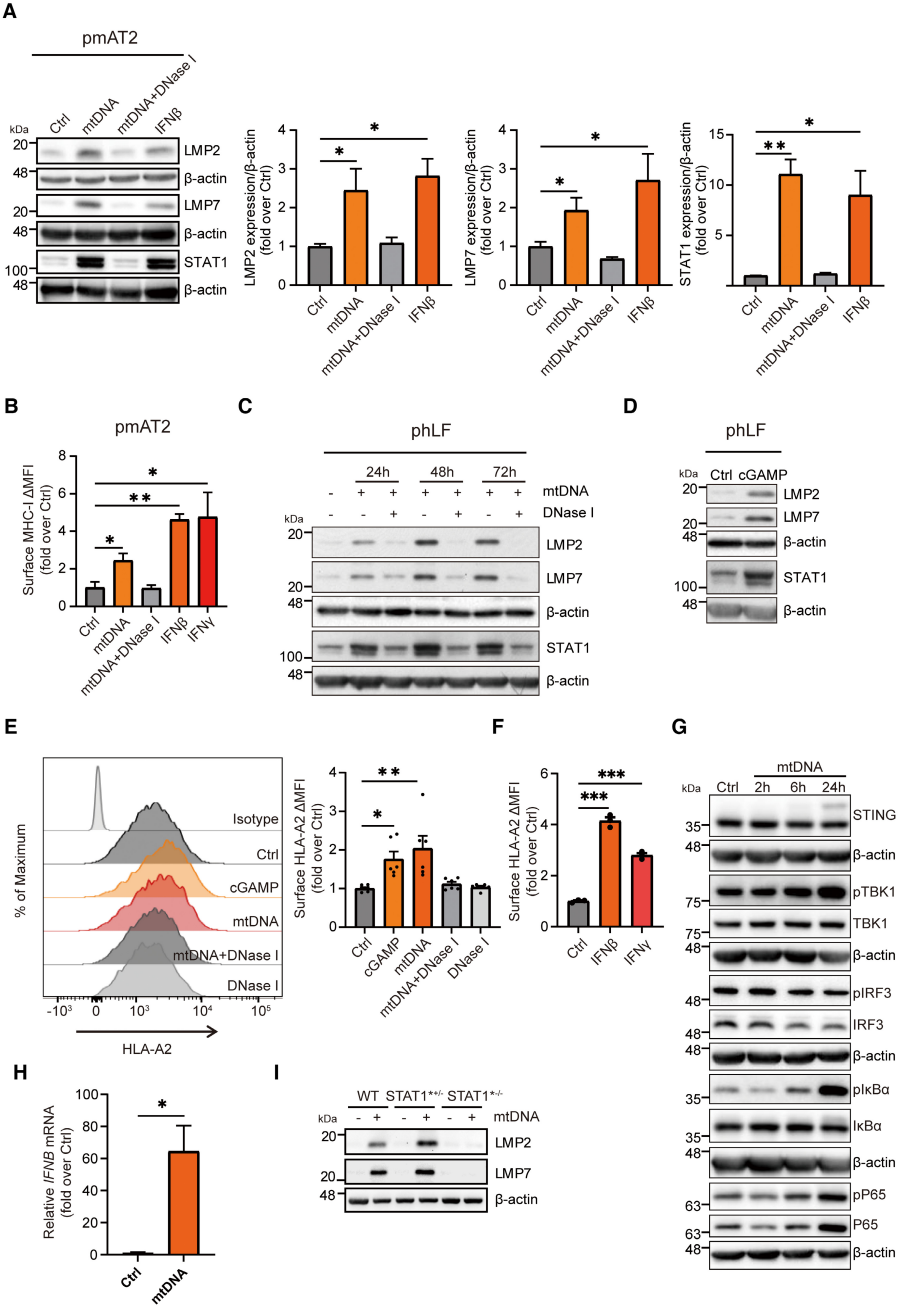
Induction of the immunoproteasome by mtDNA via the cGAS/STING pathway in mouse alveolar epithelial cells and human lung and skin fibroblasts Western blot analysis of immunoproteasome subunits LMP2, LMP7, and of STAT1 in pmAT2 cells transfected with mtDNA or mtDNA pretreated with DNase I for 48 h or treated with IFNβ for 24 h. β‐actin was used as a loading control. Densitometric analysis of LMP2, LMP7 and STAT1 expression normalized to β‐actin loading control in pmAT2 from three independent pmAT2 preparations.MHC‐I H‐2K^b^ surface expression on pmAT2 cells treated as in A. Densitometric analysis shows mean ± SEM of three different pmAT2 preparations from 6–8 mice each.Representative Western blot analysis of phLF for LMP2, LMP7, STAT1, and β‐actin (loading control) upon transfection of mtDNA or mtDNA pretreated with DNase I for 24 h, 48 h or 72 h.Representative Western blot detecting LMP2, LMP7, and STAT1 in phLF transfected with cGAMP for 24 h. β‐actin was used as a loading control.HLA‐A2 surface expression of phLF transfected with cGAMP, mtDNA, mtDNA pretreated with DNase I, or DNase I (*n* = 3).HLA‐A2 surface expression upon treatment of phLFs with IFNβ or IFNγ for 48 h (*n* = 3).Western blot analysis of STING, pTBK1, TBK1, pIRF3, IRF3, pIκBα, IκBα, pP65, P65 and β‐actin (loading control) in phLFs transfected with mtDNA for 2 h, 6 h, or 24 h, respectively.RT‐qPCR analysis of *IFNB* in phLF transfected with 200 ng/ml human mtDNA isolated from healthy primary skin fibroblasts for 24 h.Western blot analysis of LMP2, LMP7 and β‐actin (loading control) in neonatal skin fibroblasts of a healthy donor (WT), in adult skin fibroblasts of a donor with a heterozygous STAT1 loss of function mutation (STAT1*^+/−^) and in neonatal skin fibroblasts of a child carrying the same STAT1 LOF mutation (STAT1*^−/−^) on both alleles. Cells had been transfected with 200 ng mtDNA for 48 h. Data information: Data are shown as mean ± SEM of three independent experiments using different passages of the same cells and are presented as fold over mean of control. All data were analyzed with two‐tailed unpaired Student's *t*‐test. Asterisks indicate significance as *P < 0.05, **P < 0.001, ***P < 0.0001. Source data are available online for this figure.

**Figure EV5:**
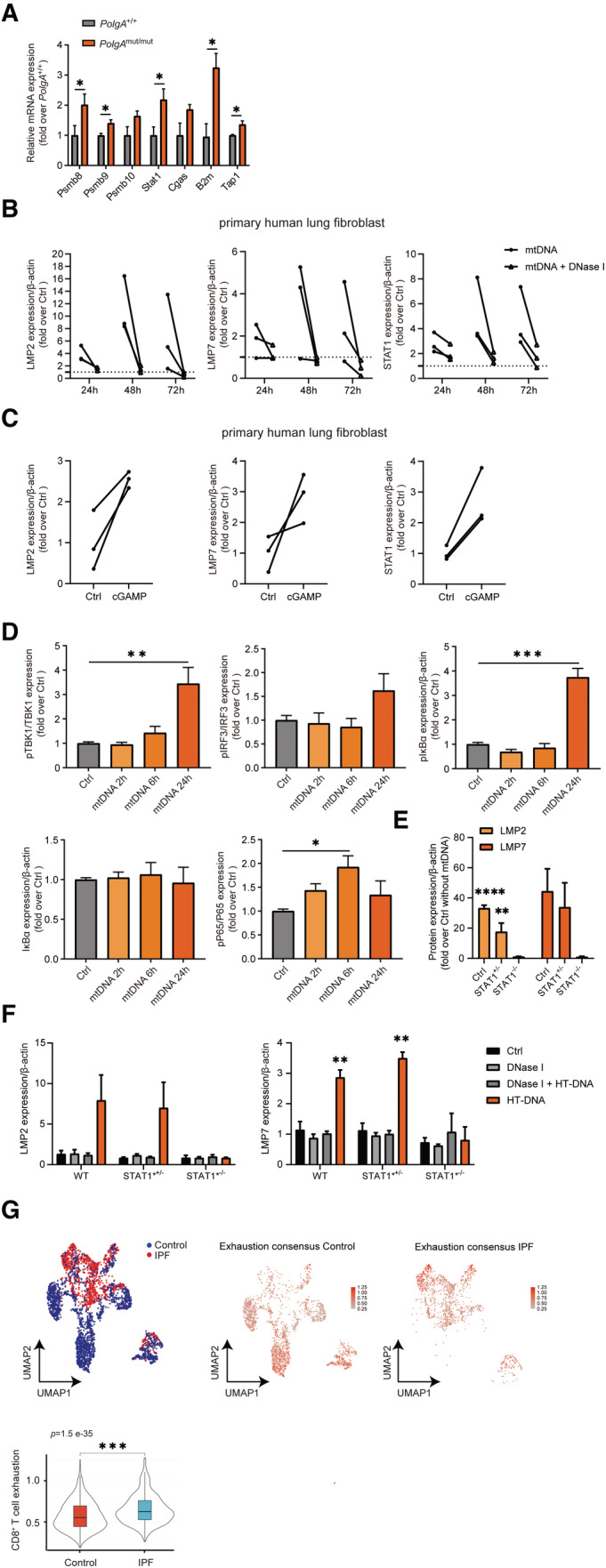
Induction of the immunoproteasome in lung cells upon mtDNA stress RT‐qPCR analysis of immunoproteasome subunits *Psmb8*, *Psmb9*, *Psmb10*, and of *Stat1*, *Cgas*, *B2m* and *Tap1* mRNA expression in *PolgA* ^+/+^ and *PolgA* ^mut/mut^ mice lungs (3–4 *PolgA* ^+/+^ and *PolgA* ^mut/mut^ mice).Densitometric analysis of LMP2, LMP7, and STAT1 expression normalized to β‐actin loading control in phLFs as exemplarily shown in the Western blot in Fig 6C using three different phLF cell lines isolated each from different donors. Values are given as fold over control with the respective control signal set to 1 and indicated by the dashed line.Densitometric analysis of LMP2, LMP7, and STAT1 expression normalized to β‐actin loading control in phLFs as exemplarily shown in Western blot in Fig 6D. Values are given as fold over control normalized to the mean of the controls.Densitometric analysis of Western blots for STING, pTBK1, TBK1, pIRF3, IRF3, pIκBα, IκBα, pP65, P65 and β‐actin (loading control) in phLFs transfected with mtDNA for 2 h, 6 h, or 24 h, respectively as exemplarily shown in Western blot in Fig 6G. Densitometric analysis shows mean ± SEM of three independent experiments using one phLF line and are presented as fold over mean of control.Densitometric analysis of Western blots (Fig 6I) for LMP2 and LMP7 in neonatal wild‐type (WT), adult heterozygous STAT1 mutant (STAT1*^+/−^) and neonatal homozygous STAT1 mutant (STAT1*^−/−^) skin fibroblasts transfected with mtDNA. Fold over Ctrl without transfection is shown, respectively (*n* = 3 independent biological experiments).Densitometric analysis of Western blots (Fig 8C) for LMP2 and LMP7 in skin fibroblast transfected with HT‐DNA or HT‐DNA pretreated with DNase I, respectively (*n* = 3 independent biological experiments).UMAP and violin plot analysis of scRNA seq T‐cell exhaustion gene signature in control and IPF CD3^+^CD8^+^ T cells using the publicly available data set GSE135893. scRNA seq data from 24 IPF patients and 10 donors as controls were analyzed with two‐tailed unpaired Student's *t*‐test. The violin plots represent T cell exhaustion consensus enrichment score density and distribution. Elements in boxplot: Upper and lower whisker = max and least value excluding outliers, box edges the first and third quartiles, boxplot midline represents the median. Data information: All data are shown as mean ± SEM and were analyzed with two‐tailed unpaired Student's *t*‐test. Asterisks indicate significance as *P < 0.05, **P < 0.01, ***P < 0.001, ****P < 0.0001.

Mitochondrial dysfunction, mtDNA release, and activation of cGAS/STING signaling are known pathomechanistic features of pulmonary fibrosis (Liu *et al*, 2014; Bueno *et al*, 2015; Mora *et al*, 2017; Benmerzoug *et al*, 2018). We thus investigated whether mtDNA stress activates the immunoproteasome/MHC class I pathway in the lung. In lungs of *PolgA* ^mut/mut^ mice, we observed concerted upregulation of the immunoproteasome subunits *Psmb8‐10*, *Stat1*, the MHC class I‐associated β2‐microglobulin (*B2m*) and the MHC class I peptide transporter *Tap1* when compared to wild‐type *PolgA* ^*+/+*^ lung tissue suggesting that the immunoproteasome/MHC class I pathway is active in lungs of *PolgA* ^mut/mut^ mice (Fig EV5A). We next isolated primary alveolar type 2 epithelial cells (AT2) from lungs of wild‐type mice and tested whether mtDNA stress can directly activate the immunoproteasome/MHC class I pathway in lung epithelial cells. Transfection of mtDNA into primary AT2 cells upregulated the expression of the immunoproteasome and STAT1 (Fig 6A). This effect was abrogated by DNase I treatment (Fig 6A). The expression of the MHC‐I H‐2K^b^ allele was also induced as determined by flow cytometry of primary mouse AT2 cells but abolished by DNase I treatment (Fig 6B). Immunoproteasome, STAT1, and surface expression of MHC class I were similarly induced when treating primary AT2 cells with IFNβ (Fig 6A and B).

As a second model for lung cells, we used primary human lung fibroblasts (phLF), isolated from healthy lung explant donors. Transfection of mtDNA into phLF also upregulated the immunoproteasome subunits LMP2 and LMP7 as well as STAT1 with a peak expression after 48‐h transfection (Figs 6C and EV5B). Pretreatment of mtDNA with DNase I abolished this effect (Figs 6C and EV5B). Moreover, direct activation of the cGAS/STING signaling pathway by transfection of the STING ligand cGAMP induced LMP2, LMP7, and STAT1 expressions within 24 h (Figs 6D and EV5C). Transfection of mtDNA or cGAMP concomitantly upregulated surface expression of the MHC‐I HLA‐A2 allele in phLF (Fig 6E) that was abolished by DNase I treatment (Fig 6E). HLA‐A2 was similarly induced by IFNβ and IFNγ (Fig 6F). We also confirmed activation of the cGAS/STING pathway involving NFκB but not IRF3 signaling after 6–24 h upon mtDNA transfection into primary human lung cells (Figs 6G and EV5D) resulting in pronounced induction of IFNβ (Fig 6H). Using skin fibroblasts derived from a newborn patient with a homozygous STAT1 loss‐of‐function (LOF) mutation, we compared induction of the immunoproteasome by mtDNA transfection in these STAT1 signaling‐deficient cells with neonatal healthy cells (WT) and adult skin fibroblasts obtained from one of the parents that carried a heterozygous STAT1 mutation. As shown in Fig 6I, mtDNA‐mediated upregulation of the immunoproteasome was completely abrogated in STAT1 LOF cells (Figs 6I and EV5E), demonstrating the essential nature of the STAT1 signaling pathway for the adaptive type I interferon response.

### mtDNA stress directly activates influenza specific CD8^+^ T cells

**Figure 7:**
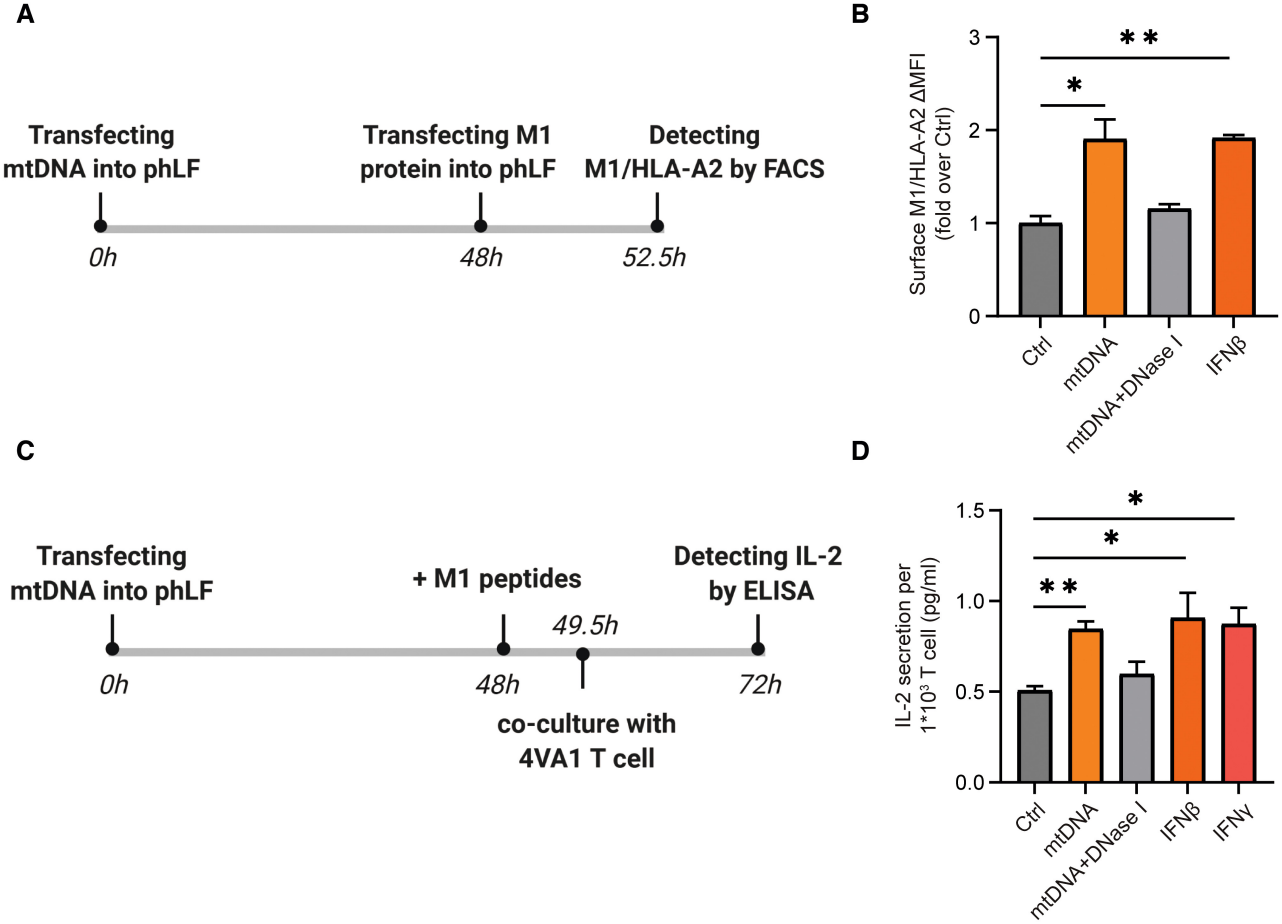
mtDNA transfection activates Influenza A‐specific CD8^+^ T cells Scheme of the immunoproteasome‐specific T cell assay detecting the HLA‐A2 restricted GILGFVFT peptide in phLF.Flow cytometric detection of Influenza‐M1/HLA‐A2 complex in phLF upon transfection with mtDNA, mtDNA pretreated with DNase I, or treatment with IFNβ (*n* = 3 independent experiments).Scheme of M1/HLA‐A2 restricted CD8^+^ T cells (4VA1 T cell clone) activated by mtDNA stressed phLF that had been loaded with Influenza‐M1 peptide.CD8^+^ T‐cell activation assay of Influenza‐M1/HLA‐A2 restricted CD8^+^ T cells upon co‐culture with mtDNA‐transfected phLFs loaded with M1 peptide or upon treatment with IFNβ or IFNγ displaying secretion of IL‐2 from three different biological experiments. Data information: All data are shown as mean ± SEM and analyzed with one‐way ANOVA test. Asterisks indicate significance as *P < 0.05, **P < 0.01. Source data are available online for this figure.

To assay whether mtDNA stress activates CD8^+^ T cells in human lung cells, we employed an immunoproteasome‐specific T‐cell assay similar to the above‐described UTY assay. The matrix M1 protein on Influenza A virus (IAV) is specifically cleaved by the immunoproteasome to generate the HLA‐A2 restricted GILGFVFT peptide and activates interleukin (IL)‐2 secretion of an influenza M1‐specific CD8^+^ T‐cell clone (Canaday *et al*, 2003). We thus introduced the influenza M1 protein into mtDNA‐transfected primary human lung cells and quantified presentation of the HLA‐A2‐restricted M1 epitope on the cell surface by flow cytometry using an antibody specific for HLA‐A2 bound M1 peptide (Fig 7A; Biddison *et al*, 2003). mtDNA transfection activated the presentation of the immunoproteasome‐specific M1 peptide on HLA‐A2 to a similar extent as stimulation of lung cells with IFNβ but was abolished by DNase I treatment (Fig 7B). Moreover, IAV‐specific CD8^+^ T cells were activated by mtDNA‐transfected lung cells when the M1 peptide was directly loaded onto HLA‐A2 molecules (Fig 7C and D). IL‐2 secretion of CD8^+^ T cells was activated by almost twofold, very similar to the treatment of cells with IFNβ or IFNγ (Fig 7D). Our data thus demonstrate that intracellular mtDNA sensing activates adaptive CD8^+^ T‐cell responses in primary human lung cells involving the immunoproteasome and MHC class I antigen presentation pathway. This adaptive type I interferon response to intracellular mtDNA is conserved in mouse and human parenchymal cells.

### Double‐stranded DNA sensing via cGAS/STING activates an adaptive type I interferon response

**Figure 8:**
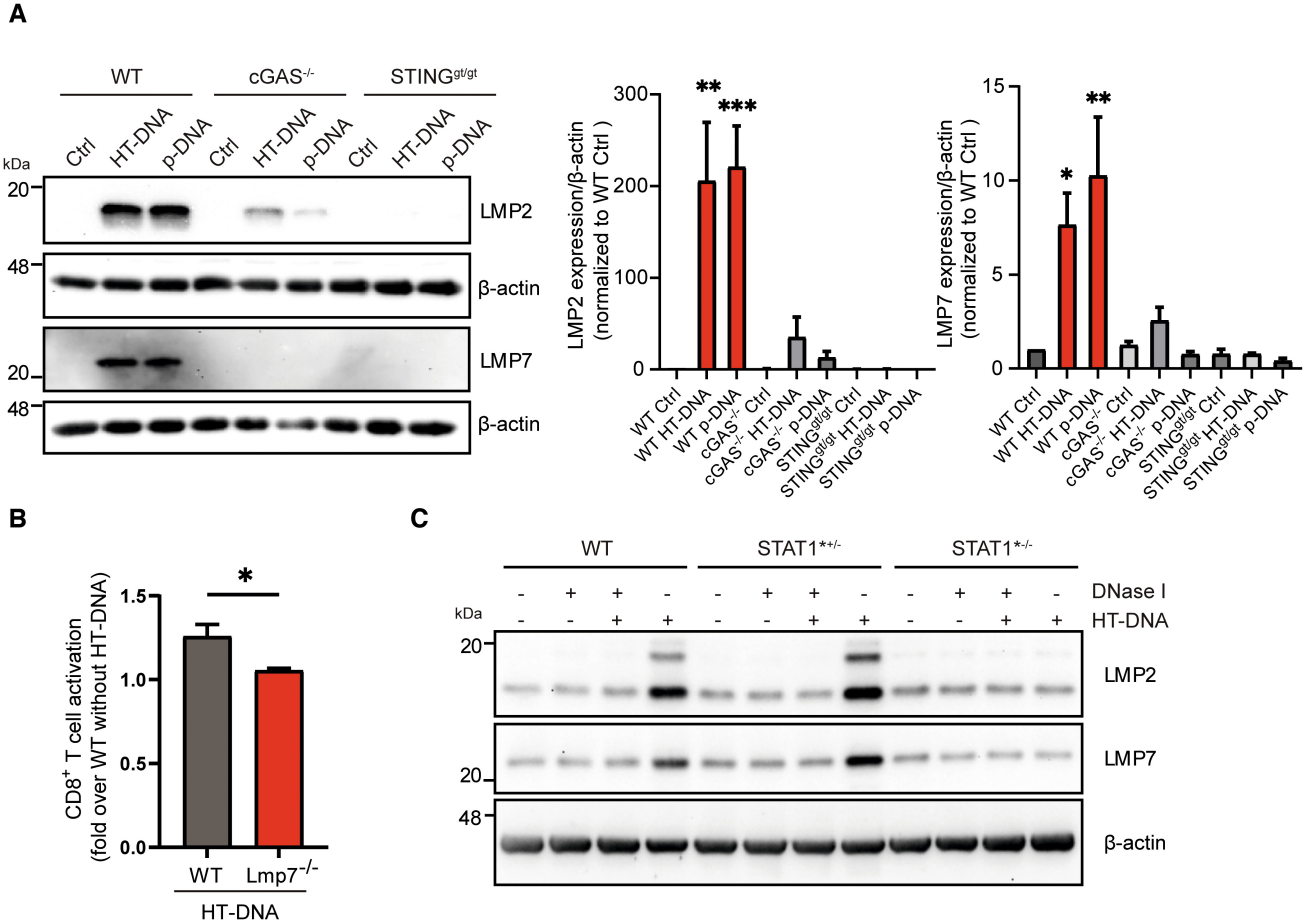
Double‐stranded DNA sensing via cGAS/STING activates an adaptive type I interferon response Western blot analysis of the immunoproteasome subunits LMP2 and LMP7 in wild‐type (WT), cGAS KO and STING^gt/gt^ loss‐of‐function MEFs transfected with 1 μg/ml HT‐DNA or plasmid‐DNA for 48 h. β‐actin was used as a loading control. Densitometric analysis of LMP2 and LMP7 expression normalized to β‐actin loading control in wild‐type (WT) controls (ctrl) from three independent experiments.UTY‐assay of CD8^+^ reporter T cells upon 1:2 co‐culture (splenocytes:T cells) with splenocytes isolated from either wildtype (WT) or LMP7 KO mice and transfected with htDNA. Splenocytes were isolated from 4 mice each in two different preparations.Western blot analysis of immunoproteasome subunits LMP2 and LMP7 in wild‐type (WT), heterozygous STAT1 mutated (STAT1*^+/−^) and homozygous STAT1 mutated (STAT1*^−/−^) skin fibroblasts transfected with 1 μg/ml HT‐DNA or pretreated HT‐DNA with DNase I for 48 h. Data information: Data are shown as mean ± SEM of at least three independent experiments and were analyzed with two‐tailed unpaired Student's *t*‐test. Asterisks indicate significance as *P < 0.05, **P < 0.01, ***P < 0.001. Source data are available online for this figure.

We next asked the question whether induction of the adaptive type I interferon response is restricted to intracellular mtDNA or whether it also applies to the presence of cytoplasmic double‐stranded DNA in general. Transfection of genomic or plasmid DNA into MEFs of wild‐type mice markedly upregulated both LMP2 and LMP7 (Fig 8A). This dsDNA‐mediated induction of the immunoproteasome was almost completely abrogated in cGAS KO and STING‐mutant cells (Fig 8A). Transfection of dsDNA into splenocytes that had been isolated from male wild‐type mice also activated autoreactive CD8^+^ UTY_264‐254_ reporter T cells (Fig 8B). CD8^+^ T‐cell activation, however, was lost in splenocytes of male LMP7 KO mice (Fig 8B) confirming the crucial role of the immunoproteasome in adaptive type I interferon responses. We further tested the involvement of STAT1 signaling our STAT1 LOF cells. Of note, dsDNA‐mediated upregulation of the immunoproteasome subunits LMP2 and LMP7 was abrogated in these STAT1 LOF cells compared with WT controls and the adult skin fibroblasts of the heterozygous carrier of the same mutation (Figs 8C and EV5F). Our data thus demonstrate that not only mtDNA but double‐stranded DNA in general activates a cGAS/STING‐dependent adaptive immune response that involves upregulation of the immunoproteasome.

### Immunoproteasome and CD8^+^ T cells are activated in pulmonary fibrosis

**Figure 9:**
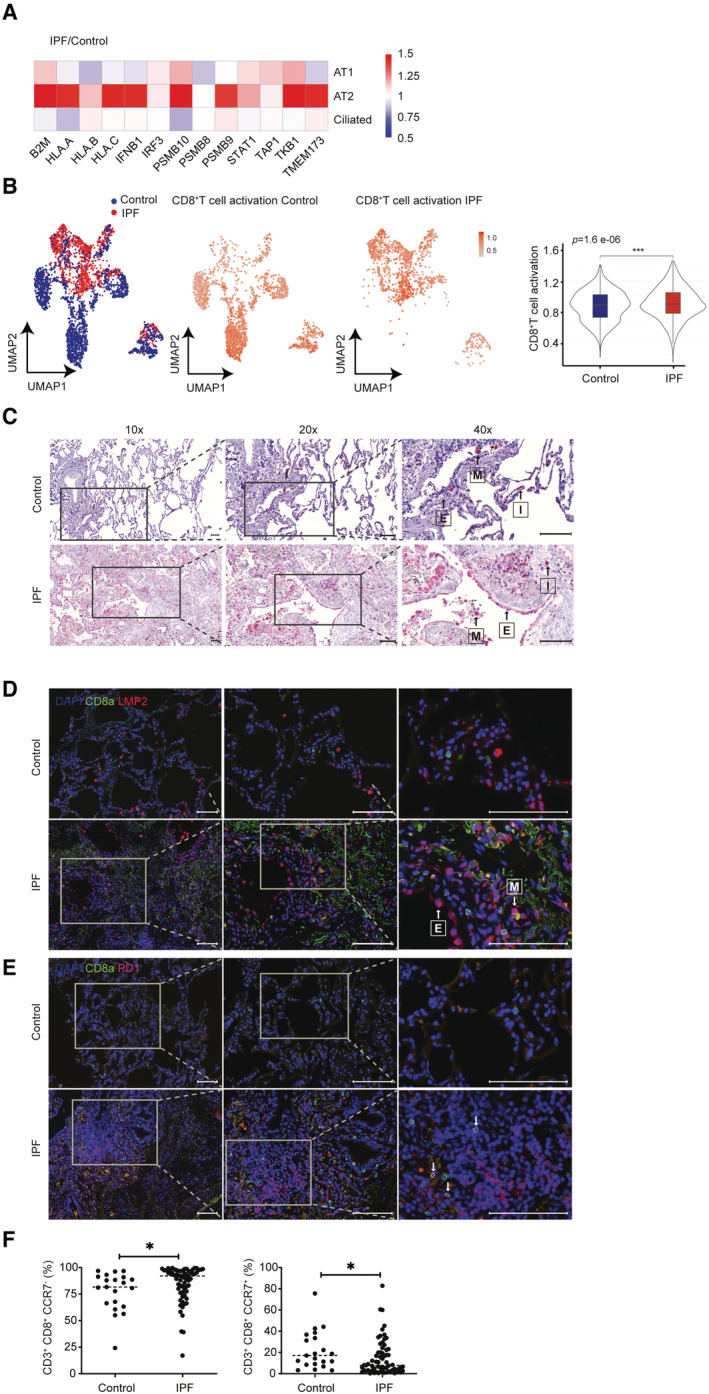
Immunoproteasome and CD8^+^ T cells are activated in pulmonary fibrosis AComparison of the log2‐fold changes in mRNA expression in AT1, AT2, and ciliated cells of IPF patients relative to control using the publicly available data set GSE135893.BUMAP and violin plot analysis of scRNA seq T‐cell activation gene signature in control and IPF CD3^+^CD8^+^ T cells using the publicly available data set GSE135893. Data are presented as gated % of parent. scRNA seq data from 24 IPF patients and 10 donors as controls were analyzed with two‐tailed unpaired Student's *t*‐test. The violin plots represent T cell activation signature enrichment score density and distribution. Elements in boxplot: Upper and lower whisker = max and least value excluding outliers, box edges the first and third quartiles, boxplot midline represents the median. Asterisks indicate significance as ***P < 0.001.CRepresentative immunohistochemistry staining of LMP2 in control and IPF lungs; M, E and I represent Macrophages, Epithelium and Interstitium, magnifications 10×, 20× and 40× correspond to a scale bar of 100 μm, 100 μm and 50 μm, respectively.D, ERepresentative immunofluorescence detection of LMP2 (red), CD8^+^ (green) and PD‐1 (red) in control and IPF lungs, arrows indicate double positive CD8^+^ PD‐1^+^ cells, magnifications 10×, 20× and 40× correspond to a scale bar of 100 μm. Arrows indicate epithelial cells (E) or macrophages (M) and cells stained positive for CD8.FFlow cytometric analysis of CD3, CD8, and CCR7 expression on peripheral blood mononuclear cells (PBMCs) of control (*n* = 21) and IPF (*n* = 66) patients. Data are presented as gated % of parent. Data were analyzed with two‐tailed unpaired Student's *t*‐test. Asterisks indicate significance as *P < 0.05. Source data are available online for this figure.

We finally investigated the disease relevance of our findings in patients with idiopathic pulmonary fibrosis (IPF). Using publicly available single‐cell RNA sequencing (scRNA seq) datasets (www.ipfcellatlas.com), we observed specific activation of the cGAS/STING‐induced adaptive immune response in alveolar type 2 (AT2) epithelial cells of IPF patients (Fig 9A). Specifically, the immunoproteasome subunits *PSMB9* (LMP7) and *PSMB10* (MECL1), *TMEM173* (STING), mediators of type I interferon signaling such as *STAT1*, *TBK1*, and *IFNB1* and several MHC I alleles (*HLA‐A*, *HLA‐C*) were uniformly upregulated. Of note, we detected concomitant CD8^+^ T‐cell activation in IPF lung tissue (Fig 9B): UMAP (Uniform Manifold Approximation and Projection) analysis of CD8^+^ T‐cell activation gene signatures (Table EV2) in CD3^+^CD8^+^ T‐cell populations revealed a distinct and significant activation pattern of CD8^+^ T cells in fibrotic lungs of IPF patients (Fig 9B). CD8^+^ T‐cell activation in IPF lungs overlapped with significantly elevated expression of T‐cell exhaustion markers (Fig EV5G and Table EV3 for gene signature) suggesting chronic CD8^+^ T‐cell activation that possibly results in immune cell exhaustion.

Induction of the immunoproteasome in lung epithelial cells of IPF patients was confirmed by immunohistochemical and immunofluorescence staining of fibrotic lung tissue (Fig 9C and D). While the expression of the immunoproteasome subunit in healthy lung tissue was restricted to immune cells such as alveolar macrophages and interstitial immune cells, fibrotic lungs also displayed prominent expression of the immunoproteasome subunit LMP2 in hyperplastic lung epithelial cells overlying fibroblast foci (Fig 9C and D). These sites of fibrotic remodeling and defective re‐epithelization are pathological hallmarks of IPF (Renzoni *et al*, 2021). IPF lungs also displayed infiltration of single positive CD8^+^ T cells and PD‐1^+^ cells, as well as double positive cells that further support the concept of chronic T‐cell activation and their subsequent exhaustion (Pritykin *et al*, 2021) (Fig 9E).

Of note, the activation of CD8^+^ T cells in IPF patients was also detectable on a systemic level when analyzing peripheral blood cells from 69 IPF patients and controls of the Munich IPF cohort (Fernandez *et al*, 2016). While CD8^+^ noneffector T cells (CD3^+^ CD8^+^ CCR7^+^) were reduced in IPF patients, the numbers of CD8^+^ effector T cells (CD3^+^ CD8^+^ CCR7^−^) were significantly elevated (Fig 9F). These data thus point towards the activation of potentially autoreactive CD8^+^ T cells in IPF that represents a novel potential pathomechanism for pulmonary fibrosis and opens new therapeutic perspectives.

## Discussion

We here provide first evidence for the induction of the immunoproteasome by cytoplasmic double‐stranded DNA via the cGAS/STING pathway that drives the activation of CD8^+^ T cells. Moreover, our data indicate chronic activation of type I interferon signaling and the immunoproteasome in alveolar epithelial cells of IPF patients that concurs with activation of lung resident and systemic CD8^+^ T cells in IPF. These data raise the possibility that cytoplasmic DNA‐induced activation of type I interferon signaling contributes to the upregulation of the immunoproteasome/MHC class I pathway in IPF alveolar epithelial cells to potentially activate autoimmune T‐cell responses. This study thus provides first evidence for a CD8^+^ T cell‐driven pathomechanism for IPF.

### Activation of the immunoproteasome and adaptive immune responses by cytoplasmic DNA

Under physiological conditions, immunoproteasome expression is low or absent in parenchymal cells but can be rapidly induced upon stimulation of cells with IFNγ, TNFα, or LPS (Kammerl & Meiners, 2016). Transcriptional induction involves the activity of the pro‐inflammatory transcription factor STAT1 (Barton *et al*, 2002). The immunoproteasome can also be induced by IFNα and IFNβ upon infection with the RNA hepatitis C virus (HCV) *in vitro* and *in vivo* (Shin *et al*, 2006). We here demonstrate that IFNβ activates the immunoproteasome in response to cytoplasmic double‐stranded DNA sensing, such as mtDNA and genomic DNA, via the cGAS/ STING pathway. Induction of the immunoproteasome has also recently been observed upon mtDNA depletion in breast carcinoma cells by unbiased RNA seq analysis but was not observed in other models of mitochondrial stress such as mtDNA heteroplasmy (Picard *et al*, 2014) or heterozygous TFAM knockout (West *et al*, 2015; Wu *et al*, 2019). In cells treated with rotenone—a well‐known blocker of respiratory chain complex I—the immunoproteasome was activated as part of a cytotoxic response due to severe oxidative stress (Sun *et al*, 2019, 2020). By contrast, our study demonstrates induction of the immunoproteasome by mtDNA in *PolgA* ^mut/mut^ cells, upon TFAM silencing and upon transfection of mtDNA into the cells. Moreover, we here show activation of the immunoproteasome by transfection of double‐stranded DNA in general. These experimental systems are characterized by mitochondrial dysfunction and mtDNA stress in the absence of oxidative stress. We can thus rule out oxidative stress as an inducer of the immunoproteasome.

Importantly, the induction of the immunoproteasome in response to cytoplasmic DNA was accompanied by concomitant upregulation of the MHC class I antigen presentation pathway and resulted in the activation of cytotoxic CD8^+^ T cells as demonstrated in mouse and human cells using two different CD8^+^ T‐cell activation assays. We thereby provide first evidence that type I interferons cell‐intrinsically prime CD8^+^ T cells against stressed parenchymal cells. This response depended on the activation of the cGAS/STING/type I interferon signaling pathway as unambiguously demonstrated by cGAS and STING gene silencing and knockout, antibody‐mediated blocking of type I interferon signaling and the use of patient‐derived skin fibroblasts containing a STAT1 loss‐of‐function mutation. Importantly, the here described cGAS/STING‐induced adaptive immune response is different from the well‐known activity of type I interferons to activate innate immune signaling in response to cGAS/STING‐ intracellular nucleic acid sensing of viral DNA or cytoplasmic double‐stranded DNA (Hornung *et al*, 2014; Margolis *et al*, 2017; West & Shadel, 2017; Ablasser & Chen, 2019; Miller *et al*, 2021). Activation of adaptive T‐cell responses via cGAS/STING/type I interferon signaling has previously been demonstrated in several models of antitumor immunity, graft‐versus‐host disease, autoimmune and inflammatory diseases (Corrales *et al*, 2015; Bader *et al*, 2020; Zhang *et al*, 2020; Hu *et al*, 2021). In these studies, however, adaptive immune responses were always described as an indirect activity of type I interferons which act as co‐stimulatory signals for CD8^+^ T‐cell activation.

### CD8^+^ T cell activation in IPF—evidence for autoimmunity

We here provide evidence for a chronic activation of type I interferon‐induced adaptive immune signaling in alveolar epithelial cells of IPF patients that concurs with local and systemic CD8^+^ T‐cell activation. While we cannot delineate whether this chronic activation is due to mtDNA stress or genomic DNA sensing, we speculate that this chronic type I interferon signaling in lung epithelial cells promotes a break in immune tolerance with activation of autoreactive CD8^+^ T‐cell responses, thereby contributing to autoimmune‐driven tissue remodeling and fibrosis. Indeed, chronic activation of type I interferon signaling is an established feature of autoimmune diseases (Crow *et al*, 2019). Moreover, it is also a prominent feature of persistent virus infections that cause fibrotic tissue remodeling (Teijaro *et al*, 2013; Oldstone, 2015) and has recently been rediscovered in the course of the COVID‐19 pandemic (Greene & Zuniga, 2021; Domizio *et al*, 2022). As prolonged type I interferon signaling has recently been shown to disrupt epithelial repair (Major *et al*, 2020), chronic activation of this pathway in lung epithelial cells of IPF patients may contribute to aberrant wound healing responses of chronically damaged alveolar epithelial cells in IPF (Fernandez & Eickelberg, 2012; Selman & Pardo, 2020). Importantly, type I interferon signaling is activated in response to multiple stressors such as mitochondrial and genomic DNA stress but also telomere dysfunction, which are hallmarks of IPF pathogenesis (Mora *et al*, 2017). Recent data on silica‐ and SARS‐CoV2‐induced lung damage reported on the key role of the cGAS/STING/type I interferon signaling axis (Benmerzoug *et al*, 2018; Domizio *et al*, 2022). cGAS/STING signaling is also activated by dysfunctional telomeres (Abdisalaam *et al*, 2020) that might link telomere dysfunction as found in sporadic and hereditary IPF to development of pulmonary fibrosis (Cronkhite *et al*, 2008; Armanios & Blackburn, 2012). It is thus tempting to speculate that all these stressors, that is, ER and mtDNA stress, telomere dysfunction as well as persistent virus infection converge on chronic activation of type I interferon signaling in lung epithelial cells, thereby driving autoimmune IPF pathogenesis.

Our data on activated CD8^+^ T cells in IPF lungs indicate that at some point of IPF pathogenesis, these T cells experienced some MHC class I antigen/T‐cell receptor (TCR) interaction that served as signal 1 and together with a co‐stimulation signal 2 as well as additional inflammatory cytokine signaling to promote their activation and proliferation. While we do not know the antigenic specificity of the CD8^+^ T cells in the IPF tissue, antigen recognition is essential for CD8^+^ T‐cell activation. Potential antigenic sources can be viral antigens as observed in chronic infections (McKinney *et al*, 2015). CD8^+^ T‐cell activation might also be caused by molecular mimicry, that is, the cross‐reactivity of antiviral cytotoxic CD8^+^ T cells to autoantigens and environmental bacterial antigens (Oldstone, 1998). The cytotoxic response to these antigens is much weaker than the initial viral antigens but causes a persistent low‐level activation of autoreactive CD8^+^ T cells (Misko *et al*, 1999) as for example shown for Psoriasis, type I diabetes and recently for multiple sclerosis and age‐dependent neuronal degeneration (Valdimarsson *et al*, 2009; Campisi *et al*, 2022; Girdhar *et al*, 2022; Zhou *et al*, 2022). A chronic inflammatory milieu such as in IPF lungs will most probably contribute to expansion of reactive T‐cell clones and activation of their effector functions.

Indeed, multiple studies suggest a crucial role of innate and adaptive immune responses in IPF disease onset and progression (Wynn, 2011; Heukels *et al*, 2019). CD8^+^ T cells are abundantly present in lung tissue of patients with early or late stages of IPF and have been implicated in fatal IPF disease outcome (Daniil *et al*, 2005; Xu *et al*, 2021). CD8^+^ T cell‐mediated immune responses are also crucial for the development of lung fibrosis upon experimental virus infection (O'Flaherty *et al*, 2015; Wang *et al*, 2019) suggesting a major and currently underestimated role for this T‐cell type in IPF. Moreover, expression of PD‐1 and CD8^+^‐positive T cells suggests that immune activation and possibly exhaustion is taking place in IPF lungs. While upregulation of PD‐1 has been demonstrated to restrict fibrotic remodeling and type I interferon tissue damage in experimental mouse models (Raju *et al*, 2019; Wang *et al*, 2019), it might represent a frustrated attempt to counteract autoimmune CD8^+^ T‐cell activation in end‐stage IPF lungs (McKinney *et al*, 2015; Pritykin *et al*, 2021). Moreover, a recent study also provides first evidence that type I interferons facilitate induction of immune checkpoint molecules and regulate T‐cell function (Sumida *et al*, 2022) further supporting our concept of type I interferon‐induced adaptive immune response.

Concluding, we here demonstrate a first link between aberrant type I interferon signaling in alveolar epithelial cells and activation of CD8^+^ T‐cell immune responses in pulmonary fibrosis. This pathophysiological concept may also underlie fibrotic lung remodeling in patients with long‐COVID‐19 and may offer new therapeutic options for treatment of IPF.

## Materials and Methods

### Cell culture

Mouse embryonic fibroblasts were cultured in DMEM High Glucose medium supplemented with 10% fetal bovine serum (FBS; Sigma‐Aldrich), 4 mM L‐Glutamine (Thermo Fisher Scientific), and 100 IU/ml penicillin/streptomycin (Thermo Fisher Scientific) at 37°C and 5% CO_2_. MEFs were isolated from three wild‐type mice denoted as *PolgA* ^*+/+*^ MEFs and three PolgA^mut^ homozygous knock‐in mice denoted as *PolgA* ^mut/mut^ MEFs and used as different biological replicates. Mouse embryonic fibroblasts were obtained from cGAS knockout mice (cGAS^−/−^) and the STING Goldenticket (gt) mouse strain (STING^gt/gt^) that contains a I199N missense mutation resulting in a null‐phenotype with no detectable STING activity. These MEFs were kindly provided by Qi Chen from Fujian Normal University.

Cultivation of splenocytes and UTY CD8^+^ T cells was carried out as described previously (Kammerl *et al*, 2016). Primary human skin fibroblasts were obtained from the laboratory of Fabian Hauck at the Ludwig‐Maximilians‐University. The STAT1 homozygous mutation is a c.1011_1012delAG mutation resulting in loss of function (Boehmer *et al*, 2020). The STAT1 heterozygous mutation skin fibroblasts were derived from the father of the diseased child and carries the same mutation. Different passages (P5‐7) were used for the experiments. Cells were cultured in DMEM High Glucose medium supplemented with 10% fetal bovine serum (FBS; Sigma‐Aldrich), 4 mM L‐Glutamine (Thermo Fisher Scientific) and 100 IU/ml penicillin/streptomycin (Thermo Fisher Scientific) and 200 μM Uridine (Sigma‐Aldrich) as described before (Boehmer *et al*, 2020). Primary human lung fibroblasts (phLF) were isolated from peripheral lung tissue from healthy donors and used as different biological replicates (ID: 406, 409Sp, 423g). phLFs were cultured in MCDB131 medium supplemented with 10% fetal bovine serum (FBS) (Sigma‐Aldrich), 4 mM L‐Glutamine (Thermo Fisher Scientific), 100 IU/ml penicillin/streptomycin (Thermo Fisher Scientific), 5 μg/ml insulin (Thermo Fisher Scientific), 0.5 ng/ml human recombinant EGF (Sigma‐Aldrich), and 2 ng/ml human recombinant β‐FGF (Thermo Fisher Scientific) as described before (Welk *et al*, 2019). Ethical approval for use of human cells was obtained by the respective institutions as specified in the original study publications (Semren *et al*, 2015; Boehmer *et al*, 2020).

Primary mouse AT2 cells were cultured in DMEM medium supplemented with 3.6 mg/ml glucose, 10% fetal bovine serum (FBS; Sigma‐Aldrich), 4 mM L‐glutamine (Thermo Fisher Scientific), 100 IU/ml penicillin/streptomycin (Thermo Fisher Scientific), 2.5 μg/ml amphotericin B. UTY‐specific T cells were cultured with IMDM with 10% FCS and 100 IU/ml penicillin/streptomycin (Thermo Fisher Scientific). Influenza M1‐specific 4VA1 T cells were kindly provided by David Canaday (Case Western Reserve University, Cleveland, Ohio, USA), cultured in DMEM High glucose medium supplemented with 10% fetal bovine serum (FBS; Sigma‐Aldrich), 4 mM L‐glutamine (Thermo Fisher Scientific), 100 IU/ml penicillin/streptomycin (Thermo Fisher Scientific), 1% HEPES (Sigma‐Aldrich), 1% non‐essential amino acid (Euroclone) and 25 μM 2‐mercaptoethanol (Thermo Fisher Scientific).

Conditioned medium was obtained from *PolgA* ^mut/mut^ MEFs. For that *PolgA* ^mut/mut^ MEFs and *PolgA* ^*+/+*^ MEFs were seeded into six‐well plates and cell‐free medium was collected after 48 h by 2,138 *g* centrifugation for 5 min. The conditioned medium from *PolgA* ^mut/mut^ MEFs was diluted 1:1 with medium from *PolgA* ^*+/+*^ MEFs and cells were cultured for further 48 h until harvesting. Similarly prepared conditioned medium obtained from *PolgA* ^*+/+*^ MEFs was used as a control.

### Isolation of primary mouse AT2 cells

Primary AT2 (pmAT2) cells were isolated from C57BL6/N mice aged 6–12 weeks as previously described (Lehmann *et al*, 2020). Mice were anesthetized by mixed (1:1) ketamin (Medistar Arzneimittelvertrieb)/xylazine (Bela‐pharm) and heparin (Ratiopharm). After flushing out the blood from the lungs with DPBS (Thermo Fisher Scientific) via the right heart, lungs were filled with dispase (50 units/ml, Corning) and low gelling temperature agarose (2%, Sigma Aldrich). The harvested lung lobes were minced in DMEM medium containing 0.04 mg/ml DNase I (AppliChem). The cell suspension was then sequentially filtered using 100 μm, 20 μm, 10 μm sized nylon meshes (Sefar). Fibroblasts in the filtered cell suspension were removed via adherence to noncoated plastic plates. Afterwards, the cell suspension was depleted of CD45^+^ and CD31^+^ cells using the respective antibody‐coupled magnetic beads (Miltenyi Biotec) according to the manufacturer's instructions. Isolated pmAT2 cells were seeded in either six‐well plates (2 × 10^6^ cells per well) or 12‐well plates (1 × 10^6^ cells per well) and cultured for 72 h as described above.

### Cell transfection

Transfection of siRNA, cGAMP, mtDNA, herring testis (HT), or plasmid DNA was performed using RNAiMax according to the maunfacturer's instructions (Invitrogen). mtDNA was isolated from mitochondria using the Mitochondrial Isolation Kit for Mammalian cells (Thermo Fisher Scientific) and mtDNA was purified by QIAamp® DNA Mini Kit. Mouse mtDNA was prepared from *PolgA* ^*+/+*^ MEFs and human mtDNA was prepared from primary human skin fibroblasts. Herring Testes DNA (HT‐DNA) was purchased from Sigma‐Aldrich (D6898).

Mouse mtDNA, human mtDNA, or mtDNA that had been digested by 20 μg/ml DNase I (Sigma‐Aldrich) for 30 min at 37°C as DNAse I‐pretreated mtDNA, were transfected with RNAiMax for 48 h. 4 μg/ml cGAMP (Sigma‐Aldrich, 5.31889.0001) was transfected with RNAiMax for 24 h. Mouse mtDNA was transfected at a concentration of 100 ng/ml into MEFs or pmAT2. Human mtDNA was used at a concentration of 200 ng/ml for phLF transfection. siRNAs were transfected for 48 h at a final concentration of 5 nM. Details on the siRNAs applied in this study are provided in Table EV4. HT‐DNA or plasmid DNA was transfected at a concentration of 1 μg/ml.

### Protein extraction and BCA assay

For Western blot analysis, cells were lysed in RIPA buffer (50 mM Tris HCl, pH 7.5, 150 mM NaCl, 1% NP40, 0.5% sodiumdeoxycholate, 0.1% SDS). For that, cells were harvested from six‐well plate and pellets were lysed in RIPA buffer supplemented with protease inhibitor cocktail (Roche) and phosphatase inhibitor cocktail (Roche) for 20 min on ice. Total cellular proteins were recovered upon centrifugation at 18,531 *g* for 20 min at 4°C. For proteasome activity assays, proteins were extracted from cells using native lysis buffer (50 mM Tris HCl pH 7.5, 2 mM DTT, 5 mM MgCl_2_, 10% glycerol, 2 mM ATP, 0.05% digitonin) supplemented with protease inhibitor cocktail (Roche) for 20 min on ice and centrifuged at 18,531 *g* for 20 min at 4°C. Protein concentration in cell lysates was determined by Bradford assay using the BCA detection reagent according to the manufacturer's instructions (Thermo Fisher Scientific) and an albumin standard (Thermo Fisher Scientific).

### Western blot analysis

Twenty five microgram protein of RIPA lysates was mixed with 6× Laemmli buffer (1 M Tris HCl, pH 6.8, 15% glycerol, 6% SDS, 1% bromophenol blue) and incubated at 95°C for 5 min. Samples were then loaded onto 10–15% SDS–PAGE gels and run at 90–120 V for 60–90 min. Gels were blotted onto polyvinylidene difluoride membranes at 250 mA for 90 min on ice. For detecting phosphorylated proteins, blotting was performed at 300 mA for 2 h at 4°C. Membranes were blocked in Roti®‐Block (Carl Roth) for 1 h at room temperature and incubated with primary antibodies overnight at 4°C. Upon washing of the blots for 5 min with PBST (PBS, 0.1% Tween‐20) 3 times, blots were incubated with secondary antibodies at a dilution of 1:20,000 for 2 h at room temperature.

The following antibodies were used: Anti‐LMP2 (Abcam, ab3328), Anti‐LMP7 (Abcam, ab3329), Anti‐STAT1 (Cell Signaling Technology, #9172), Anti‐pSTAT1 (Y701) (Cell Signaling Technology, #7649), Anti‐TAP1 (Cell Signaling Technology, #12341), Anti‐cGAS (Cell Signaling Technology, #31659), Anti‐STING (Cell Signaling Technology, #50494), Anti‐pSTING (S365) (Cell Signaling Technology, #72971) Anti‐pIRF3 (S396) (Cell Signaling Technology, #29047), Anti‐IRF3 (Cell signaling technology, #4302), Anti‐pTBK1/NAK (S172) (Cell Signaling Technology, #5483), Anti‐TBK1/NAK (Cell Signaling Technology, #3504), Anti‐pIKBa (5A5, pS32/36) (Cell Signaling Technology, #9246), Anti‐IKBa (Cell Signaling Technology, #4814), Anti‐pP65 (931 t1, pS536) (Cell Signaling Technology, #3033), Anti‐P65 (Cell Signaling Technology, #8242), HRP‐conjugated‐anti‐β‐actin (Sigma‐Aldrich, a3854), Anti‐mouse IgG HRP‐linked (Cell Signaling Technology, #7076), and Anti‐rabbit IgG HRP‐linked (Cell Signaling Technology, #7074).

### Quantitative real‐time RT‐PCR

Total cellular RNA was isolated using the peqGOLD total RNA extraction kit according to the manufacturer's protocol (VWR Peqlab). Reverse transcription of 1 μg total RNA into cDNA was performed using M‐MLV reverse transcriptase (Sigma Aldrich). IFNβ signaling blocking experiments were carried out with 50,000 *PolgA* ^*+/+*^ or *PolgA* ^mut/mut^ cells seeded to six‐wells plates 24 h prior to the conduction of the experiment. Cells were then treated with the IFNAR‐1 antibody (Invitrogen, #16‐5945‐85, 0.5 μg/ml) for 48 h until lysis in TRIZOL (Thermo Fisher, 15596026) followed by robust vortexing after mixing with chloroform. This mix was then subjected to centrifugation at 17,105 *g* for 15 min at 4°C, and the clear supernatants were collected and mixed with isopropyl alcohol in a ratio of 1:1 for RNA precipitation. Upon centrifugation at 17,105 *g* for 10 min at 4°C, the RNA pellet was washed in 70% ethanol with 10,691 *g* for 5 min and dried RNA pellets were dissolved in RNAse‐free water. cDNAs were synthesized using the Maxima First Strand cDNA Synthesis Kit for RT‐qPCR (Thermo Fisher, K1642). Quantitative real‐time RT‐PCR reaction was performed using SYBR and 0.5 pmol/μl of forward and reverse primers in a Light cycler 480 instrument (Roche Diagnostic).

Primers used are summarized in Expanded View Table EV5.

### Proteasome activity assay using activity‐based probes (ABPs)

The activity of all catalytic subunits of the proteasome was assayed in native cell lysates using ABP kindly provided by Bobby Florea and Hermen Overkleeft (de Bruin *et al*, 2016). Protein extracts were first diluted to a protein concentration to 0.5 μg/μl using native lysis buffer without digitonin. 15 μg of protein was then incubated with 0.5 μM MV151 (pan‐ABP), 0.25 μM LW124 (β1/LMP2 specific ABP), or 1 μM MVB127 (β5/LMP7 specific ABP), respectively, by incubation on a shaker at 37°C for 1 h. Protein samples were then mixed with 6× Laemmli buffer and separated on 15% Tris‐glycine SDS polyacrylamide gels for 2.5 h. Catalytically active proteasome subunits were detected upon binding of the fluorescently labeled ABPs to the active site using a fluorescent scanner (Typhoon TRIO^+^, Amersham Biosciences). Images were taken with the fluorescence filters Cy3/TAMRA for ABPs MV151 and MVB127 and the Cy2 fluorescent channel for LW124. Equal protein loading of the gels was confirmed by PageBlue™ staining (Thermo Fisher Scientific).

### Relative quantification of cytosolic mtDNA

1 × 10^6^ MEFs cells were plated in 10‐cm dishes. After 48 h, cells were counted, centrifuged at 18,531 *g* for 15 min at 4°C. The supernatant was used for DNA purification with the DNeasy Blood & Tissue Kit according to the manufacturer's protocol (Qiagen). Quantitative PCR was employed to measure cytosolic mitochondrial DNA with Nd2 and Nd4 primers. 18S rDNA was used as a housekeeping gene. DNA isolated from MEF cells treated with 10 μM thapsigargin (Sigma, T9033) for 6 h was used as a positive control. Primers used are included in Table EV5.

### Absolute quantification of cytosolic mtDNA

5 × 10^6^ MEFs cells were collected from a single *PolgA* ^*+/+*^ and *PolgA* ^mut/mut^ line at three different passages. Cells were lysed in 200 μl Digitonin lysis buffer (50 mM HEPES, pH 7.4; 150 mM NaCl; 18 μg/ml digitonin, protease inhibitors) by pipetting gently up and down and incubation on an end‐over‐end tube rotator for 10 min at 4°. After centrifugation at 950 *g* for 5 min at 4°C, the supernatant was transferred to a new 1.5‐ml tube. Genomic DNA was precipitated from the cytosolic fraction of the cells using the *Wizard^®^ Genomic DNA Purification Quick Kit* (Promega). DNA was further purified using the *NucleoSpin^®^ Gel and PCR‐Clean‐up Kit* from Takara Bio USA Inc according to the manufacturer's instructions. The absolute mtDNA copy number was determined using the *Absolute Human Mitochondrial DNA Copy Number Quantification qPCR Assay Kit* (AHMQ, Catalog #8948).

### Proteomic analysis

Proteomic analysis of *PolgA* ^*+/+*^ and *PolgA* ^mut/mut^ cells was performed as detailed previously in Meul *et al* (2020). The entire proteomic dataset has been deposited in the ProteomeXchange Consortium at https://proteomecentral.proteomexchange.org/cgi/GetDataset using the dataset identifier PXD019695.

### Flow cytometry analysis

*PolgA* ^*+/+*^ and *PolgA* ^mut/mut^ cells were seeded with 30,000 cells in 12‐well culture plates for 48 h, trypsinized and collected in flow cytometry tubes (Corning), washed with FACS buffer (2% FBS in PBS buffer) and stained with PE‐labeled anti‐mouse H‐2K^b^ antibody (BioLegend, #116507) in a 1:100 dilution or PE‐labeled anti‐mouse IgG2a, κ isotype control antibody (BioLegend, #400211) for 30 min at 4°C. After further washing of the samples with FACS buffer once, fluorescent signals were detected by flow cytometry analysis using the Becton Dickinson BD™ LSR II Flow Cytometer System (BD Biosciences‐Europe) and the FlowJo software (TreeStart Inc; Ashland, USA, version 7.6.5) for analysis. For treatment, *PolgA* ^mut/mut^ cells were seeded into 12‐well culture plates and 24 h after seeding treated with 200 nM immunoproteasome inhibitor Lu005i kindly provided by Bobby Florea and Hermen Overkleeft (de Bruin *et al*, 2014) for 24 h or treated with 75 IU/ml mouse recombinant IFNγ (Roche, 11276905001) for 24 h. Cells were then collected as described above and stained with PE‐labeled anti‐mouse H‐2K^b^ antibody for detection by flow cytometry. *PolgA* ^mut/mut^ cells were seeded into 12‐well culture plates and transfected with 5 nM cGAS siRNA or non‐sense scrambled control (NC) siRNA for 48 h. H‐2K^b^ surface expression was then determined by flow cytometry as described above.

phLF cells (150,000 cells) were seeded into 12‐well culture plates and transfected with mtDNA, DNAse I‐pretreated mtDNA, DNase I for 48 h or with cGAMP for 24 h. Alternatively, phLF cells seeded in 12‐well culture plates were treated with 75 IU/ml IFNγ (Roche, 11040596001) or 100 IU/ml IFNβ (abcam, ab71475) for 24 h. After trypsinization, cells were washed with FACS buffer and stained cells with FITC‐labeled antihuman HLA‐A2 antibody (Clone BB7.2, BioLegend, #343304) in a 1:50 dilution or FITC‐labeled anti‐mouse IgG2b, κ Isotype ctrl antibody (BioLegend, #401205) for 30 min at 4°C. Samples were washed again with FACS buffer and then analyzed as described above. For detection of the influenza M1‐HLA‐A2 restricted epitope (GILGFVFTL), phLF cells were seeded into 12‐well culture plates and transfected with 100 ng/ml human mtDNA, mtDNA digested with DNase I for 48 h or with cGAMP for 24 h. Alternatively, cells were treated with 100 IU/ml IFNβ for 24 h. Cells were then transfected with 1 μg/ml influenza M1 protein (SinoBiological, #40010‐V07E) dissolved in MEM using the CRISPRMAX reagent (Thermo Fisher Scientific, CMAX00003) for 4.5 h. Cells were then trypsinized and stained with A647 Influenza M1/HLA‐A2 Complex Antibody (405H1.01, Novus Biologicals, DDX0270A647) in a 1:100 dilution for 30 min at 4°C and analyzed as described above. phLF treated with 75 IU/ml IFNγ for 24 h and incubated in the presence of 1 μg/ml CEF1, Influenza Matrix Protein M1 (58–66; GILGFVFTL, GenScript Biotech, RP19978) for 90 min served as a positive control.

### Immunofluorescence analysis of cells

Mouse embryonic fibroblasts cells were seeded onto cover slides in 24‐well culture plates for 48 h. Cells were then fixed with 4% PFA for 10 min and permeabilized by 0.1% Triton‐X 100 in PBS for 10 min. Upon washing, cells were blocked in 5% BSA for 1 h and the primary anti‐STAT1 antibody (Cell Signaling Technology, #9172) was added at 4°C overnight at a dilution of 1:200 in antibody diluent (Dako, S0809). Cover slides were then washed with 0.05% Tween‐20 in PBS for 15 min and Alexa Fluor 488‐labeled goat anti‐rabbit IgG (Thermo Fisher Scientific, A11008) was added at a dilution of 1:250 together with DAPI (Sigma‐Aldrich, 28718‐90‐3) at a dilution of 1:2,000 for 1 h. Slides were again washed three times with 0.05% Tween‐20 in PBS for 15 min each and then mounted onto glass with fluorescent mounting medium (DAKO, S3023). Confocal microscopy was performed using the TCS SP8 microscope (Leica) and Leica Application Suite X core (version 3.7.4, Leica).

*PolgA* ^*+/+*^ and *PolgA* ^mut/mut^ cells were seeded onto cover slides in 12‐well culture plates for 24 h. Cells were then fixed with pre‐iced methanol for 15 min and permeabilized by acetone for 15 min in an ice bath. Upon washing, cells were blocked in Roti®Block buffer (Carl‐Roth, A151.1) for 1 h at room temperature and the primary anti‐HSP60 antibody (1:100, Cell Signaling Technology, #4870) and anti‐DNA antibody (1:200, Millipore, CBL186) were added at 4°C overnight in Roti®Block buffer. Cover slides were then washed with 0.05% Tween‐20 in PBS for 15 min, and Alexa Fluor 555‐labeled goat anti‐mouse IgG (Thermo Fisher Scientific, A21422) and Alexa Fluor 488‐labeled goat anti‐rabbit IgG H&L (Abcam, ab150077) were added at a dilution of 1:2,000 for 1 h. Slides were again washed three times with 0.05% Tween‐20 in PBS for 15 min each and then mounted onto glass slides with fluorescent mounting medium containing DAPI (Carl‐Roth, HP20.1). Confocal microscopy was determined using TCS SP5 microscope (Leica) and Leica Application Suite AF (version 2.7.3, Leica). Fluorescent intensity of single cell was calculated using ImageJ (version 1.53t).

### Patients and control group

In total, we included 91 patients in the analysis, divided into patients with IPF (*n* = 69), and healthy controls (*n* = 21). The study was performed in accordance with protocols approved by the Ludwig‐Maximilians‐Universität München Ethic's Review Board (Ethic commission numbers 180‐14 and 454‐12) as described (Fernandez *et al*, 2016). All subjects provided informed written consent for the research study. Healthy volunteers were without signs of current infection, inflammation, or respiratory symptoms at the time of blood sampling. Diagnosis of IPF was performed by multidisciplinary consensus, based on current international criteria (Raghu *et al*, 2015).

### Cell isolation and flow cytometric analysis

For immunophenotyping, fresh venous blood was collected in EDTA‐coated vacutainer tubes (Sarstedt; Nümbrecht, Germany). Briefly, PBMC were used for flow cytometric detection of lymphocyte subtypes. Lymphocyte subtypes were determined as T cells (CD3^+^), T cytotoxic (CD3^+^CD8^+^): effector (CCR7^−^) and noneffector (CCR7^+^), as previously reported (Fernandez *et al*, 2016). For lymphocyte analysis, PBMC were prepared from blood samples by density gradient sedimentation (LymphoprepTM STEMCELL Technologies). Trypan blue staining was used for differentiation between viable and nonviable cells and showed viability of > 90% for all cells used in this study. After gradient separation, cells were stained with lymphocyte antibody mix for 20 min at 4°C in the dark. Data acquisition was performed in a BD LSRII flow cytometer. Data were analyzed with the FlowJo software. Negative thresholds for gating were set according to isotype‐labeled controls.

### Immunofluorescence analysis of human and mouse lung tissue

For immunofluorescence, tumor‐free areas (control) or explanted lungs of IPF patients were analyzed. All participants gave written informed consent, and the study was approved by the local ethics committee (19‐630). Immunofluorescence staining was performed in lung tissue embedded in paraffin. For that, slides were deparaffinized and rehydrated by incubating overnight at 60°C, following by rehydration where slides were twice immersed in xylene (5 min, each), transferred to 100% ethanol (2 min, each), once in 90% ethanol (1 min), 80% ethanol (1 min), 70% ethanol (1 min), and finally flushed with distillated water (30 s). Next, for antigen retrieval step, we immerged the slides in citrate buffer solution (pH 6.0) and placed them into a Decloaking Chamber at 125°C for 30 s, and at 90°C for 10 s before cooling them down to room temperature. Slides were then washed three times in Tris buffer, followed by blocking in 5% BSA—Tris buffer solution (40 min) to prevent nonspecific binding. Furthermore, the slides were double stained with anti‐human CD8a (372902, Biolegend) and anti‐PD1 (ab137132, Abcam) or anti‐LMP2 (ab3328, Abcam). The primary antibodies were diluted using the antibody diluent (Zytomed Systems, ZUC025‐100): anti‐CD8a (1:100), anti‐PD1 (1:250), and anti‐LMP2 (1:200). Antibody‐matching IgGs (isotype) were used for control staining. The slides were placed in a wet chamber followed by the addition of the primary antibodies and incubated at 4°C overnight. Slides were rinsed three times with Tris buffer and a 1:250 dilution of the respective secondary antibody (A‐21202 Alexa Fluor 488 donkey anti‐mouse and A10042 Alexa Fluor 568 donkey anti‐rabbit; Invitrogen), together with DAPI (D9564, Sigma‐Aldrich; 1:2,000) was applied and incubated at room temperature for 1 h in the darkness. Once again, the slides were rinsed three times with Tris buffer, and covered with Fluorescence Mounting Medium (S3023, Dako). Images were obtained using the Axiovert II (Carl Zeiss Microscopy GmbH) and processed using the Zen 2.6 (blue edition) software (Carl Zeiss Microscopy GmbH).

### Bioinformatic analysis

Publicly available single‐cell RNA‐seq dataset (GSE135893) was downloaded from the NCBI Gene Expression Omnibus (GEO) database and was analyzed using Seurat, an R package for single‐cell analysis (http://satijalab.org/seurat/). Cells with more than 8,000 or fewer than 1,000 detected genes, as well as cells expressing more than 25% mitochondrial genes, were excluded. The gene expression heatmap plot was drawn using the pheatmap function of pheatmap (version 1.0.12) package in R. Gene set enrichment analysis of single cell types were performed with the R package escape (version 1.0.0). Signatures for CD8^+^ T‐cell activation were manually curated and provided in Table EV1. Gene signatures for T‐cell exhaustion were obtained from Kusnadi *et al* (2021) and are provided in Table EV2. Gene annotation enrichment analysis was performed with the 1D annotation enrichment algorithm as detailed previously (Meul *et al*, 2020) and progenesis output tables were drawn using ggplot2 (version 3.3.5) package in R.

### CD8^+^ UTY T‐cell activation assay

Mouse embryonic fibroblasts cells or splenocytes were seeded in round or flat bottom 96‐well culture plates for 48 h, respectively. A separate well was used for counting the cells using trypan blue exclusion assay (Thermo Fisher Scientific, 15250061) in order to determine the exact cell number for T‐cell co‐culturing. MEFs were co‐cultured with CD8^+^ UTY reporter T cells in ratios of 1:0.5, 1:1, 1:2, 1:4. Splenocytes were co‐cultured in a ratio of 1:2. After 24 h, the 96‐well culture plates were then centrifuged for 10 min at 2,138 *g*, medium was removed and beta galactosidase activity of the UTY‐reporter T cells was assayed by adding 200 μl of chlorophenol red‐β‐D‐galactopyranoside (CPRG) buffer (0.45% β‐mercaptoethanol, 4.5 mM MgCl_2_, 0.13% NP40, 78.6 mM CPRG) at 37°C. Substrate turnover was quantified after 18 h by measuring absorbance at 560 and 620 nm using a Sunrise plate reader (Tecan, Switzerland). The background activity of UTY‐reporter T cells alone was subtracted from all values.

### CD8^+^ Influenza‐M1 T‐cell activation assay

phLF were plated in 12‐well culture plates and treated with 100 IU/ml IFNβ or 75 IU/ml IFNγ for 24 h. 1 μg/ml M1 peptide (Genscript, RP19978) was added into the medium for 90 min and cells then washed with PBS. A separate well was used for counting the cells using trypan blue exclusion assay (Thermo Fisher Scientific, 15250061) in order to determine the exact cell number for T‐cell co‐culturing. phLF cells were co‐cultured with the CD8^+^ Influenza‐M1‐specific T cells (4VA1 CD8^+^ T cells) at the ratio of 1:5 for 24 h. The supernatant was then harvested and centrifuged at 2,138 *g* for 5 min. IL‐2 secreted by the mouse‐derived T‐cell clone was detected in the supernatant using the mouse IL‐2 MAX™ standard set (Biolegend, 431001) and DuoSet® Ancillary Reagent Kit 2 (R&D systems, #DY008). Secretion of IL‐2 per CD8^+^ T cells was calculated by multiplying the measured concentration of IL‐2 with the volume of supernatant of 4VA1 CD8^+^ T cell and phLF co‐cultures in ml divided by CD8^+^ T‐cell numbers.

### IFNβ ELISA

*PolgA* ^*+/+*^ and *PolgA* ^mut/mut^ cells were seeded in 12‐well plates and cultured for 48 h in 1 ml DMEM High glucose complete medium. Cell culture supernatants were harvested and centrifuged at 2,138 *g* for 5 min. *PolgA* ^*+/+*^ cells were transfected for 48 h either with 100 ng/ml mouse mtDNA or mtDNA digested with 20 μg/ml DNase I for 30 min at 37°C. Alternatively, cells were transfected with 4 μg/ml cGAMP for 24 h. Cell culture supernatants were then harvested and centrifuged at 2,138 *g* for 5 min. Supernatants were analyzed for IFNβ secretion using LEGEND MAX™ Mouse IFN‐β ELISA Kit according to the manufacturer's recommendations (Biolegend, #439407).

### Immunohistochemistry staining of LMP2

Human lung sections embedded in paraffin were deparaffinized using a standard protocol as described above (Keller *et al*, 2015). Endogenous peroxidase activity was quenched by incubating the slides in 80% methanol and 1.8% H_2_O_2_ for 20 min. Heat‐induced antigen retrieval was performed in 0.05% citraconic anhydride buffer (pH 7.4). Slides were washed with TBST buffer (20 mM Tris, 135 mM NaCl, 0.02% Tween, pH 7.6) for 15 min, blocked with Rodent Block M (Biocare, USA) for 30 min, washed and incubated for 60 min with LMP2 antibody (1:600, Abcam, ab3328). After another washing step, slides were incubated with rabbit‐polymer coupled to alkaline phosphatase (Biocare) for 30 min, washed again, and incubated with the substrate Vulcan Fast Red (Biocare) for 12 min. Hematoxylin counterstaining was performed, and slides were dehydrated and mounted in Eukitt® (Sigma‐Aldrich). Slides were documented using a MIRAX scanning system (Zeiss).

### Statistical analysis

All statistical analysis was performed using GraphPad Prism (Version 9.00). Data were reported in the figures as mean ± SEM. Comparison between two groups was carried out using Student's unpaired *t*‐test. Comparisons of more than three groups was carried out using one‐way ANOVA test. Statistical significance was determined as *P < 0.5, **P < 0.01, ***P < 0.001. Detailed statistical information for each individual dataset is provided in the figure legends.

## Author contributions

**Xinyuan Wang:** Conceptualization; formal analysis; visualization; methodology; writing – review and editing. **Huabin Zhang:** Data curation. **Yuqin Wang:** Formal analysis; methodology. **Laylan Bramasole:** Formal analysis; methodology; writing – review and editing. **Kai Guo:** Formal analysis; methodology. **Fatima Mourtada:** Formal analysis; methodology. **Thomas Meul:** Formal analysis; investigation. **Qianjiang Hu:** Formal analysis. **Valeria Viteri:** Data curation; formal analysis. **Ilona Kammerl:** Supervision; methodology. **Melanie Koenigshoff:** Supervision. **Mareike Lehmann:** Supervision; methodology. **Thomas Magg:** Resources. **Fabian Hauck:** Resources. **Isis E Fernandez:** Formal analysis; supervision; visualization. **Silke Meiners:** Conceptualization; supervision; writing – original draft; writing – review and editing.

## Disclosure and competing interests statement

The authors declare that they have no conflict of interest.

## Supporting information



Expanded View Figures PDF

Table EV1

Table EV2

Table EV3

Table EV4

Table EV5

PDF+

Source Data for Figure 1

Source Data for Figure 2

Source Data for Figure 3

Source Data for Figure 4

Source Data for Figure 5

Source Data for Figure 6

Source Data for Figure 7

Source Data for Figure 8

Source Data for Figure 9

## Data Availability

All data and materials are available upon request. The proteomics data are deposited under the following link http://proteomecentral.proteomexchange.org/cgi/GetDataset?ID=PXD019695.
