# Supplementary material for: DNA sensing via the cGAS/STING pathway activates the immunoproteasome and adaptive T‐cell immunity
Source: EMBO J. 2023 Mar 13;42(8):e110597. doi: 10.15252/embj.2022110597 (PMC10106989; doi:10.15252/embj.2022110597)
Supplement: Supplementary file 1 — Expanded View Figures PDF [file EMBJ-42-e110597-s016.pdf]

Expanded View Figures

Figure EV1. Mitochondrial DNA stress in *PolgA*$^{mut/mut}$ cells.

A Abundance of cytoplasmic mtDNA genes *Nd2* and *Nd4* in three *PolgA*$^{+/+}$ and three *PolgA*$^{mut/mut}$ cell lines as biological replicates. Values are given as fold over *PolgA*$^{+/+}$ control with the mean of normalized *PolgA*$^{+/+}$ signal set to 1. Single data points are provided to denote the individual cell lines.

B Cytoplasmic mtDNA genes *Nd2* in *PolgA*$^{+/+}$ cells treated with 10 μM thapsigargin for 6 h serving as a positive control for the detection of cytosolic mtDNA. Three independent experiments were performed.

C Detection of cytoplasmic DNA in *PolgA*$^{+/+}$ and *PolgA*$^{mut/mut}$ cells by using confocal microscope. Cells were stained with DAPI (blue), anti-HSP60 (green) and anti-DNA (red). Arrows indicate red dsDNA signals outside the nucleus. Scale bar on the figures represents 50 μm. Quantification of cytoplasmic DNA was from independent fields ($n \geq 6$) with 62 *PolgA*$^{+/+}$ and 55 *PolgA*$^{mut/mut}$ cells counted.

D Densitometric analysis of cGAS, STING, pTBK1, TBK1, pIRF3, IRF3, pIκBα, IκBα, pP65, and P65 expression normalized to β-actin loading control in *PolgA*$^{+/+}$ and *PolgA*$^{mut/mut}$ cells from three biological replicates as exemplarily shown in Western blot in Fig 2D.

Data information: Data are shown as mean ± SEM and were analyzed with two-tailed unpaired Student's *t*-test. Asterisks indicate significance as *$P < 0.05$, **$P < 0.01$, ***$P < 0.001$.

© 2023 The Authors

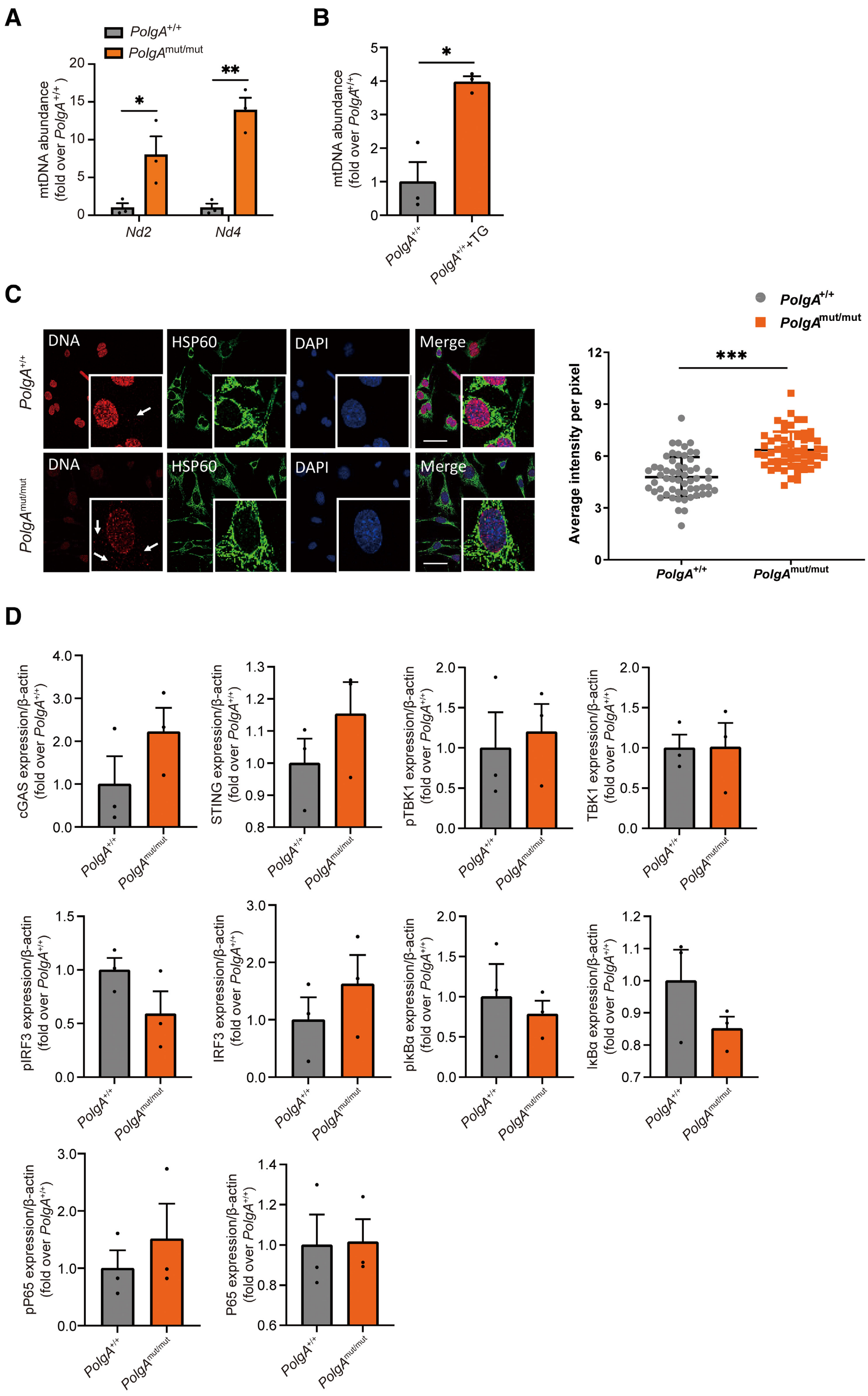

Figure EV1.

© 2023 The Authors

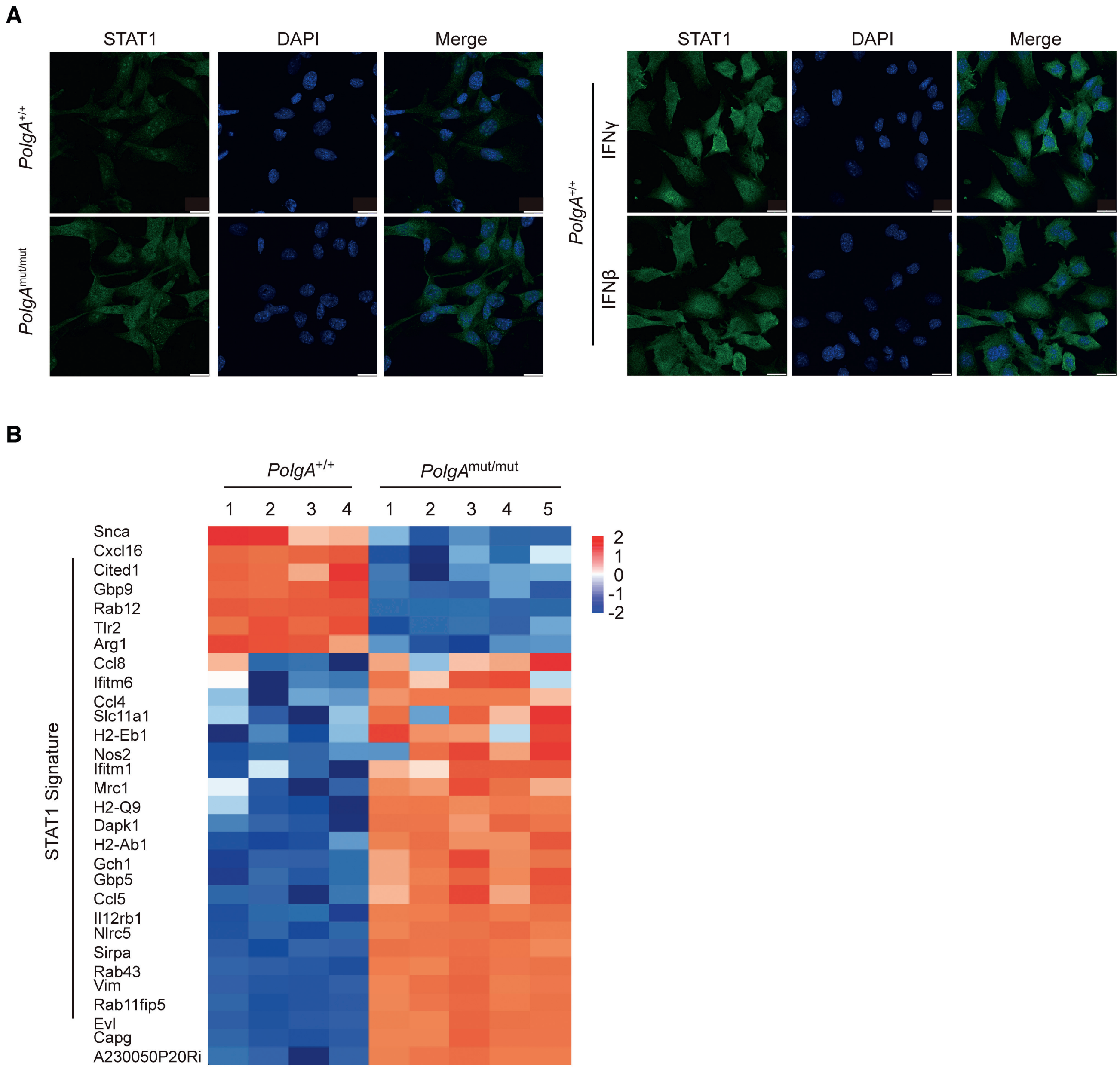

Figure EV2. Activation of STAT1 in *PolgA*$^{mut/mut}$ cells.

A Immunofluorescence analysis of STAT1 in *PolgA*$^{+/+}$ and *PolgA*$^{mut/mut}$ cells, as well as in *PolgA*$^{+/+}$ cells treated with 100 IU/ml IFNβ or 75 IU/ml IFNγ for 24 h, scale bar: 20 μm.

B Heatmap of mRNA expression values of significantly regulated STAT1-stimulated genes obtained from RNA seq analysis of *PolgA*$^{+/+}$ (four independent replicates of the same cell line) and *PolgA*$^{mut/mut}$ (five independent replicates of the same cell line) cells.

© 2023 The Authors

Figure EV3. cGAS/STING-dependent induction of the immunoproteasome upon mitochondrial DNA stress.

A Representative Western blot and densitometric analysis for LMP2, LMP7, STAT1 and β-actin (loading control) in extracts of *PolgA*$^{+/+}$ cells treated with 100 IU/ml IFNβ for 48 h in three independent biological experiments.

B Representative Western blot for cGAS and STING in *PolgA*$^{mut/mut}$ cells upon siRNA transfection of non-sense scrambled control (NC), cGAS or STING-specific siRNAs. Untransfected cells were used as controls (Ctrl). β-actin served as a loading control.

C Western blot for LMP2 and LMP7 in *PolgA*$^{+/+}$ cells pretreated with 0.04 μg/ml, 0.1 μg/ml, 0.2 μg/ml or 0.5 μg/ml IFNAR1 Ab for 24 h and treated with IFNβ, IFNγ or transfected with HT-DNA for 48 h.

D RT-qPCR analysis of TFAM in wild-type (WT) and cGAS KO MEFs upon silencing of TFAM with siRNA from three independent biological experiments.

E Representative Western blot for cGAS in wild-type (WT) and cGAS KO MEFs, β-actin served as a loading control.

F ELISA of secreted IFNβ in supernatants from *PolgA*$^{+/+}$ cells transfected with cGAMP, mtDNA, DNase I pretreated mtDNA and DNase I (n = 3 independent biological experiments).

G Representative Western blot for STING in wild-type (WT), cGAS KO MEFs and STING$^{gt/gt}$ loss-of-function MEFs, β-actin served as a loading control.

Data information: All data are shown as mean ± SEM and were analyzed with two-tailed unpaired Student's *t*-test. Asterisks indicate significance as *P < 0.05, **P < 0.01.

© 2023 The Authors

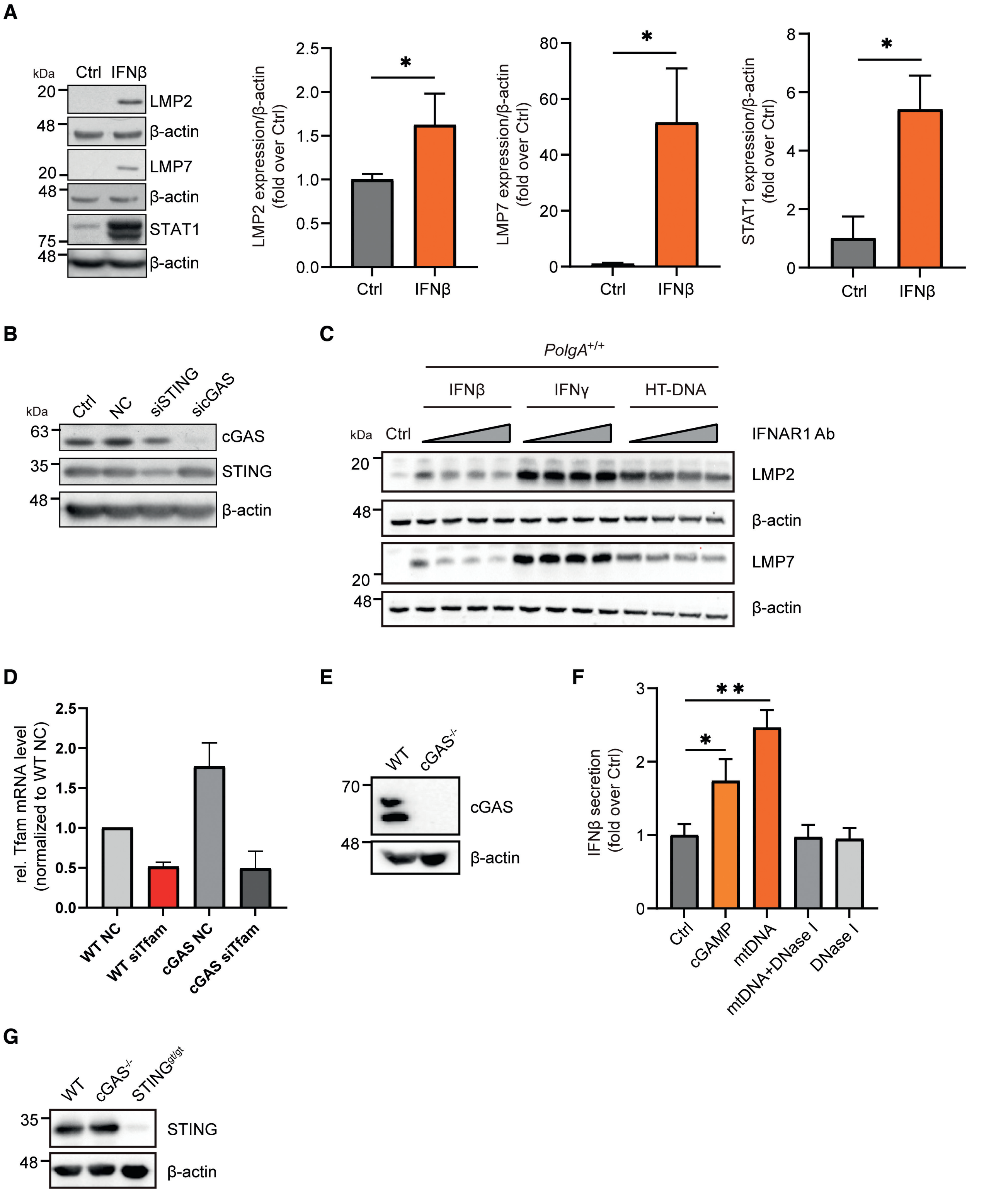
A
kDa Ctrl IFNβ
20 LMP2
48 β-actin
LMP7
20
48 β-actin
STAT1
75
48 β-actin
LMP2 expression/β-actin (fold over Ctrl)
LMP7 expression/β-actin (fold over Ctrl)
STAT1 expression/β-actin (fold over Ctrl)
Ctrl IFNβ
B
kDa Ctrl NC siSTING sicGAS
63 cGAS
35 STING
48 β-actin
C
PolgA+/+
IFNβ IFNγ HT-DNA
kDa Ctrl IFNAR1 Ab
20 LMP2
48 β-actin
LMP7
20
48 β-actin
D
rel. Tfam mRNA level (normalized to WT NC)
WT NC WT siTfam cGAS NC cGAS siTfam
E
WT cGAS-/-
70 cGAS
48 β-actin
F
IFNβ secretion (fold over Ctrl)
Ctrl cGAMP mtDNA mtDNA+DNase I DNase I
G
WT cGAS-/- STINGgt/gt
35 STING
48 β-actin

Figure EV3.

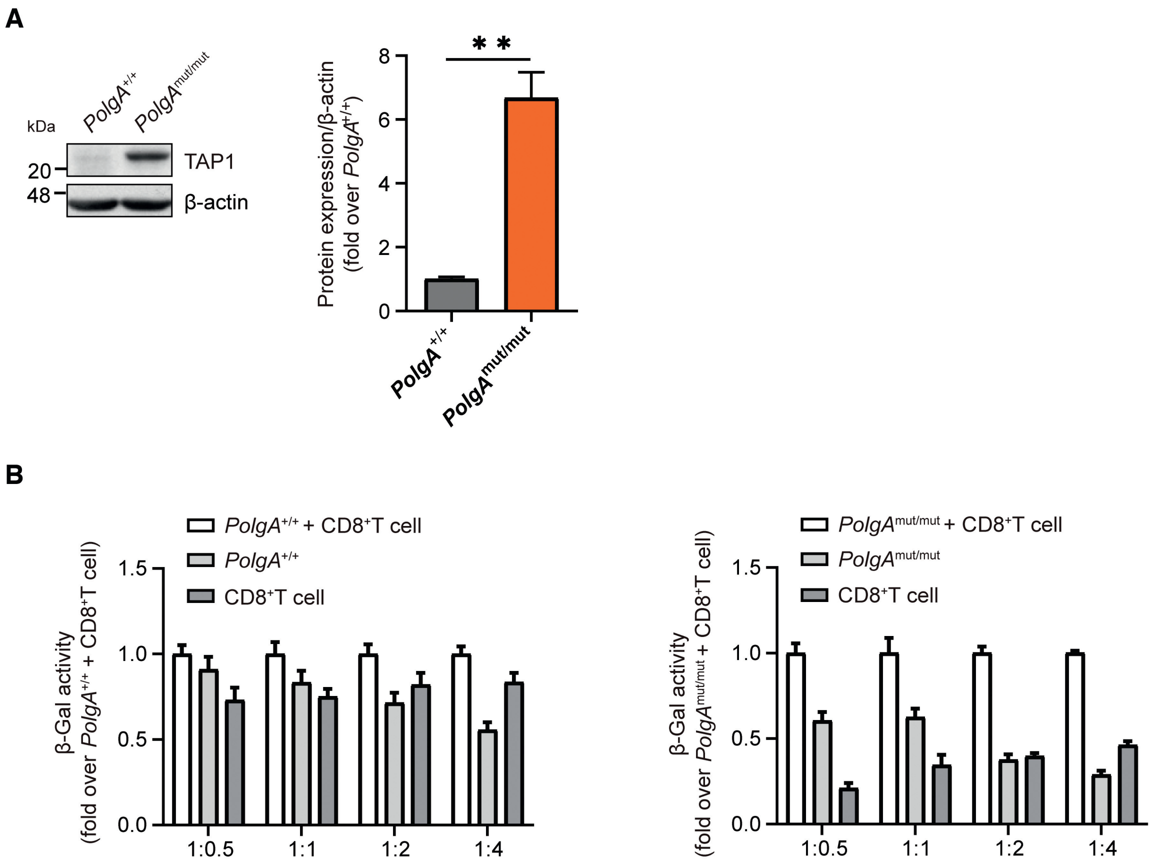

Figure EV4. TAP1 expression and $CD8^+$ T-cell reporter activation assay.

A Western blot analysis of TAP1 and β-actin (loading control) and densitometric analysis from three *PolgA*$^{+/+}$ and three *PolgA*$^{mut/mut}$ cell lines. Densitometric analysis shows mean ± SEM of three biological replicates and is presented as fold over mean of *PolgA*$^{+/+}$ set to 1.

B $CD8^+$ reporter T cell background in co-culture with *PolgA*$^{+/+}$ or *PolgA*$^{mut/mut}$ cells. Data are provided as normalized values to the β-galactosidase (β-Gal) activity of co-cultured MEFs and $CD8^+$ T reporter cells. Data show mean ± SEM of three independent biological experiments.

Data information: All data were analyzed with two-tailed unpaired Student's *t*-test. Asterisks indicate significance as $^{**}P < 0.01$.

 © 2023 The Authors

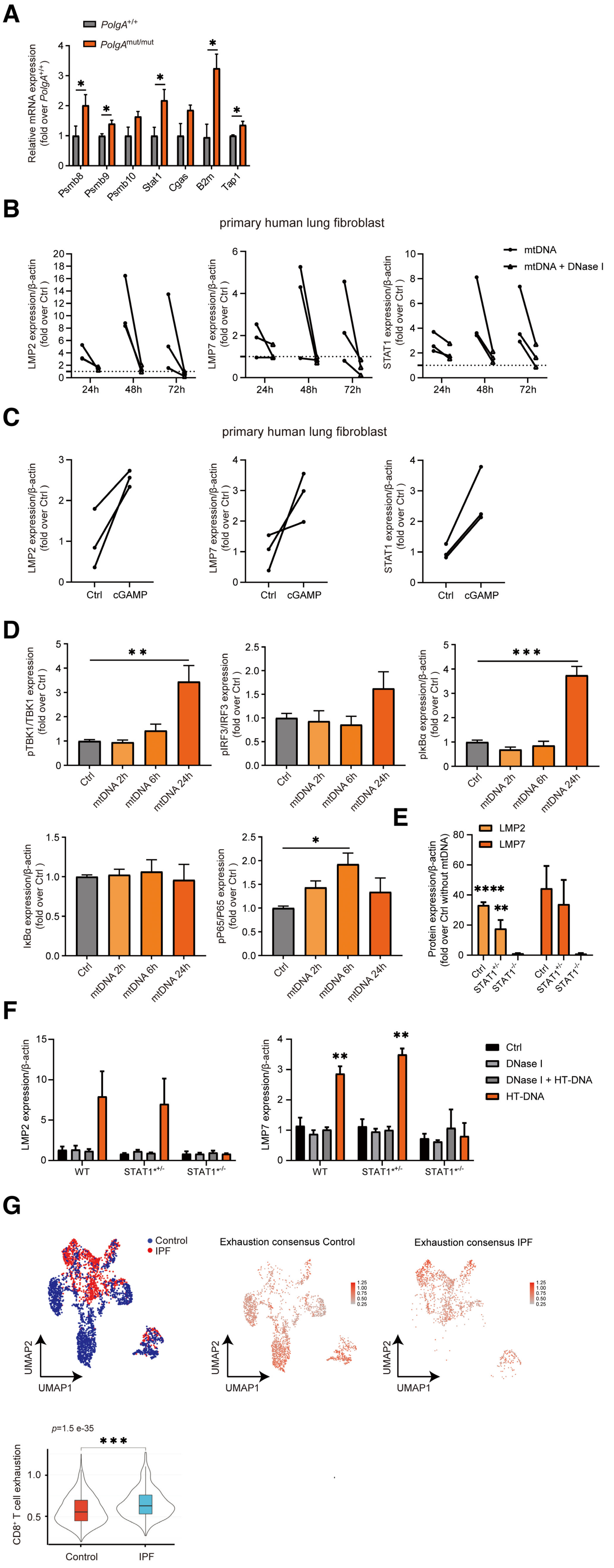

Figure EV5. Induction of the immunoproteasome in lung cells upon mtDNA stress.

A RT-qPCR analysis of immunoproteasome subunits *Psmb8*, *Psmb9*, *Psmb10*, and of *Stat1*, *Cgas*, *B2m* and *Tap1* mRNA expression in *PolgA*$^{+/+}$ and *PolgA*$^{mut/mut}$ mice lungs (3–4 *PolgA*$^{+/+}$ and *PolgA*$^{mut/mut}$ mice).

B Densitometric analysis of LMP2, LMP7, and STAT1 expression normalized to β-actin loading control in phLFs as exemplarily shown in the Western blot in Fig 6C using three different phLF cell lines isolated each from different donors. Values are given as fold over control with the respective control signal set to 1 and indicated by the dashed line.

C Densitometric analysis of LMP2, LMP7, and STAT1 expression normalized to β-actin loading control in phLFs as exemplarily shown in Western blot in Fig 6D. Values are given as fold over control normalized to the mean of the controls.

D Densitometric analysis of Western blots for STING, pTBK1, TBK1, pIRF3, IRF3, pIκBα, IκBα, pP65, P65 and β-actin (loading control) in phLFs transfected with mtDNA for 2 h, 6 h, or 24 h, respectively as exemplarily shown in Western blot in Fig 6G. Densitometric analysis shows mean ± SEM of three independent experiments using one phLF line and are presented as fold over mean of control.

E Densitometric analysis of Western blots (Fig 6I) for LMP2 and LMP7 in neonatal wild-type (WT), adult heterozygous STAT1 mutant (STAT1*$^{+/-}$) and neonatal homozygous STAT1 mutant (STAT1*$^{-/-}$) skin fibroblasts transfected with mtDNA. Fold over Ctrl without transfection is shown, respectively ($n = 3$ independent biological experiments).

F Densitometric analysis of Western blots (Fig 8C) for LMP2 and LMP7 in skin fibroblast transfected with HT-DNA or HT-DNA pretreated with DNase I, respectively ($n = 3$ independent biological experiments).

G UMAP and violin plot analysis of scRNA seq T-cell exhaustion gene signature in control and IPF CD3^{+}CD8^{+} T cells using the publicly available data set GSE135893. scRNA seq data from 24 IPF patients and 10 donors as controls were analyzed with two-tailed unpaired Student's *t*-test. The violin plots represent T cell exhaustion consensus enrichment score density and distribution. Elements in boxplot: Upper and lower whisker = max and least value excluding outliers, box edges the first and third quartiles, boxplot midline represents the median.

Data information: All data are shown as mean ± SEM and were analyzed with two-tailed unpaired Student's *t*-test. Asterisks indicate significance as *$P < 0.05$, **$P < 0.01$, ***$P < 0.001$, ****$P < 0.0001$.

© 2023 The Authors
