# Supplementary material for: DNA sensing via the cGAS/STING pathway activates the immunoproteasome and adaptive T‐cell immunity
Source: EMBO J. 2023 Mar 13;42(8):e110597. doi: 10.15252/embj.2022110597 (PMC10106989; doi:10.15252/embj.2022110597)
Supplement: Supplementary file 2 — Table EV1 [file EMBJ-42-e110597-s012.docx]

**Table EV1. List of enriched pathways as shown in Figure 2A**

| **Column** | **Type** | **Name** | **Score** | **P value** | **Benj. Hoch. FDR** | **Mean** | **Median** |
| --- | --- | --- | --- | --- | --- | --- | --- |
| *PolgA*^mut/mut^ */ PolgA*^+/+^ | GOBP name | RNA methylation | -0.9371 | 0.00496 | 0.10146 | -0.860 | -0.886 |
| *PolgA*^mut/mut^ */ PolgA*^+/+^ | GOBP name | mRNA splice site selection | -0.8782 | 0.00067 | 0.01997 | -0.835 | -0.626 |
| *PolgA*^mut/mut^ */ PolgA*^+/+^ | GOMF name | cytochrome-c oxidase activity | -0.859 | 0.00027 | 0.01315 | -1.218 | -1.285 |
| *PolgA*^mut/mut^ */ PolgA*^+/+^ | GOMF name | heme-copper terminal oxidase activity | -0.859 | 0.00027 | 0.01280 | -1.218 | -1.285 |
| *PolgA*^mut/mut^ */ PolgA*^+/+^ | GOMF name | oxidoreductase activity, acting on a heme group of donors, | -0.859 | 0.00027 | 0.01247 | -1.218 | -1.285 |
| *PolgA*^mut/mut^ */ PolgA*^+/+^ | GOBP name | renal tubule development | 0.9271 | 0.00544 | 0.10845 | 0.9962 | 0.9506 |
| *PolgA*^mut/mut^ */ PolgA*^+/+^ | GOBP name | antigen processing and presentation of exogenous peptide antigen | 0.9295 | 0.00532 | 0.10676 | 0.9249 | 0.9360 |
| *PolgA*^mut/mut^ */ PolgA*^+/+^ | GOMF name | 2'-5'-oligoadenylate synthetase activity | 0.9367 | 0.00498 | 0.09499 | 1.1103 | 1.2350 |
| *PolgA*^mut/mut^ */ PolgA*^+/+^ | GOCC name | TAP complex | 0.9454 | 0.00459 | 0.03697 | 1.1291 | 1.0477 |
| *PolgA*^mut/mut^ */ PolgA*^+/+^ | GOBP name | response to type I interferon | 0.9815 | 0.0032 | 0.07298 | 1.6088 | 1.6793 |
