# Supplementary material for: DNA sensing via the cGAS/STING pathway activates the immunoproteasome and adaptive T‐cell immunity
Source: EMBO J. 2023 Mar 13;42(8):e110597. doi: 10.15252/embj.2022110597 (PMC10106989; doi:10.15252/embj.2022110597)
Supplement: Supplementary file 3 — Table EV2 [file EMBJ-42-e110597-s014.docx]

**Table EV2. List of genes involved in CD8^+^ T cell activation (used for Figure 9B)**

| CD8a |
| --- |
| CD3e |
| CD3g |
| CD3d |
| LCK |
| NKG7 |
| PRF1 |
| GZMB |
| GZMA |
| GNLY |
| CXCR3 |
| CD69 |
| NKG2D |
| CD5 |
| ICOS |
| KLRG1 |
| PDCD1 |
| HAVCR2 |
| BTLA |
| SLAMF6 |
| DGKA |
| TBX21 |
| EOMES |
| SIT1 |
| TCF1/7 |
| TOX |
| NFATC1 |
| LAT |
| ZAP70 |
| RASGRP1 |
| CBLB |
| IL1B |
| IL15 |
