# Supplementary material for: DNA sensing via the cGAS/STING pathway activates the immunoproteasome and adaptive T‐cell immunity
Source: EMBO J. 2023 Mar 13;42(8):e110597. doi: 10.15252/embj.2022110597 (PMC10106989; doi:10.15252/embj.2022110597)
Supplement: Supplementary file 4 — Table EV3 [file EMBJ-42-e110597-s007.docx]

**Table EV3. List of genes involved in CD8^+^ T cell exhaustion (used for Figure EV5G)**

| TOX |
| --- |
| HAVCR2 |
| LAG3 |
| ENTPD1 |
| PDCD1 |
| CTLA4 |
| CD38 |
| TIGIT |
| VCAM1 |
| CD27 |
| SNAP47 |
| IGFLR1 |
| RAD51 |
| CCNB1 |
| BUB1 |
| SIRPG |
| SEMA4A |
| CXCR6 |
| FUT8 |
| HLA-DMA |
| ITGAE |
| UBE2F |
| NDFIP2 |
| CD63 |
| FKBP1A |
| TPI1 |
| CDCA8 |
| NCAPG2 |
| CDKN3 |
| CCL4L2 |
| RGS2 |
| NAB1 |
| ID3 |
| CCR5 |
| GOLIM4 |
| ACP5 |
| HLA-DRA |
| FCRL3 |
| OSBPL3 |
| ICOS |
| FAM3C |
| PTPN11 |
| CKS2 |
| GALM |
| SNX9 |
| IRF4 |
| STMN1 |
| PRDM1 |
| CD2BP2 |
| RAB27A |
| DUSP4 |
| PHLDA1 |
| ITM2A |
| IFI35 |
| ISG15 |
| STAT3 |
| WARS |
| SYNGR2 |
| GBP2 |
| LYST |
| BST2 |
| PARK7 |
