# Supplementary material for: DNA sensing via the cGAS/STING pathway activates the immunoproteasome and adaptive T‐cell immunity
Source: EMBO J. 2023 Mar 13;42(8):e110597. doi: 10.15252/embj.2022110597 (PMC10106989; doi:10.15252/embj.2022110597)
Supplement: Supplementary file 5 — Table EV4 [file EMBJ-42-e110597-s005.docx]

**Table EV4. List of siRNAs**

| siRNA | Antisense Sequence | Sense Sequence |
| --- | --- | --- |
| STING | rCrCrArGrCrCrCrArCrUrGrUrGrArUrUrGrUrArUrArCrArGrCrUrA | rGrCrUrGrUrArUrArCrArArUrCrArCrArGrUrGrGrGrCrUGG |
| cGAS | rCrCrGrArGrArUrArUrCrUrUrUrGrCrGrUrUrUrCrArArUrCrUrCrA | rArGrArUrUrGrArArArCrGrCrArArArGrArUrArUrCrUrCGG |
| \| TFAM \|  \|  \| \| --- \| --- \| --- \| | dTdTCCUCUCGUCUACCGACUU | GGAAGAGCAGAUGGCUGAAdTdT |
