# Supplementary material for: DNA sensing via the cGAS/STING pathway activates the immunoproteasome and adaptive T‐cell immunity
Source: EMBO J. 2023 Mar 13;42(8):e110597. doi: 10.15252/embj.2022110597 (PMC10106989; doi:10.15252/embj.2022110597)
Supplement: Supplementary file 6 — Table EV5 [file EMBJ-42-e110597-s011.docx]

Table EV5 Primer list

| Gene | Forward Primer (5’-3’) | Reverse Primer (3’-5’) |
| --- | --- | --- |
| mouse *Psmb8* | TGCTTATGCTACCCACAGAGACAA | TTCACTTTCACCCAACCGTC |
| mouse *Psmb9* | GTACCGTGAGGACTTGTTAGCGC | GGCTGTCGAATTAGCATCCCT |
| mouse *Psmb10* | GAAGACCGGTTCCAGCCAA | CACTCAGGATCCCTGCTGTGAT |
| mouse *Nd2* | CCCATTCCACTTCTGATTACC | ATGATAGTAGAGTTGAGTAGCG |
| mouse *Nd4* | CCACTGCTAATTGCCCTCAT | CTTCAACATGGGCTTTTGGT |
| mouse *Stat1* | TGGTGAAATTGCAAGAGCTG | CAGACTTCCGTTGGTGGATT |
| mouse *Ifnb1* | TGGGAGATGTCCTCAACTGC | ACCACCACTCATTCTGAGGC |
| mouse *Rpl19* | CGGGAATCCAAGAAGATTGA | TTCAGCTTGTGGATGTGCTC |
| mouse *Tfam* | CAGGAGGCAAAGGATGATTC | CCAAGACTTCATTTCATTGTCG |
| mouse 18s rDNA | AGAAACGGCTACCACATCCA | CCCTCCAATGGATCCTCGTT |
| human *IFNB1* | ACTGCCTCAAGGACAGGATG | AGCCAGGAGGTTCTCAACAA |
| human *RPL19* | TGTACCTGAAGGTGAAGGGG | GCGTGCTTCCTTGGTCTTAG |
