# Supplementary figures and images for: DNA sensing via the cGAS/STING pathway activates the immunoproteasome and adaptive T‐cell immunity
Source: EMBO J. 2023 Mar 13;42(8):e110597. doi: 10.15252/embj.2022110597 (PMC10106989; doi:10.15252/embj.2022110597)

Fig. 1A

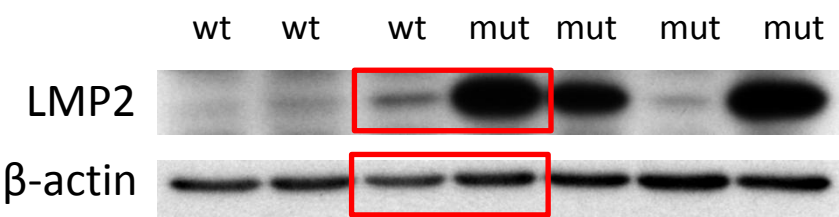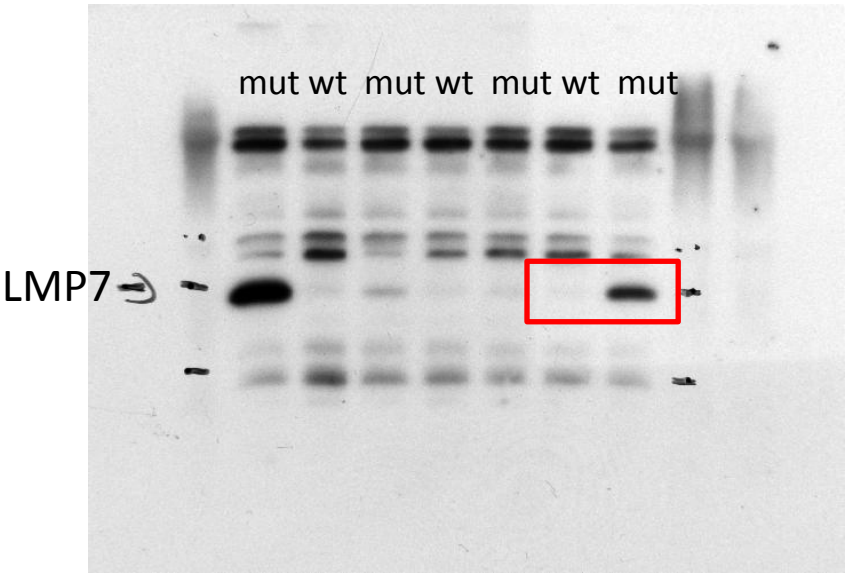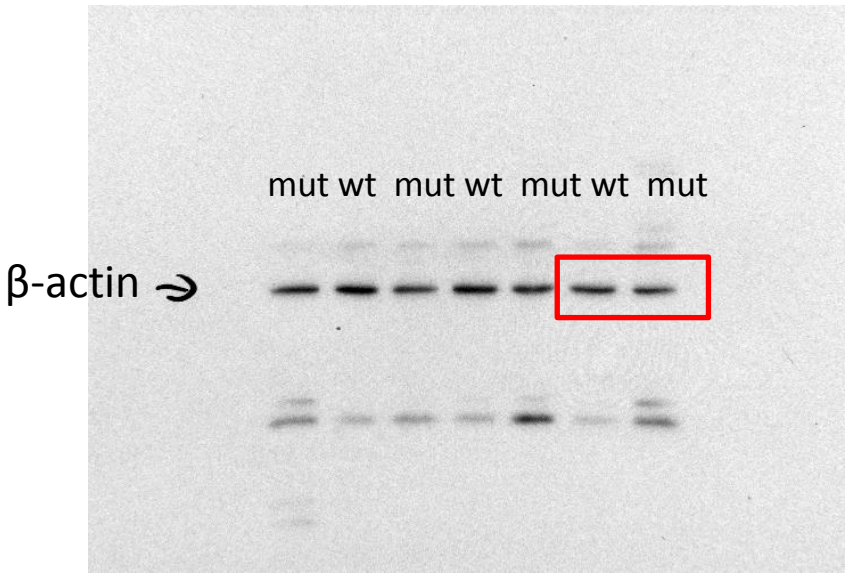

Supplement: Supplementary file 8 — Source Data for Figure 1 [file EMBJ-42-e110597-s006.zip › SourceData_Figure 1/SourceData_Figure 1A.pdf]

Fig.1C

LW124

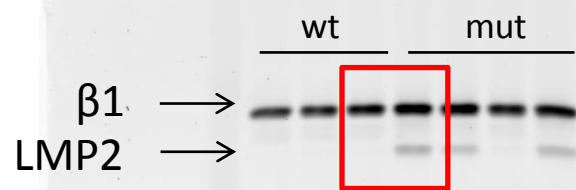

Total protein

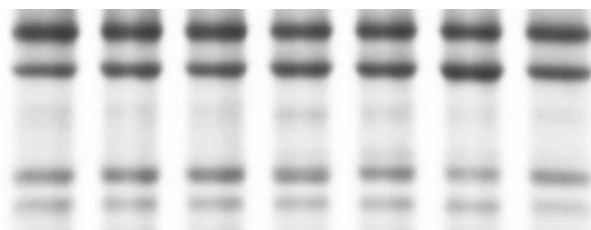

MVB127

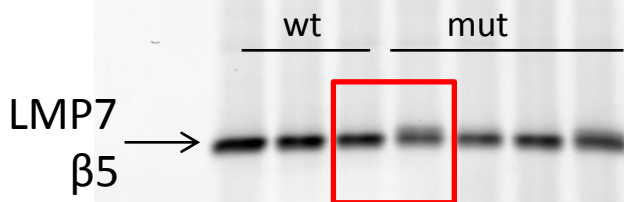

MV151

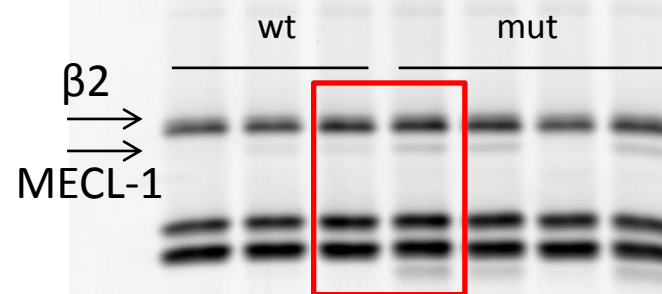

Total protein

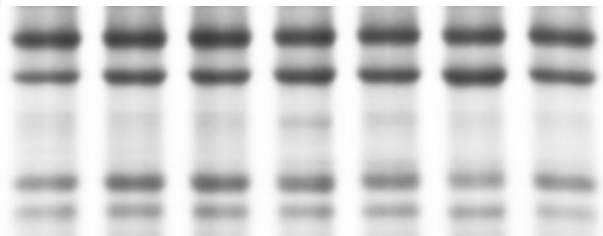

Supplement: Supplementary file 8 — Source Data for Figure 1 [file EMBJ-42-e110597-s006.zip › SourceData_Figure 1/SourceData_Figure 1C.pdf]

Fig.1C

LW124

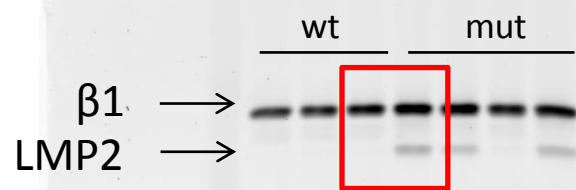

Total protein

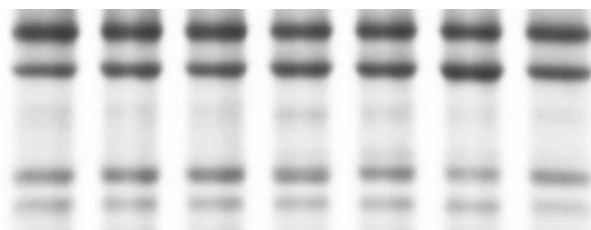

MVB127

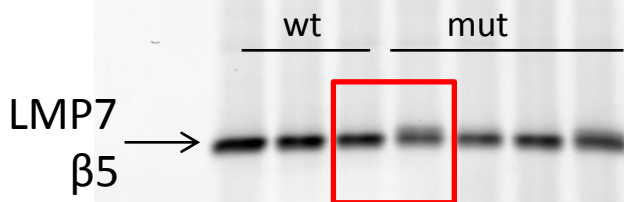

MV151

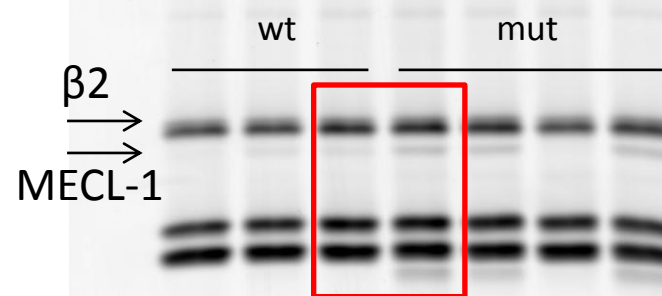

Total protein

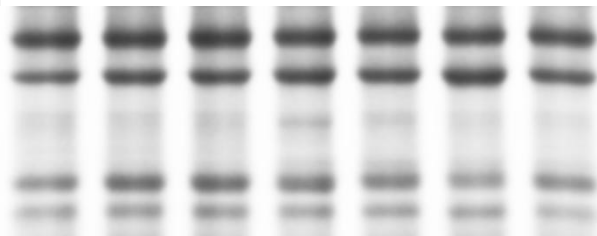

Supplement: Supplementary file 9 — Source Data for Figure 2 [file EMBJ-42-e110597-s004.zip › SourceData_Figure 2/SourceData_Figure 2D.pdf]

Fig. 2G

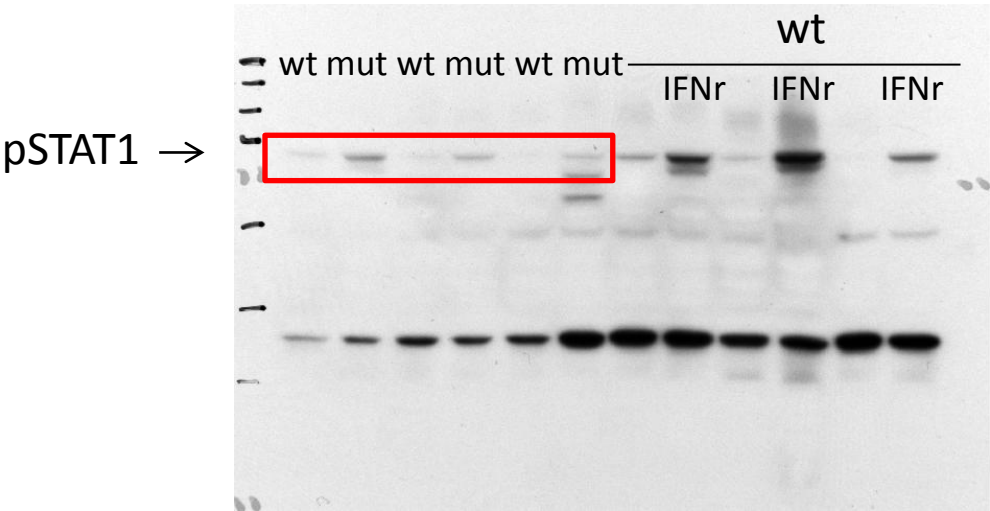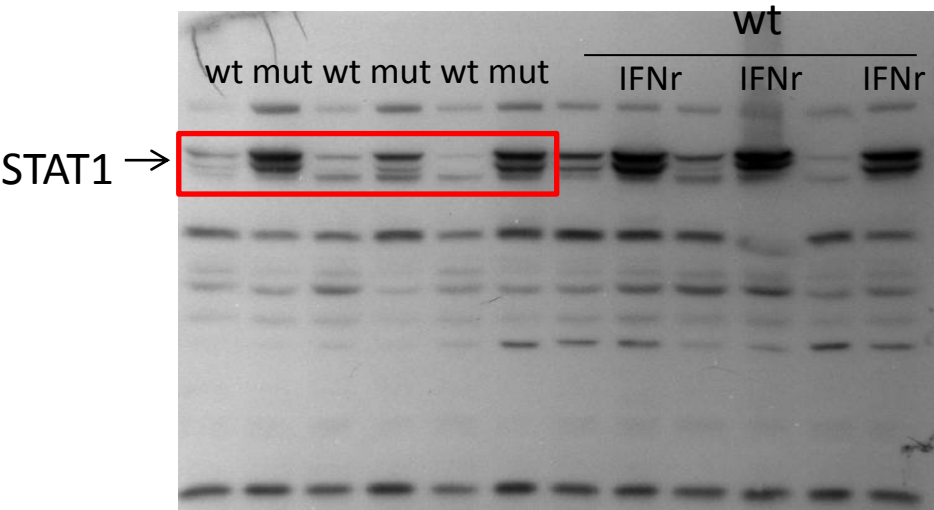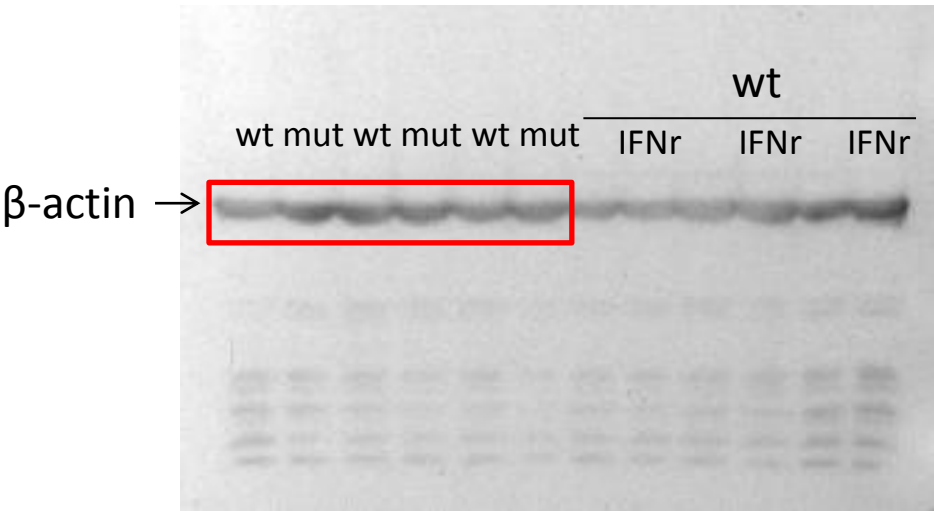

Supplement: Supplementary file 9 — Source Data for Figure 2 [file EMBJ-42-e110597-s004.zip › SourceData_Figure 2/SourceData_Figure 2G.pdf]

Fig. 3A

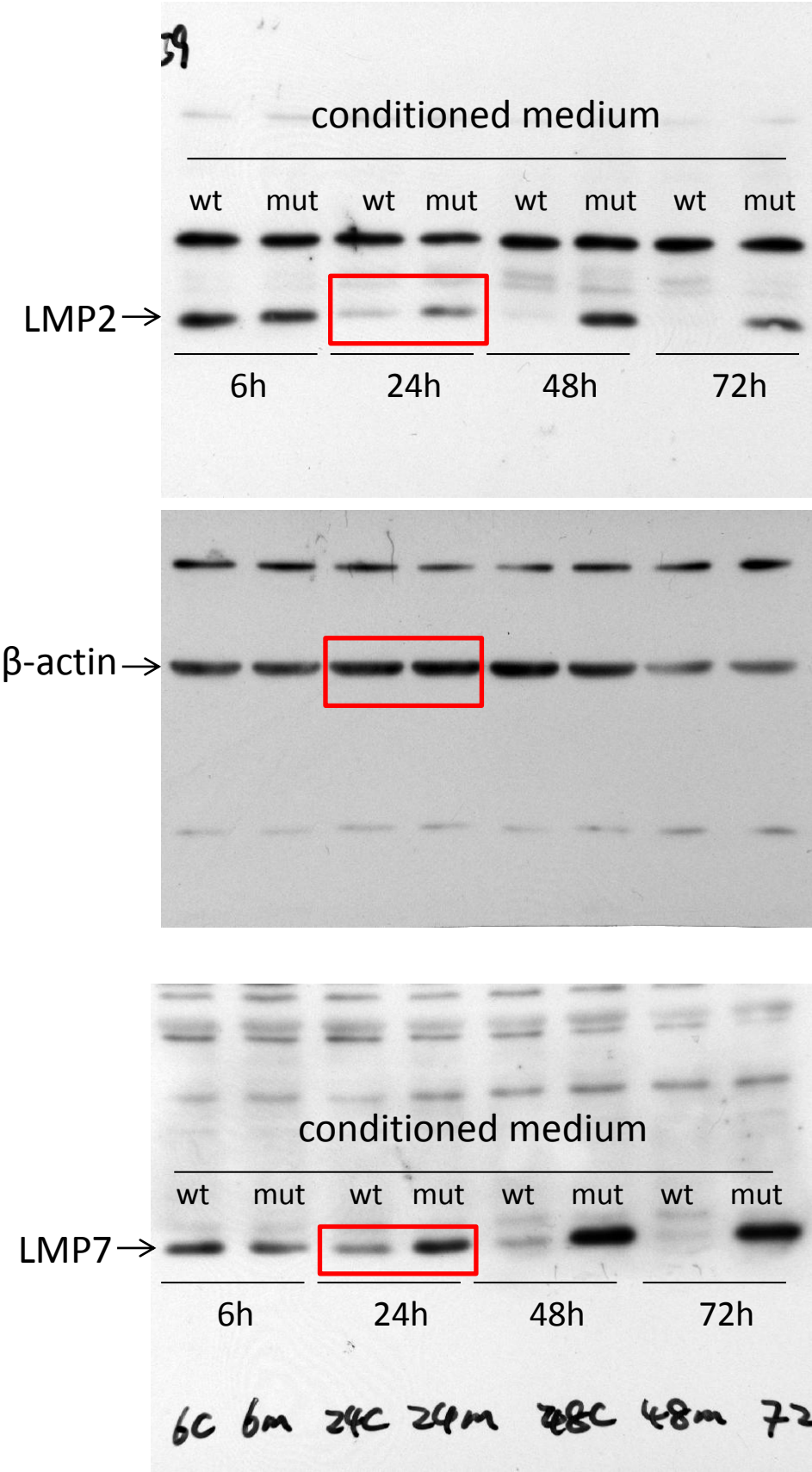

Fig. 3A

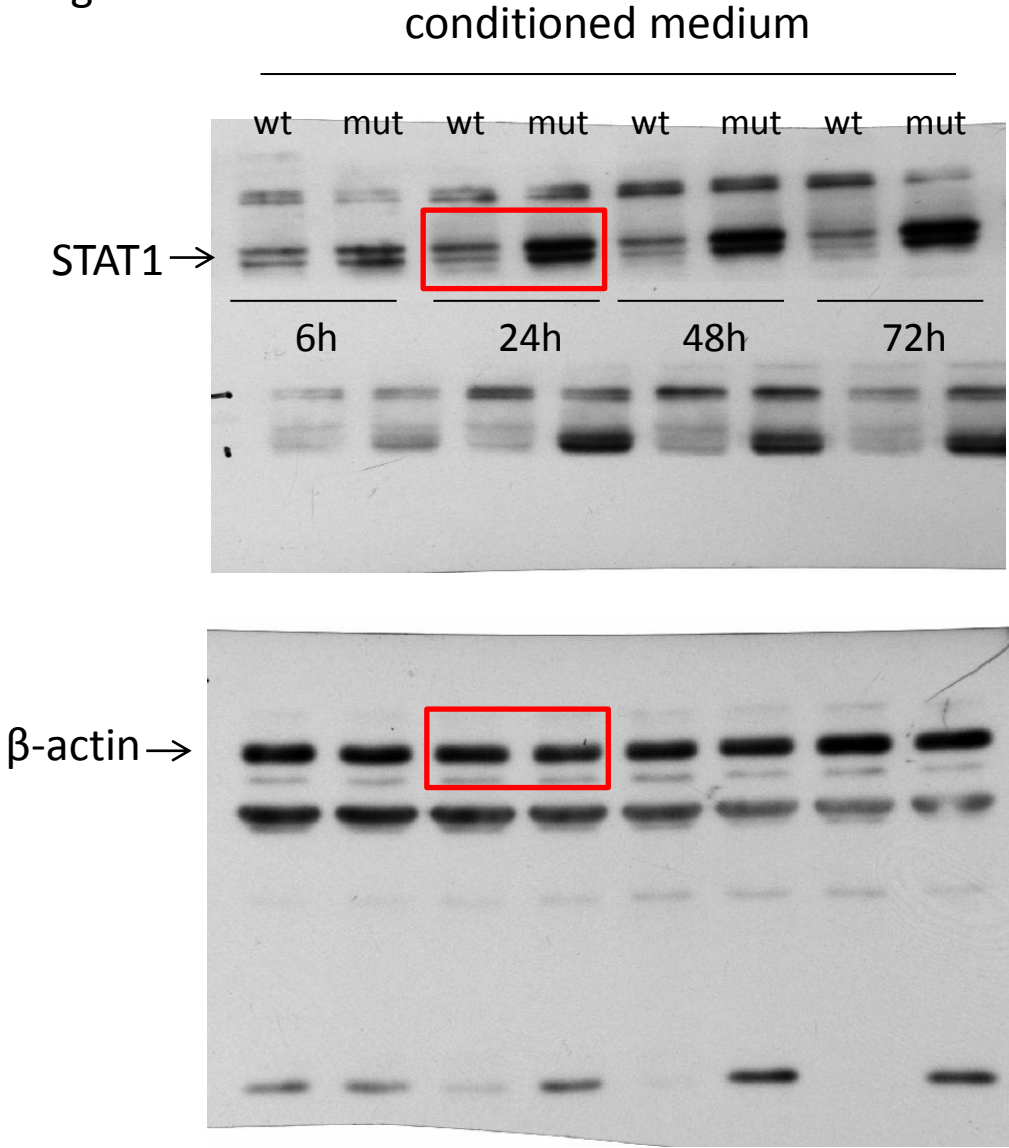

Supplement: Supplementary file 10 — Source Data for Figure 3 [file EMBJ-42-e110597-s015.zip › SourceData_Figure 3/SourceData_Figure 3A.pdf]

Fig. 3B

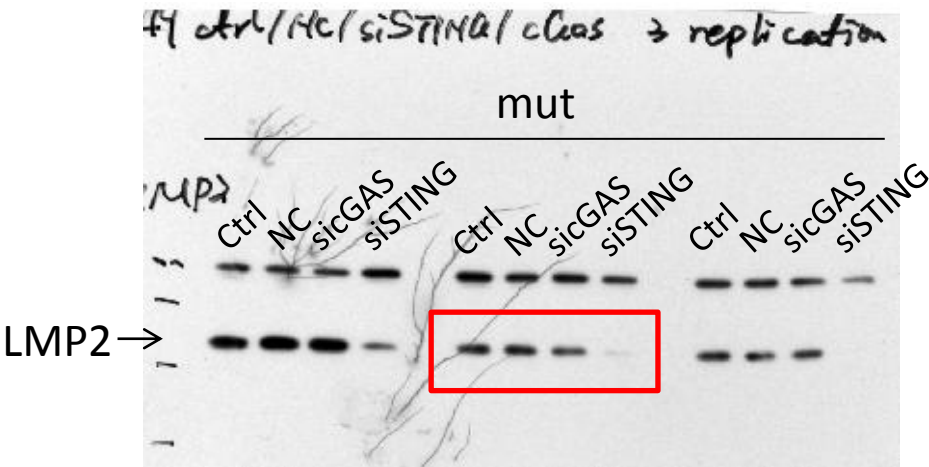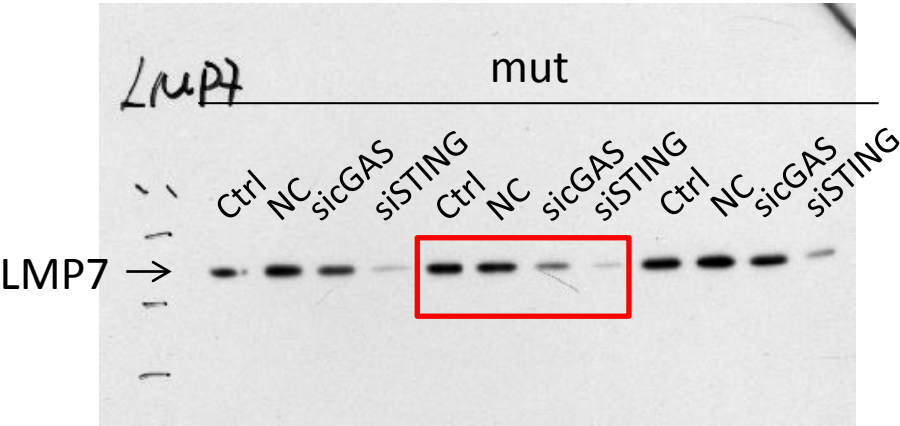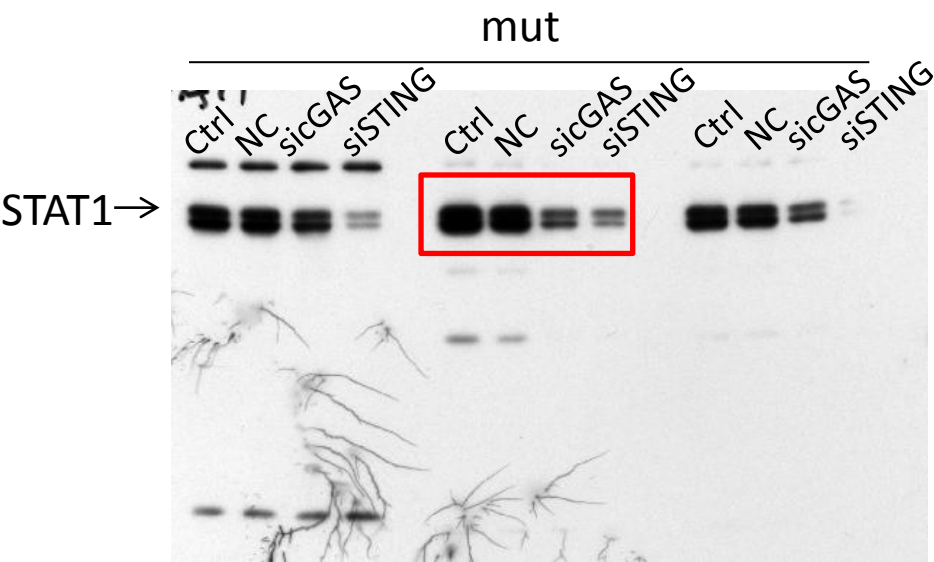

Supplement: Supplementary file 10 — Source Data for Figure 3 [file EMBJ-42-e110597-s015.zip › SourceData_Figure 3/SourceData_Figure 3B.pdf]

Fig. 3D

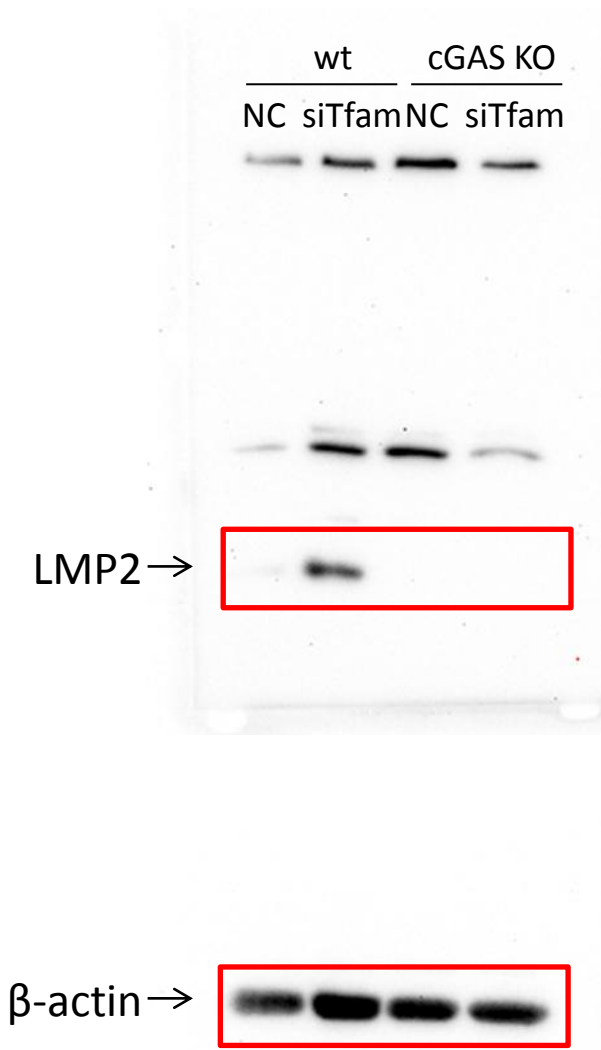

Supplement: Supplementary file 10 — Source Data for Figure 3 [file EMBJ-42-e110597-s015.zip › SourceData_Figure 3/SourceData_Figure 3D.pdf]

Fig. 3E

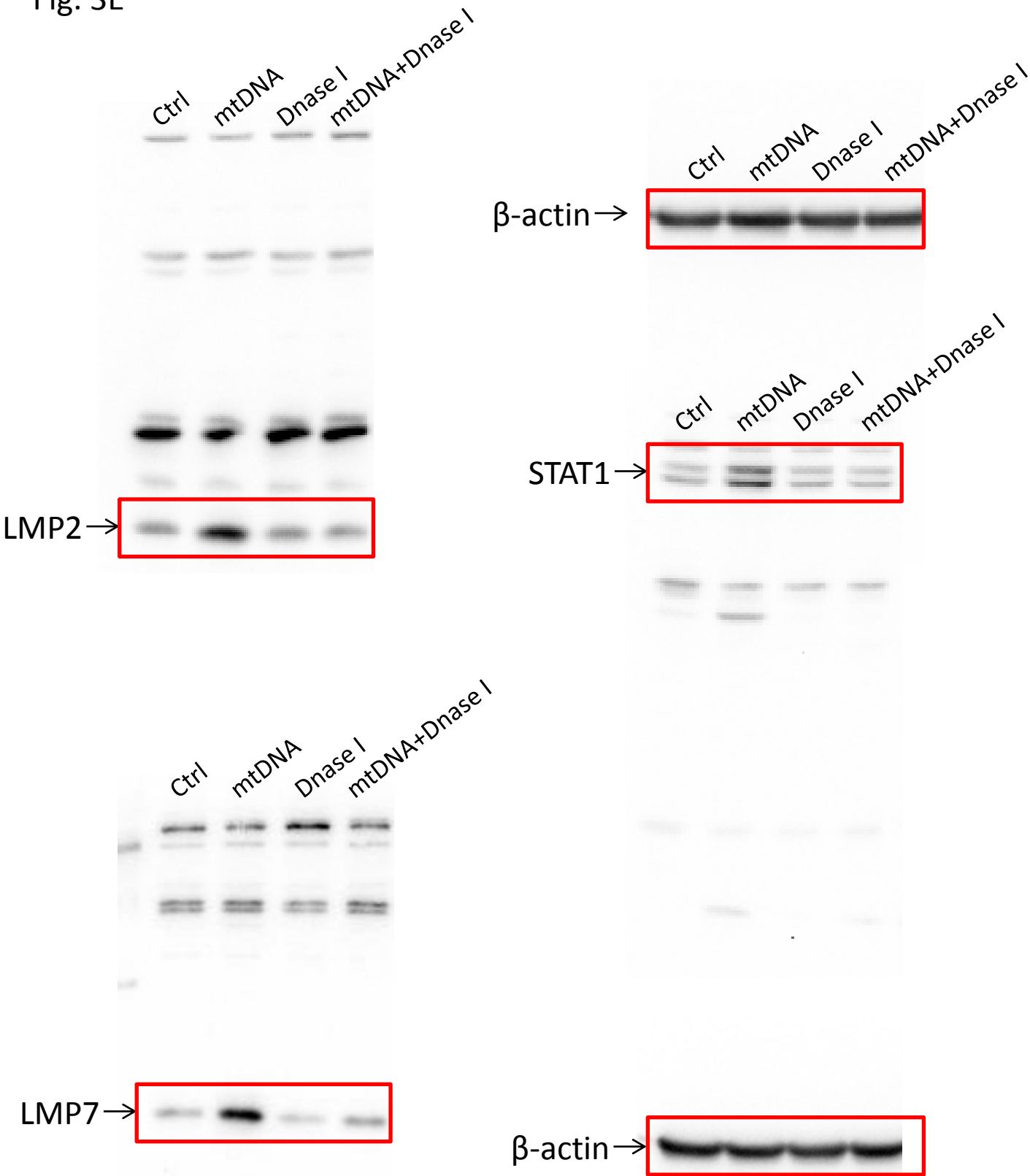

Supplement: Supplementary file 10 — Source Data for Figure 3 [file EMBJ-42-e110597-s015.zip › SourceData_Figure 3/SourceData_Figure 3E.pdf]

Fig. 3F

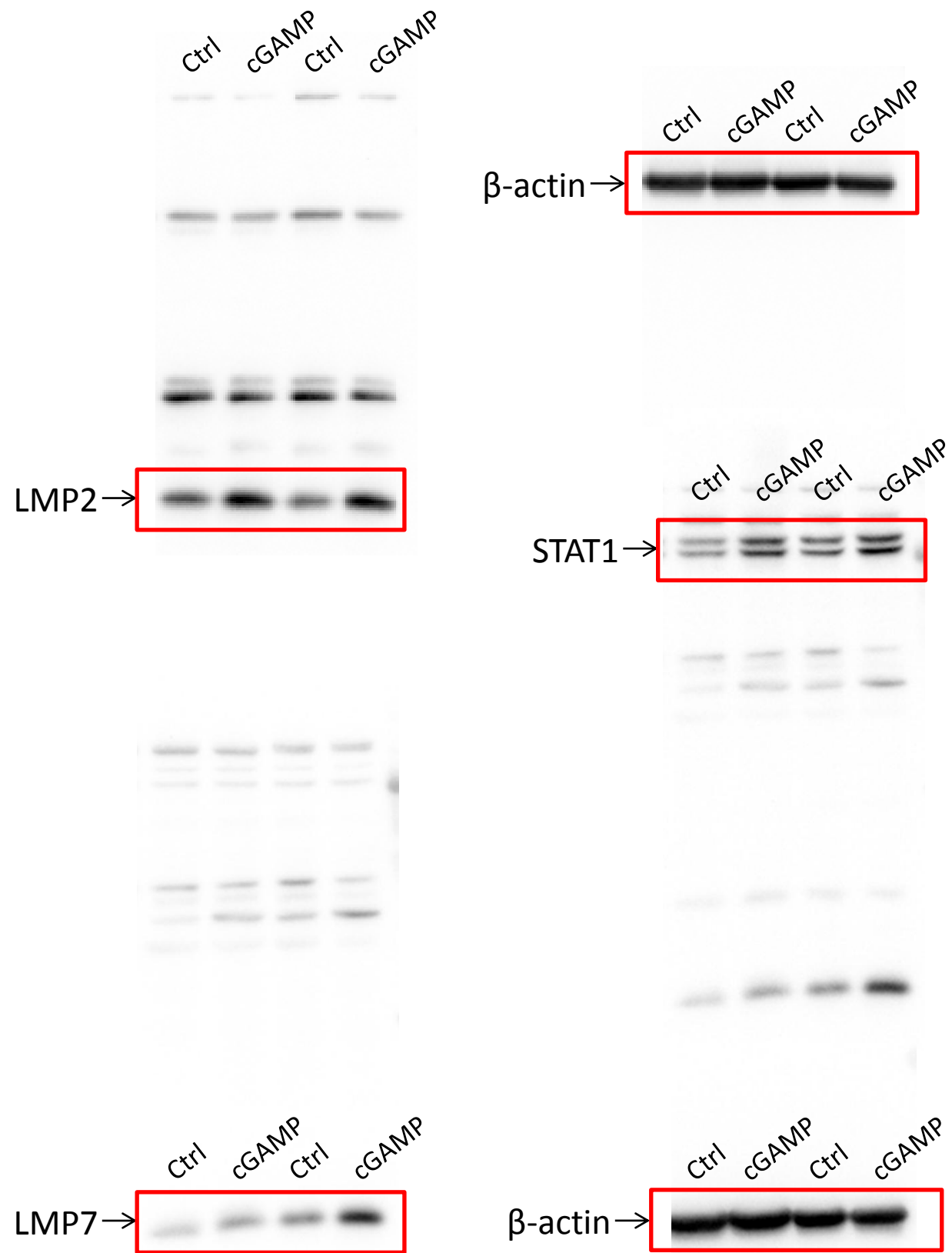

Supplement: Supplementary file 10 — Source Data for Figure 3 [file EMBJ-42-e110597-s015.zip › SourceData_Figure 3/SourceData_Figure 3F.pdf]

Fig. 3G

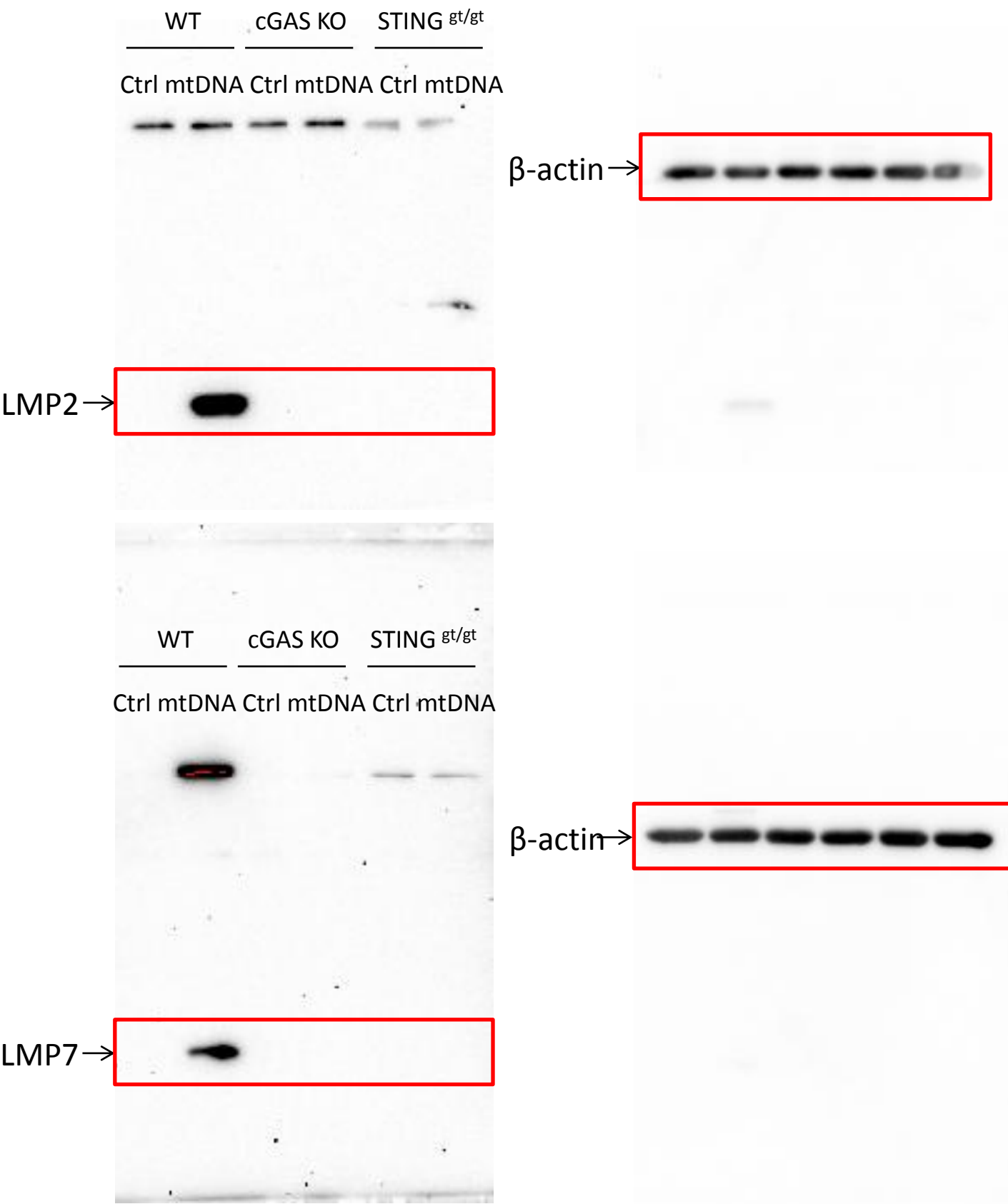

Supplement: Supplementary file 10 — Source Data for Figure 3 [file EMBJ-42-e110597-s015.zip › SourceData_Figure 3/SourceData_Figure 3G.pdf]

Fig. 6A

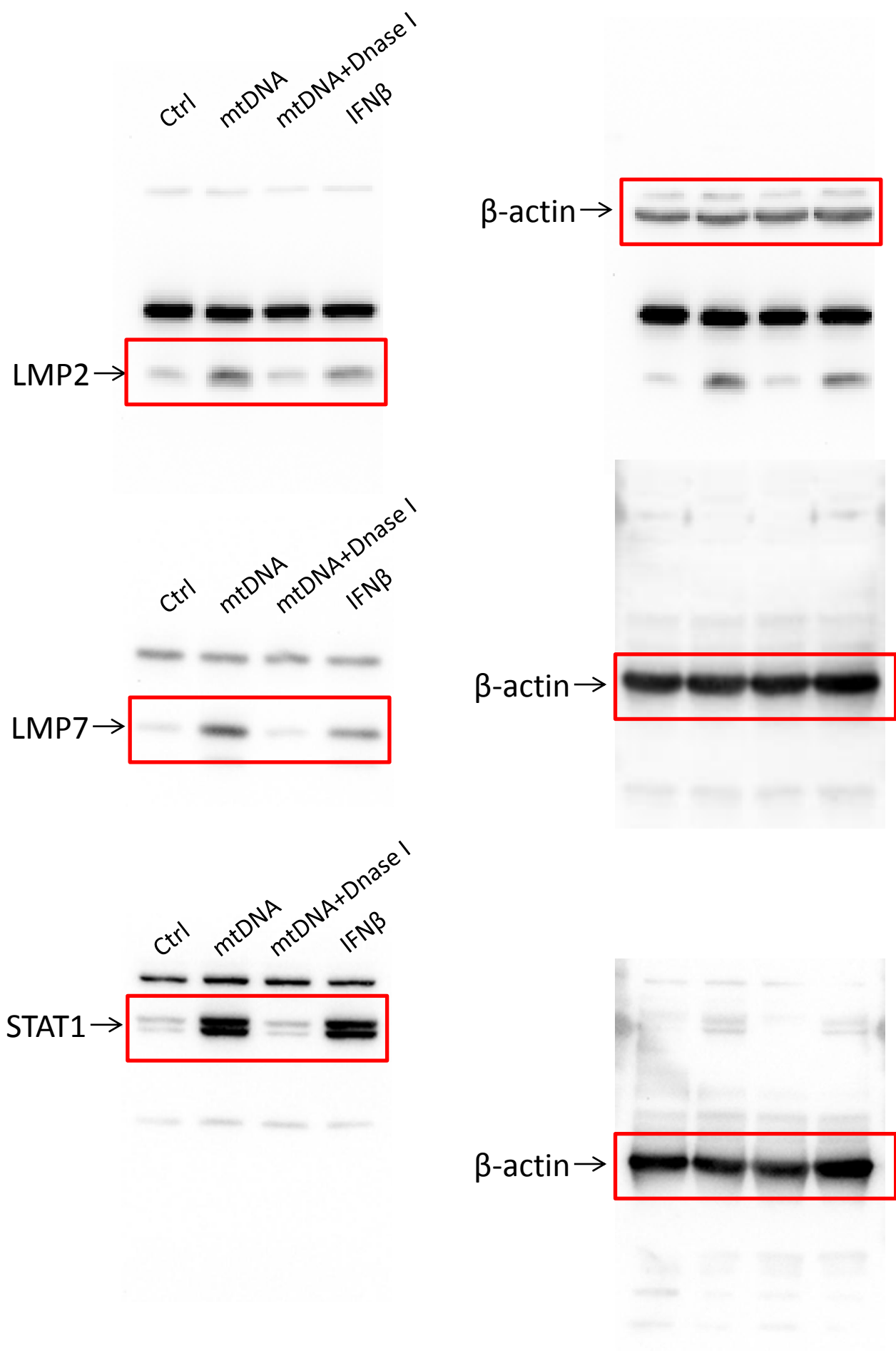

Supplement: Supplementary file 13 — Source Data for Figure 6 [file EMBJ-42-e110597-s010.zip › SourceData_Figure 6/SourceData_Figure 6A.pdf]

Fig. 6C

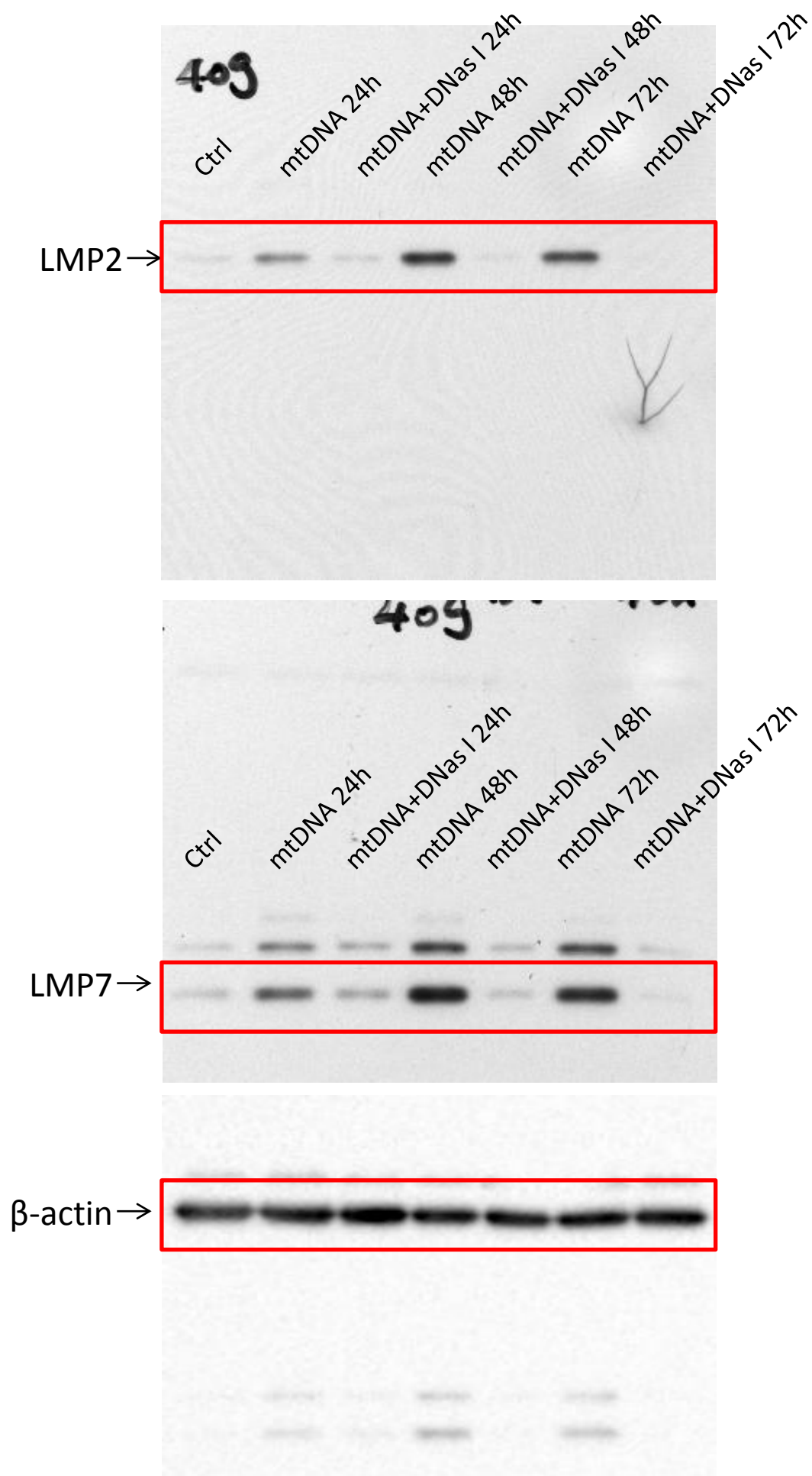

Fig. 6C

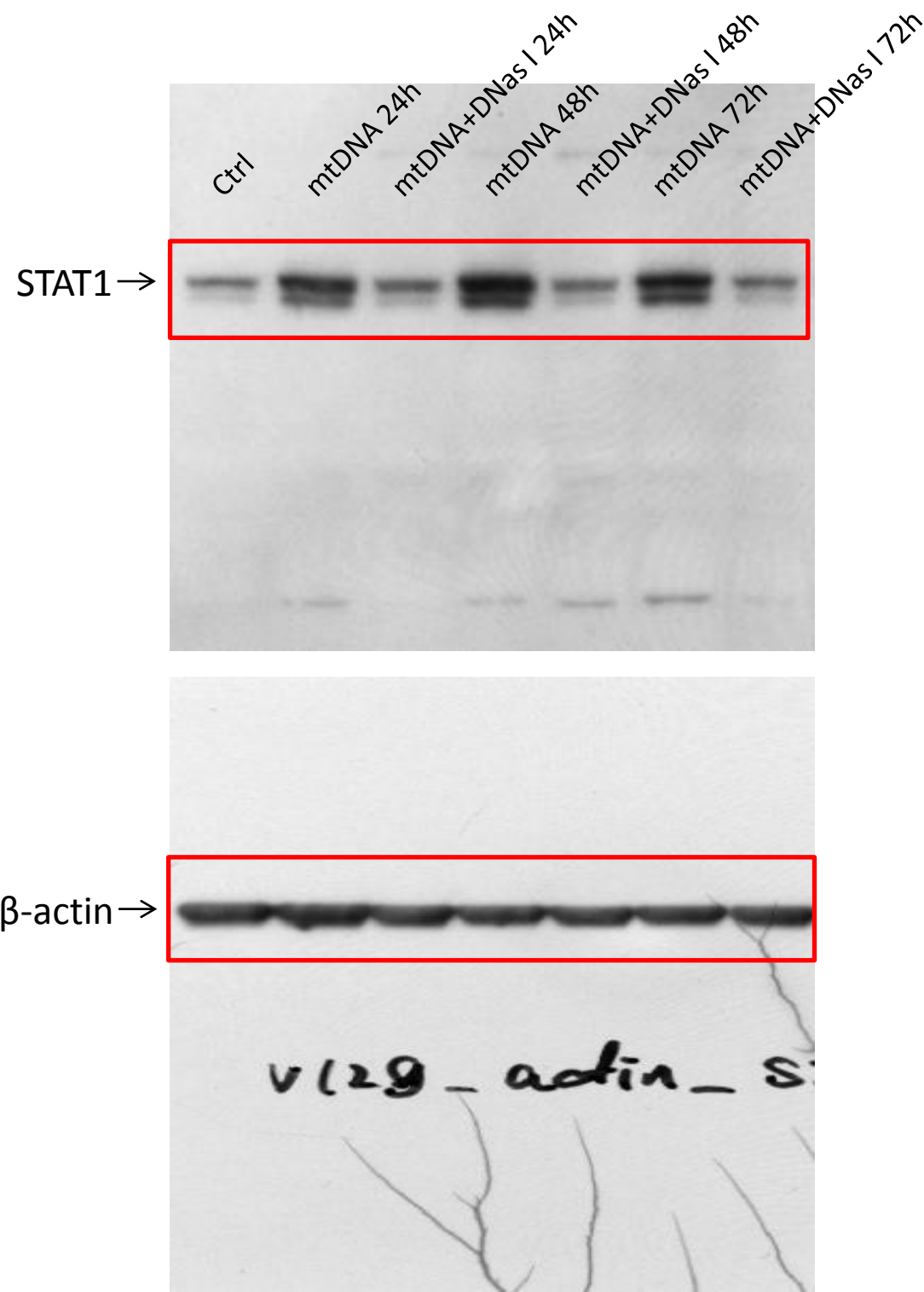

Supplement: Supplementary file 13 — Source Data for Figure 6 [file EMBJ-42-e110597-s010.zip › SourceData_Figure 6/SourceData_Figure 6C.pdf]

Fig. 6D

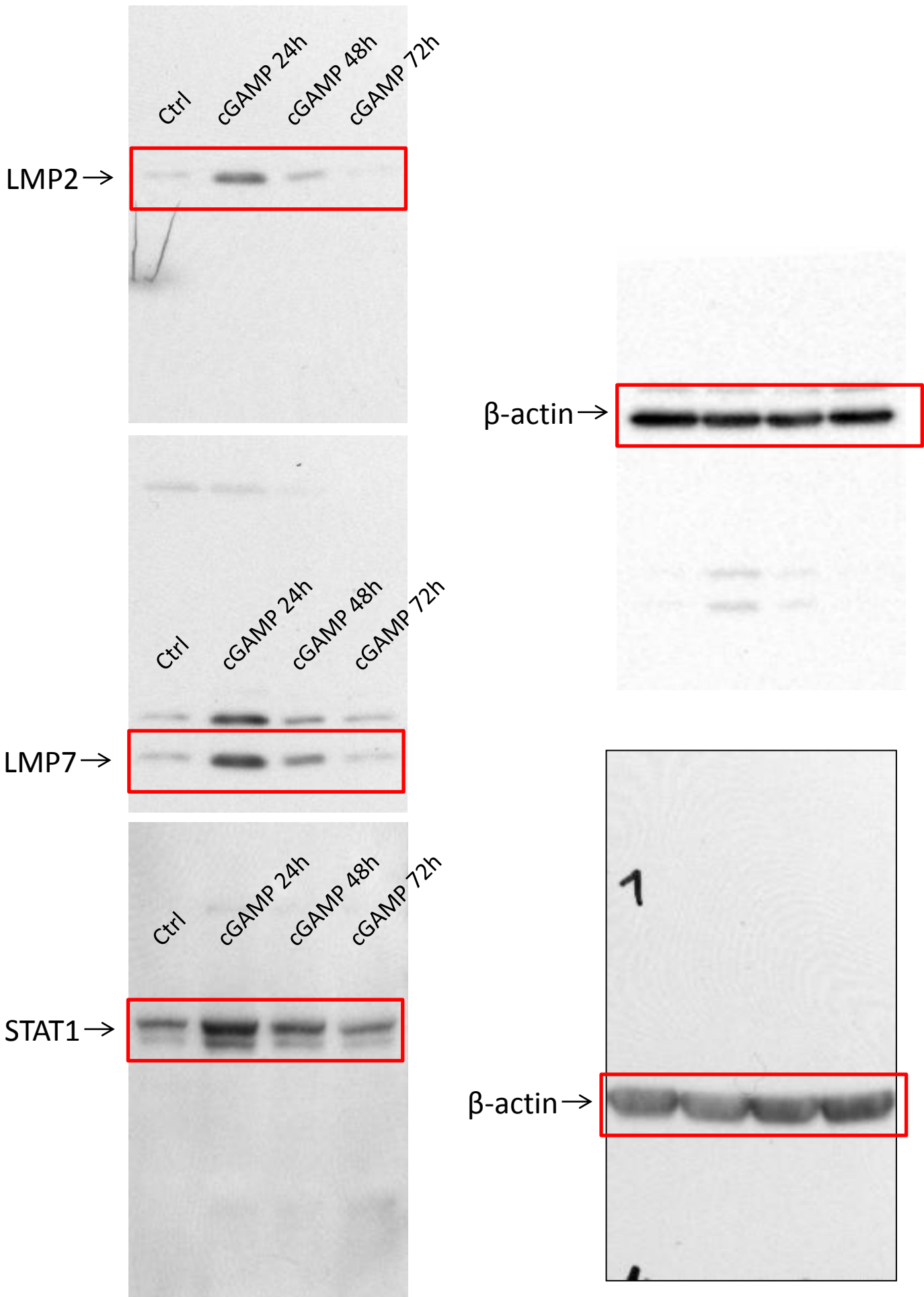

Supplement: Supplementary file 13 — Source Data for Figure 6 [file EMBJ-42-e110597-s010.zip › SourceData_Figure 6/SourceData_Figure 6D.pdf]

Fig. 6G

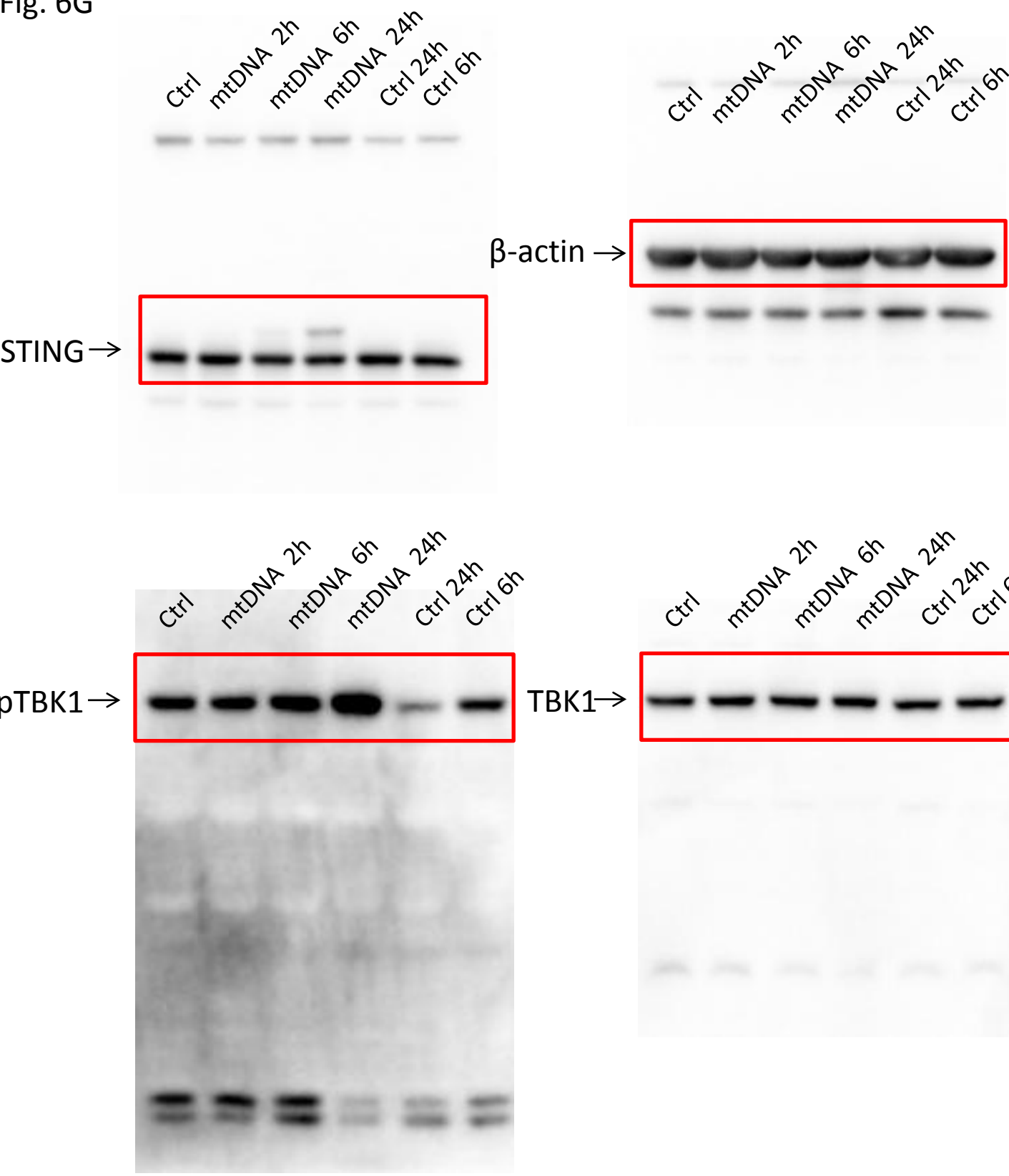

Fig. 6G

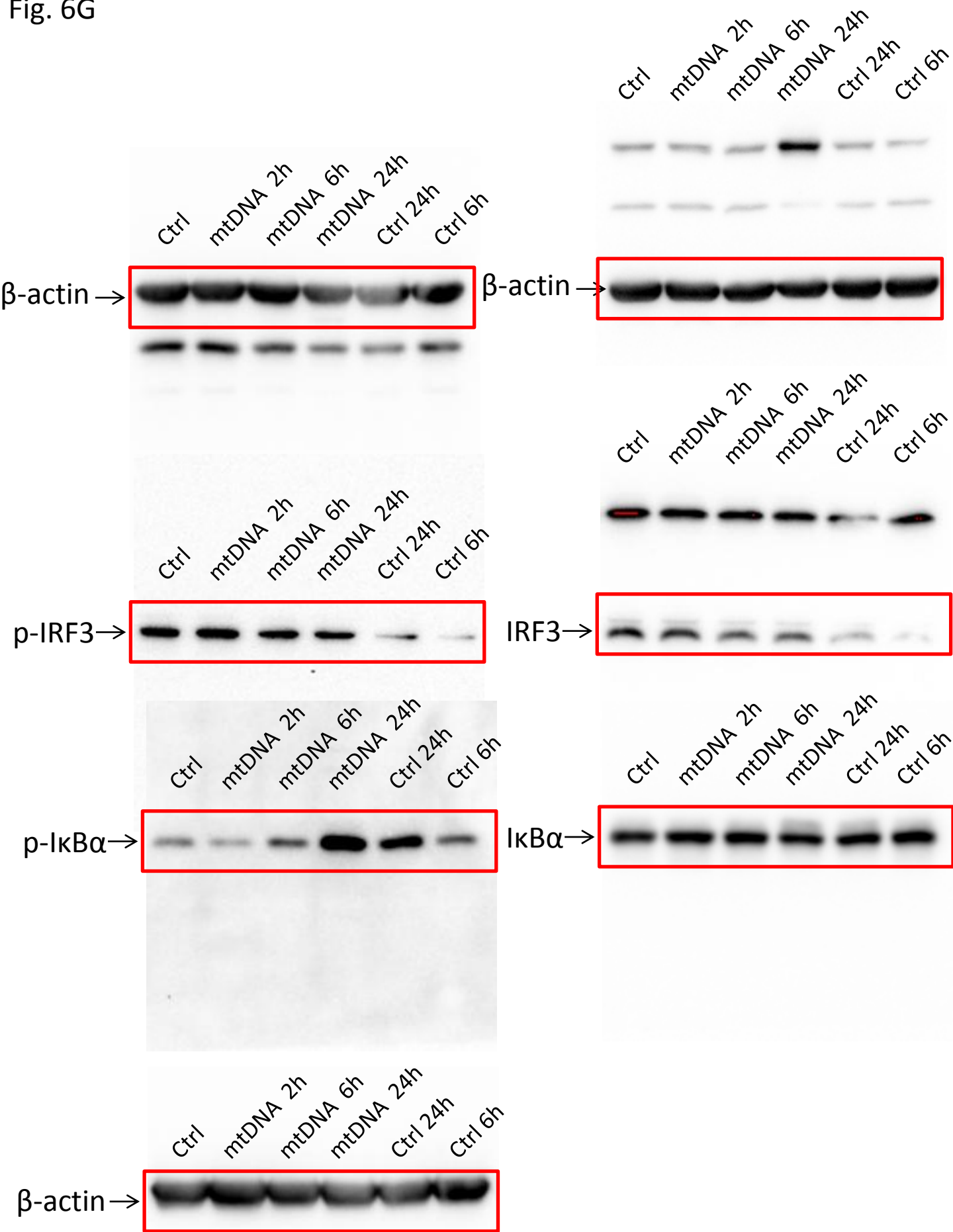

Fig. 6G

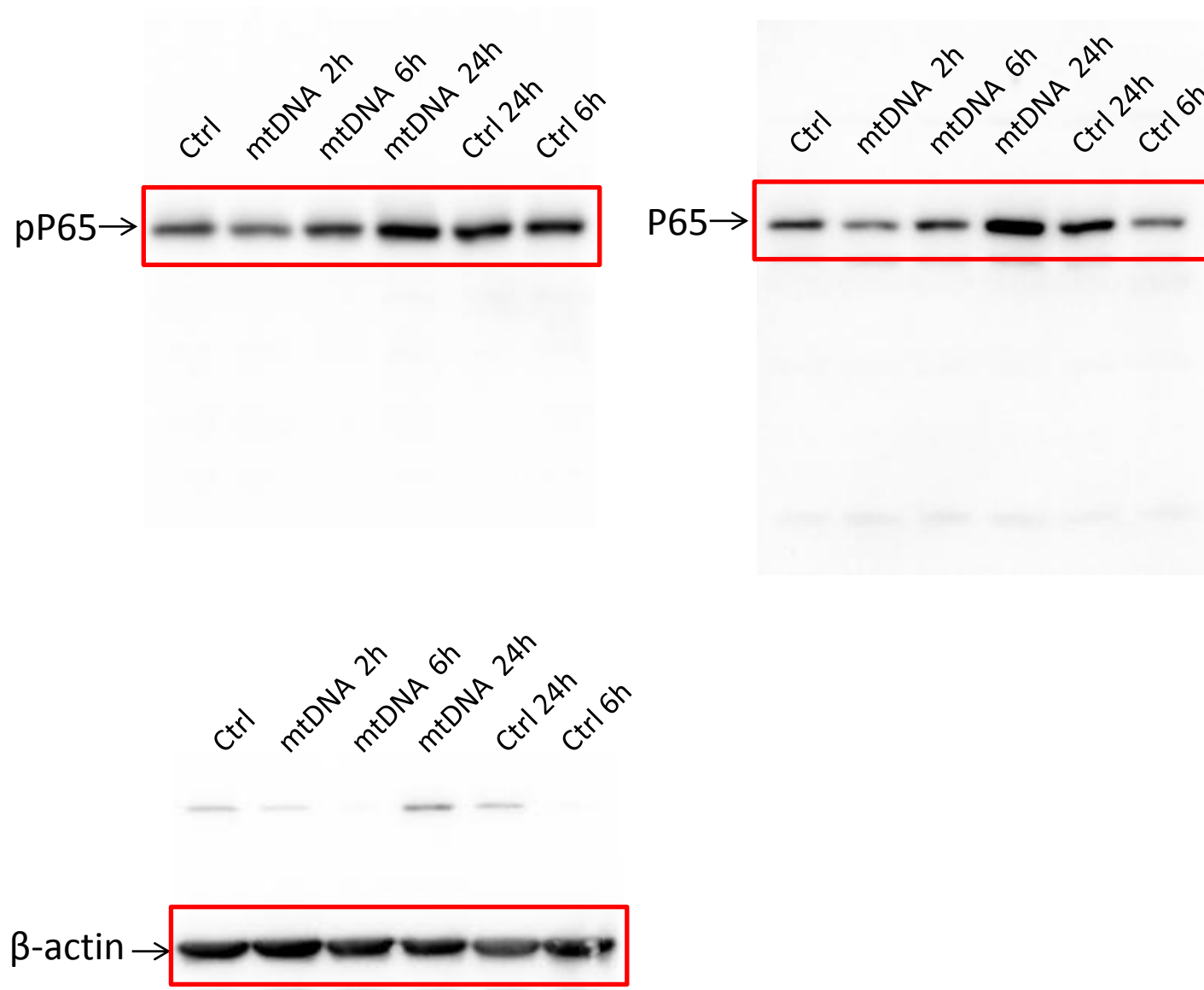

Supplement: Supplementary file 13 — Source Data for Figure 6 [file EMBJ-42-e110597-s010.zip › SourceData_Figure 6/SourceData_Figure 6G.pdf]

Fig. 6I

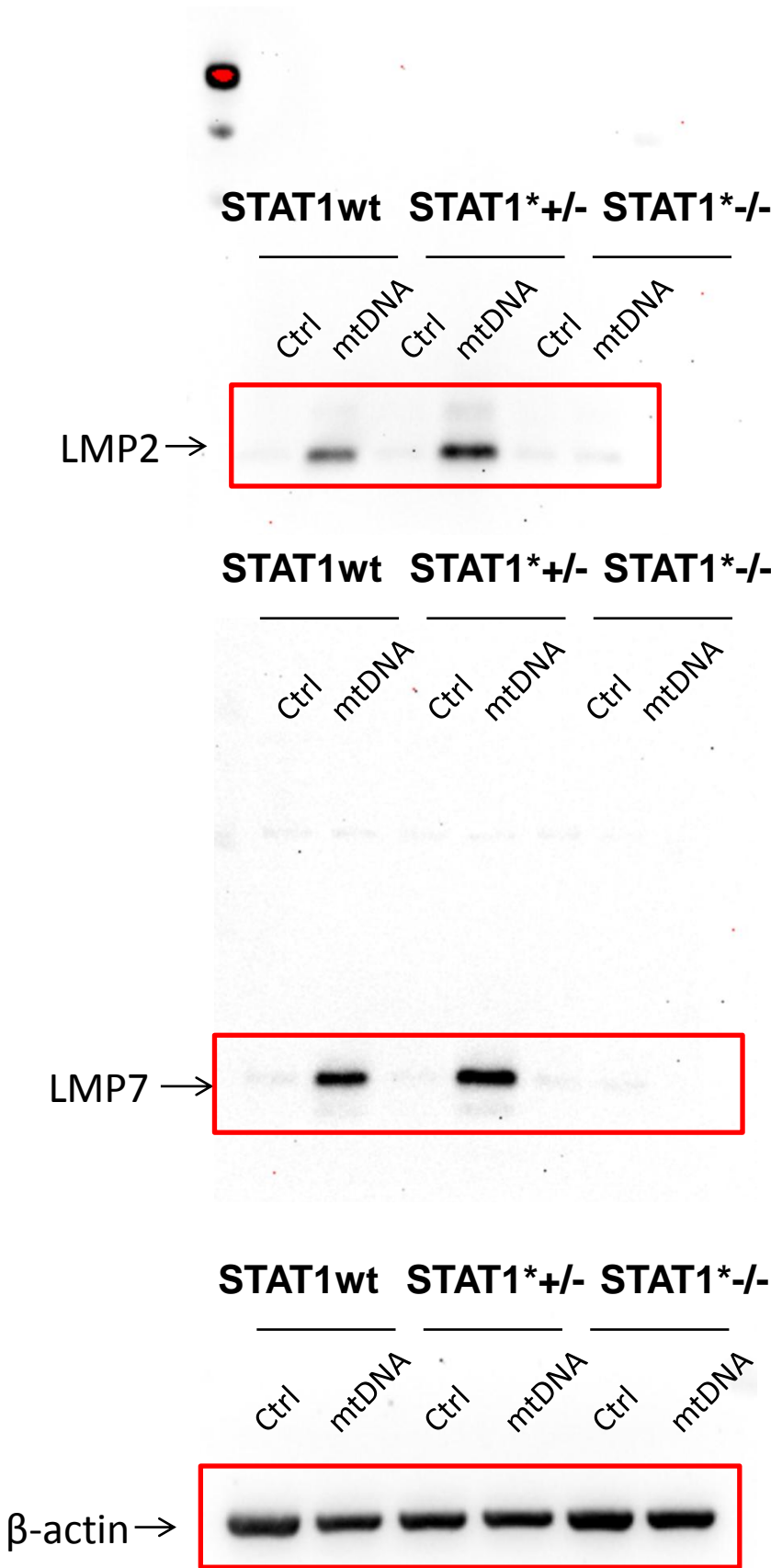

Supplement: Supplementary file 13 — Source Data for Figure 6 [file EMBJ-42-e110597-s010.zip › SourceData_Figure 6/SourceData_Figure 6I.pdf]

Fig. 8A

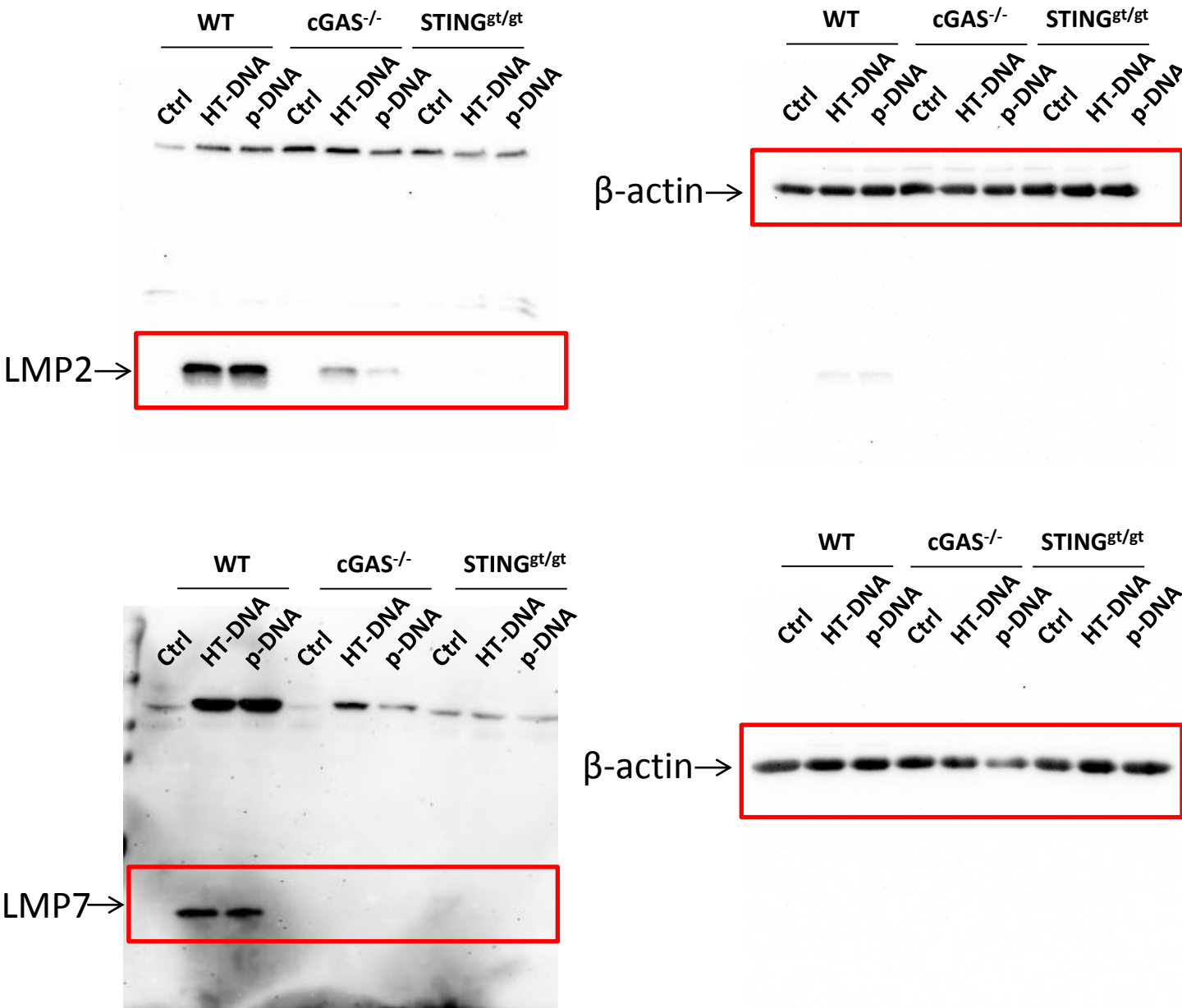

Supplement: Supplementary file 15 — Source Data for Figure 8 [file EMBJ-42-e110597-s001.zip › SourceData_Figure 8/SourceData_Figure 8A.pdf]

Fig. 8A

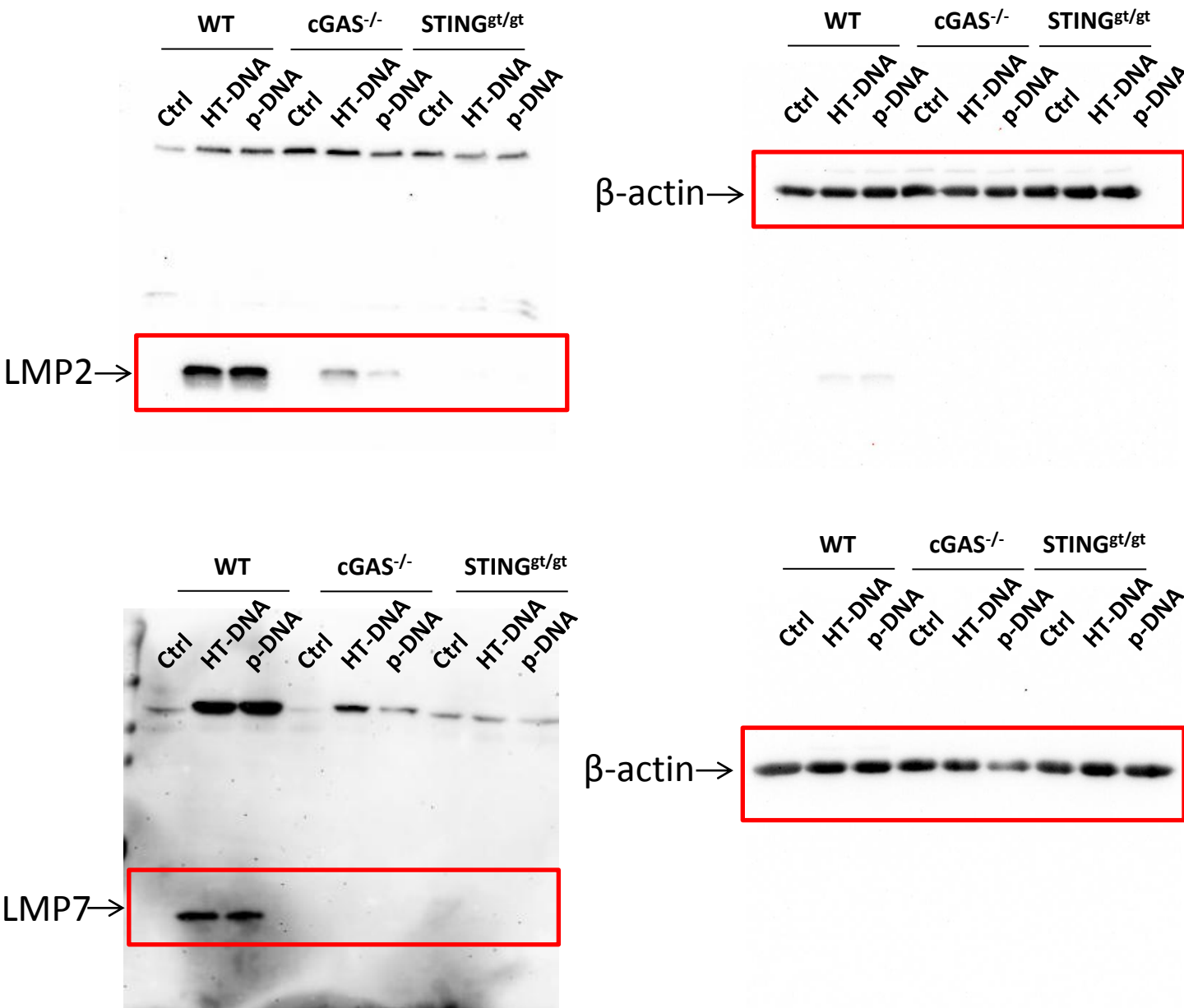

Supplement: Supplementary file 15 — Source Data for Figure 8 [file EMBJ-42-e110597-s001.zip › SourceData_Figure 8/SourceData_Figure 8C.pdf]

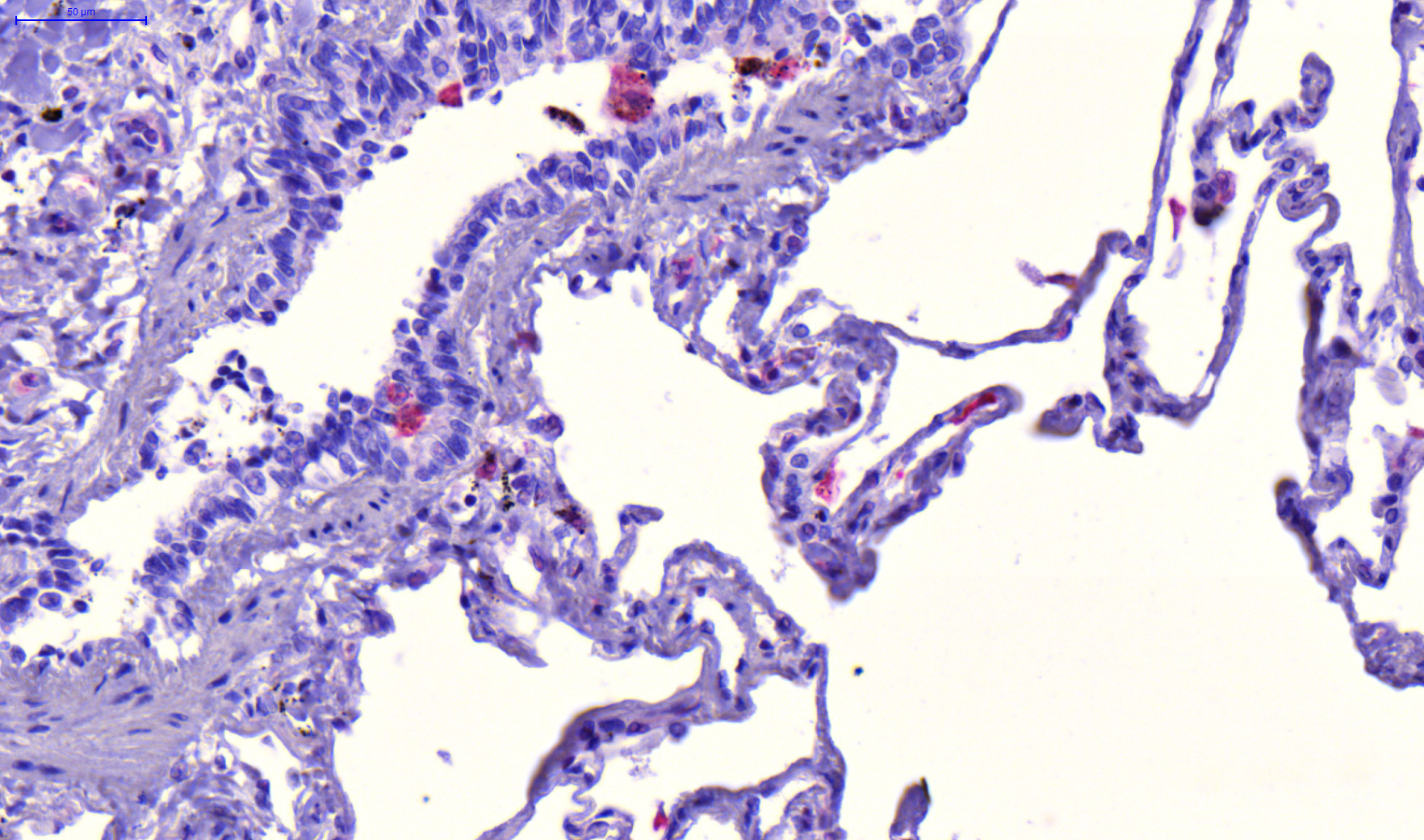

Supplement: Supplementary file 16 — Source Data for Figure 9 [file EMBJ-42-e110597-s009.zip › SourceData_Figure 9C /_IHC333-12 Gies62 LMP2 x8C Doner_40x_Bar.tif]

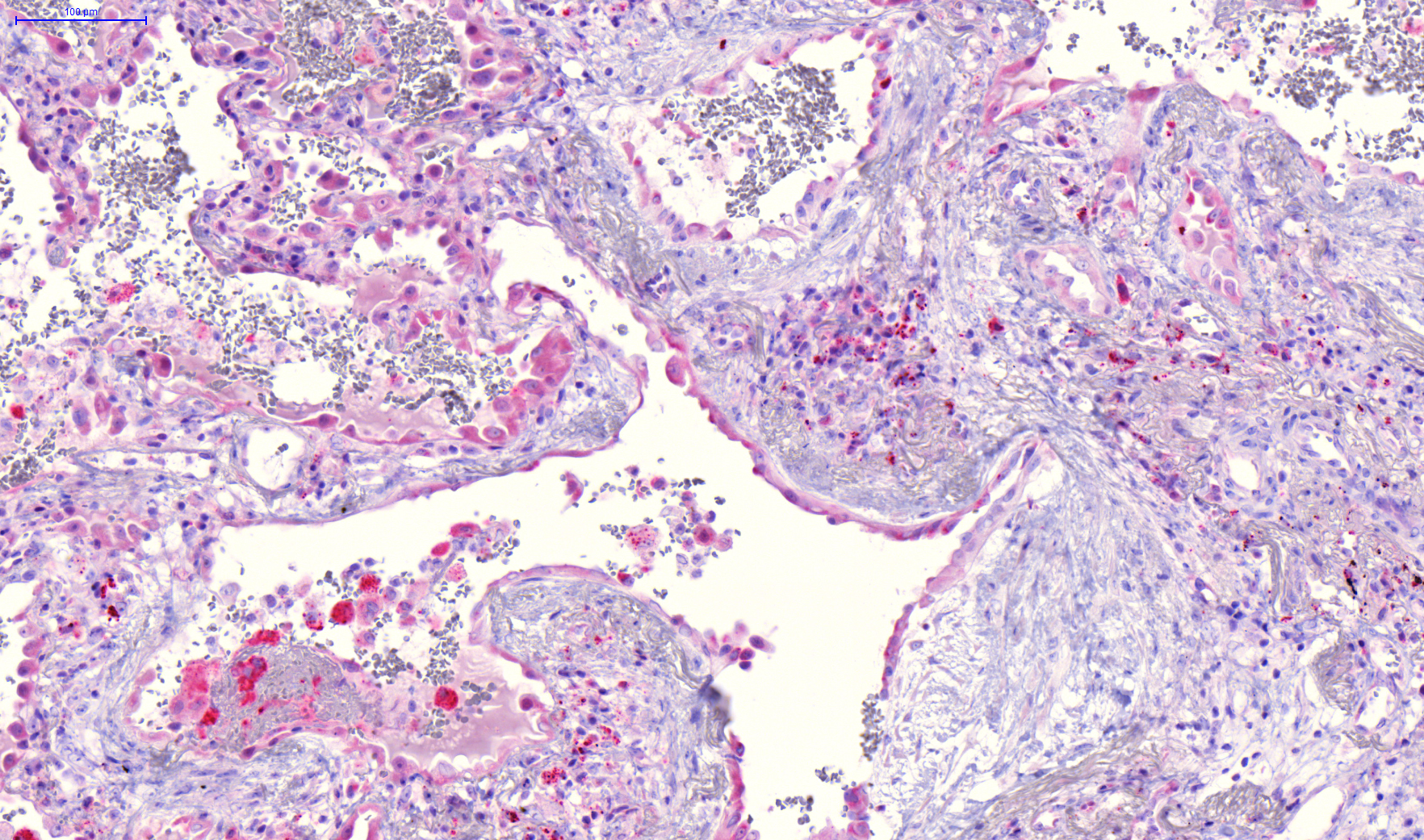

Supplement: Supplementary file 16 — Source Data for Figure 9 [file EMBJ-42-e110597-s009.zip › SourceData_Figure 9C /_IHC333-3 Gies180 LMP2 x4C IPF_2_10x_Bar.tif]

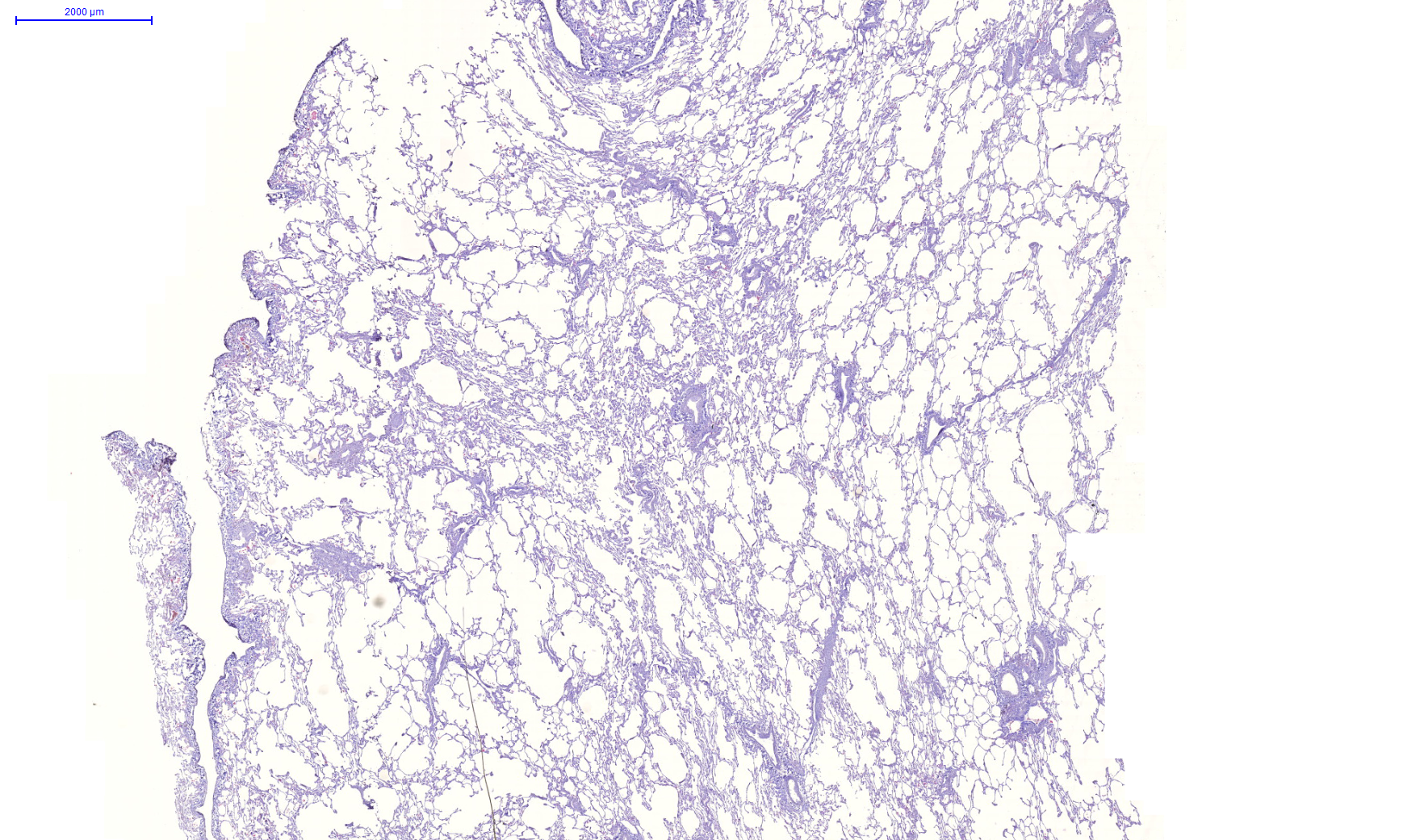

Supplement: Supplementary file 16 — Source Data for Figure 9 [file EMBJ-42-e110597-s009.zip › SourceData_Figure 9C /_IHC333-12 Gies62 LMP2 x8C Doner_4x_Bar.tif]

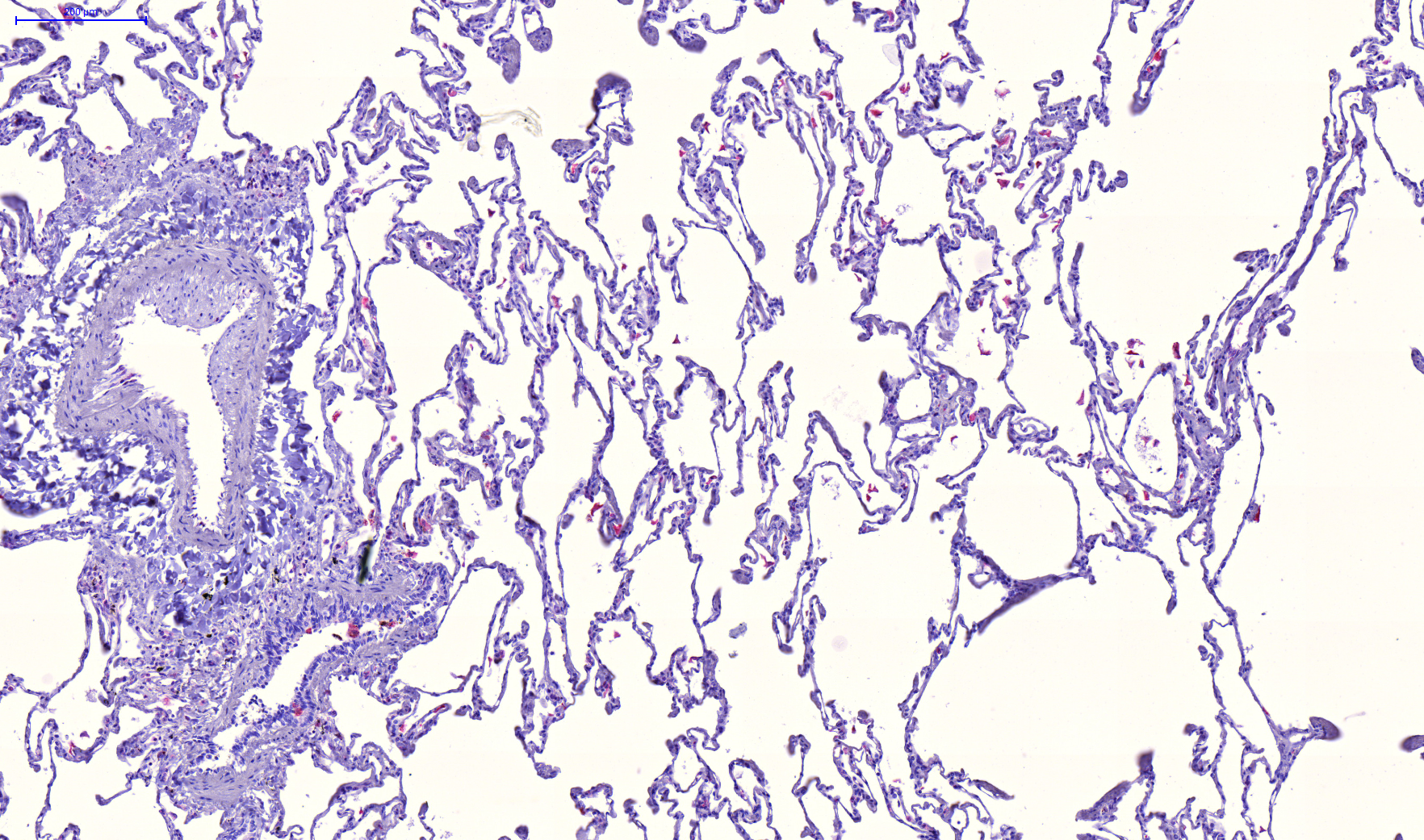

Supplement: Supplementary file 16 — Source Data for Figure 9 [file EMBJ-42-e110597-s009.zip › SourceData_Figure 9C /_IHC333-12 Gies62 LMP2 x8C Doner_10x_Bar.tif]

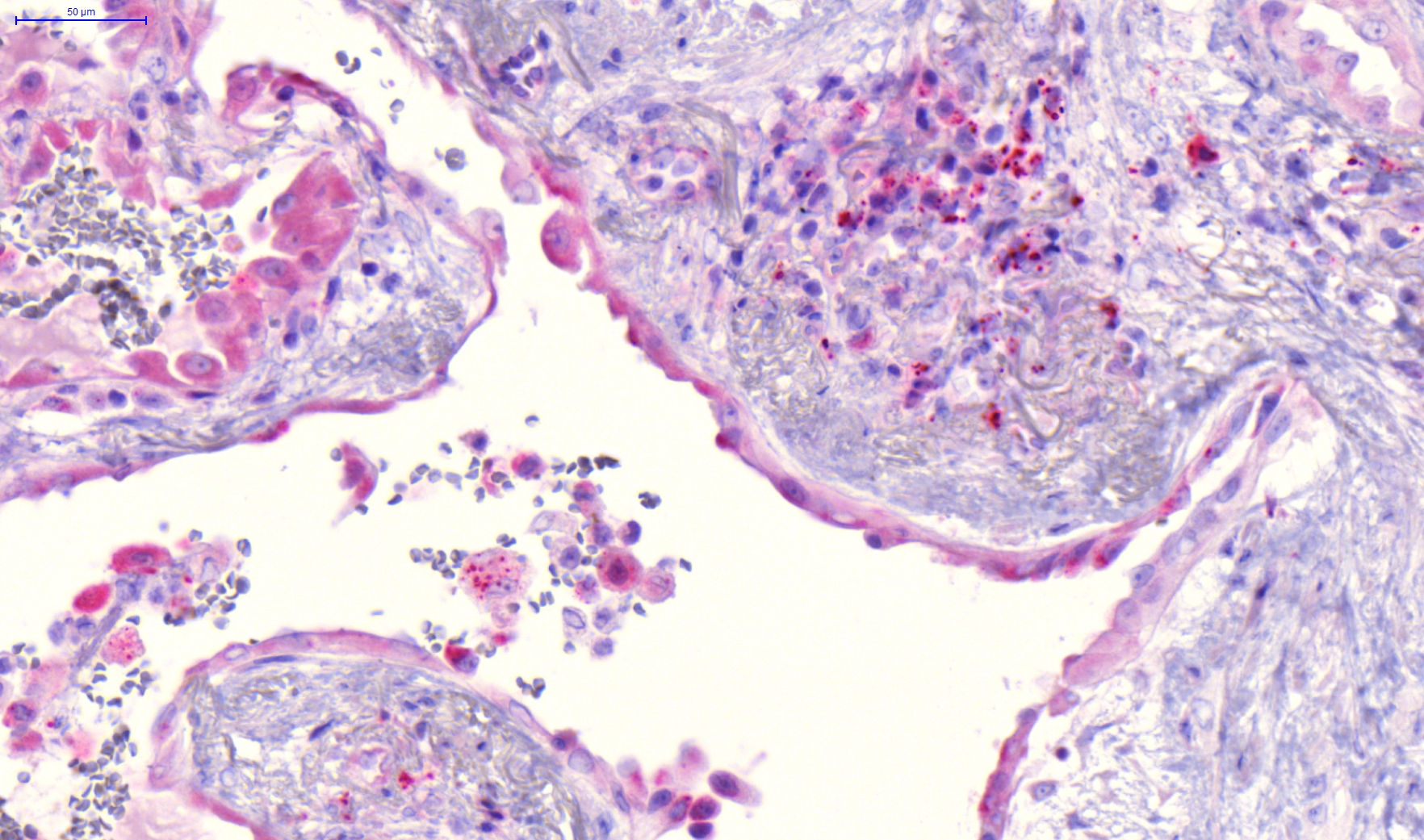

Supplement: Supplementary file 16 — Source Data for Figure 9 [file EMBJ-42-e110597-s009.zip › SourceData_Figure 9C /_IHC333-3 Gies180 LMP2 x4C IPF_2_40x_Bar.tif]

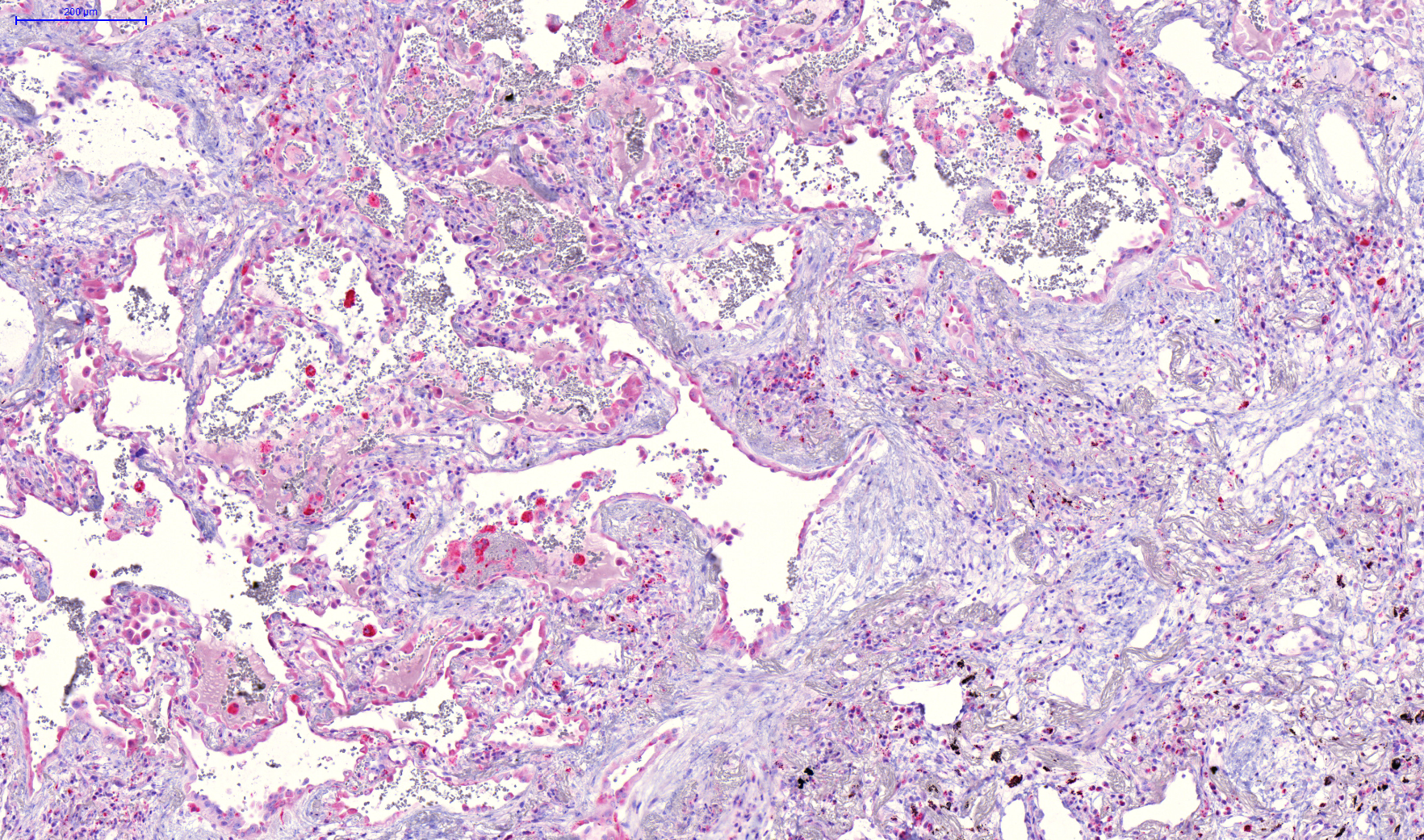

Supplement: Supplementary file 16 — Source Data for Figure 9 [file EMBJ-42-e110597-s009.zip › SourceData_Figure 9C /_IHC333-3 Gies180 LMP2 x4C IPF_2_4x_Bar.tif]
