# Supplementary material for: DNA sensing via the cGAS/STING pathway activates the immunoproteasome and adaptive T‐cell immunity
Source: EMBO J. 2023 Mar 13;42(8):e110597. doi: 10.15252/embj.2022110597 (PMC10106989; doi:10.15252/embj.2022110597)
Supplement: Supplementary file 17 [file EMBJ-42-e110597-s017.pdf]

THE EMBO JOURNAL

DNA sensing via the cGAS/STING pathway activates the immunoproteasome and adaptive T-cell immunity

Xinyuan Wang, Huabin Zhang, Yuqin Wang, Laylan Bramasole, Kai Guo, Fatima Mourtada, Thomas Meul, Qianjiang Hu, Valeria Viteri, Ilona Kammerl, Melanie Koenigshoff, Mareike Lehmann, Thomas Magg, Fabian Hauck, Isis Fernandez, and Silke Meiners
DOI: 10.15252/embj.2022110597

Corresponding author(s): Silke Meiners (smeiners@fz-borstel.de)

Review Timeline:

Submission Date:	4th Jan 22
Editorial Decision:	11th Feb 22
Revision Received:	30th Dec 22
Editorial Decision:	26th Jan 23
Revision Received:	9th Feb 23
Accepted:	20th Feb 23

Editor: Karin Dumstrei

Transaction Report:

(Note: With the exception of the correction of typographical or spelling errors that could be a source of ambiguity, letters and reports are not edited. Depending on transfer agreements, referee reports obtained elsewhere may or may not be included in this compilation. Referee reports are anonymous unless the Referee chooses to sign their reports.)

Dear Prof. Meiners,

Thank you for submitting your manuscript to The EMBO Journal. Your study has now been seen by three referees and their comments are provided below.

As you can see below, the referees appreciate the insights reported but also find that further analysis is needed for consideration here. As you can see better support for that it is mtDNA that drives the cGAS-response and the contributions of IFN is needed. Should you be able to address the raised concerns in full, then I would like to invite you to submit a revised version.

I think it would be helpful to discuss the revisions further and we can do so via video call or email. Whatever works best for you

When preparing your letter of response to the referees' comments, please bear in mind that this will form part of the Review Process File, and will therefore be available online to the community. For more details on our Transparent Editorial Process, please visit our website: https://www.embopress.org/page/journal/14602075/authorguide#transparentprocess

I thank you for the opportunity to consider your work for publication. I look forward to discussing your revisions further.

Yours sincerely,

Karin Dumstrei, PhD
Senior Editor
The EMBO Journal

Instructions for preparing your revised manuscript:

I have attached a guide with helpful tips on how to prepare the revised version.

Guide For Authors: https://www.embopress.org/page/journal/14602075/authorguide

We realize that it is difficult to revise to a specific deadline. In the interest of protecting the conceptual advance provided by the work, we recommend a revision within 3 months (12th May 2022). Please discuss the revision progress ahead of this time with the editor if you require more time to complete the revisions. Use the link below to submit your revision:

https://emboj.msubmit.net/cgi-bin/main.plex

Referee #1:

This study by Wang and colleagues implicates mtDNA stress in the regulation of adaptive immunity. Specifically, the authors propose (from an extensive dataset) that cGAS-STING mediated upregulation of immunoproteasome function (by mtDNA) promotes T-cell activation, and aberrant activation of this process may underlie pathology of idiopathic pulmonary fibrosis. The research is timely, and well carried out/presented. Overall, the data are largely supportive of the authors' model. Nonetheless, I have some concerns that should be addressed below.

- The authors show in various expts. that mtDNA is sufficient to trigger the effects they see (e.g. upregulation of immunoproteasome activity, CD8 T-cell activation etc). Given that the effects are cGAS-STING dependent, its not surprising that transfection of mtDNA has these effects (transfection of any DNA source might be expected to be the same - though this control is not done in these expts.), what is missing is the demonstration that mtDNA is required for the effects the authors observe - this should be investigated, for instance, does loss of mtDNA in polgAmut/mut cells revert the induction of immunoproteasome components seen in Fig 1a-c, and prevent constitutive activation of cGAS-STING in these cells ? - generation of mtDNA deficient (Rho zero) cells is a relatively straight forward approach to investigate this question.

- the demonstration that T-cell stimulatory effects are dependent on immunoproteasome upregulation are reliant upon a single inhibitor (Figure 5), given the central message of the paper centers on immunoproteasome upregulation being relevant for these effects, the authors should address this in orthogonal manner (for instance through shRNA suppression of immunoproteasome sub-units)

- the title suggests a generality of the findings (i.e. mtDNA stress) extending beyond PolgAmut/mut cells, however only one

experiment is done using a different model of mtDNA stress (ND5 patient cells), figure 1 - it would be supportive of a general relevance of the authors' findings to investigate if these cells also upregulate LMP2/7 in a cGAS-STING, mtDNA dependent manner.

Referee #2:

In this manuscript, Wang et al. determine the effects of mitochondrial stress on MHC I antigen processing and presentation. Using primary mouse and human fibroblasts with mutations in their mtDNA, the authors convincingly show that mitochondrial stress (mtDNA in the cytosol) activates the cGAS/STING pathway, leading to type I interferon production. The type I interferon IFNbeta in turn induces the formation of immunoproteasomes and the expression of other components of the MHC I antigen processing pathway, resulting in enhanced MHC I antigen presentation. This is illustrated by the improved T cell-mediated detection of the H-Y model antigen on primary mouse, male fibroblasts. Similar results are obtained using primary mouse alveolar type 2 epithelial cells (AT2) and human lung fibroblasts, transfected with mtDNA. The authors then continue to analyze single cell sequencing data from a public database, and show the specific activation of the type I IFN response in AT2 cells of patients with idiopathic pulmonary fibrosis (IPF). In addition, analysis of CD8 T cell gene signatures indicates their activation, but also exhaustion. Staining of lung tissue of IPF patients confirms the formation of immunoproteasomes and infiltration of PD-1+ CD8 T cells in the lungs of IPF patients.

Overall, this is a very nice analysis of the effects of mitochondrial dysfunction on antigen processing and T cell activation. While the single steps in this process (effects of mt stress and cytosolic mtDNA leakage on cGAS/STING activation and IFNbeta production, and exposure of cells to interferons on antigen processing) have been described, the linkage of these different steps and their potential role in lung fibrotic disease is entirely new. All analyses are well-performed, include many different controls and exploit different primary cells of both mice and humans. This is very convincing.

A few concerns:
1. Although the analysis of IPF patients is an excellent turn, a T cell and autoimmune-driven pathomechanism for IPF seems just one possibility. Chronic inflammation may also, secondarily, activate CD8 T cell responses.
2. Are observed CD8 T cell responses auto-immune responses? And does mitochondrial stress prime these responses or aggravate the disease phenotype?
3. The authors refer to a well-established role of mitochondrial dysfuntion and mtDNA stress in IPF patients (p. 14). Are there any mtDNA mutations associated with IPF? In general or in the analysed cohorts? Is IPF inherited, from mother to child?
4. p. 9 title: This paragraph describes the presentation of a model antigen to reporter T cells, not the activation of autoreactive T cells.

Minor concern:

Fig 5 c and d are exchanged
-

Referee #3:

Wang et al. investigate the effects of a defect in the mitochondrial DNA-polymerase gamma and arrive at a model where this defect, through generating 'mitochondrial stress', causes the cytosolic presence of mtDNA and the stimulation of cGAS with an associated IFN I-signature. Enhanced expression and function of the immunoproteasome are further found, and enhanced numbers of CD8 T cells, as well as signs of T cell exhaustion, are described in vivo.

These are interesting results, and the authors present an intriguing model. There are however a number of points that in my view still require attention:

1. The conclusion that it is mtDNA that drives the cGAS-response and the downstream effects is in my view insufficiently supported. Despite some issues with data presentation (below) and individual findings, it is convincing that cGAS is activated. However, the evidence that this is the result of mtDNA-release seems inconclusive. Why mitochondrial stress (whatever that means: please discuss why the accumulation of mutations causes stress) should cause the release of mtDNA is at least not obvious. Some increase of cytosolic mtDNA is measured, but it is impossible to tell how much that is because only a relative measure is given (wt vs. mut cells). It is further easily conceivable that mut-cells have higher levels of apoptosis and disintegrating mitochondria, and that this is what is measured (in which case the cell would be dead and presumably not contribute to the response). I appreciate the difficulties in establishing mtDNA-release, but as the data stand this is inconclusive; transfecting mtDNA does not show that mtDNA is responsible. How would the release work? Do mitochondria disintegrate? Release of mtDNA has only been convincingly shown for late stage apoptosis. If changes to mitochondrial integrity are suggested to be the connection between the mutation and the release of mtDNA, this has to be tested by high-resolution

microscopy (it can also be attempted to get rid of mtDNA). Cytosolic mtDNA has to be quantified in absolute terms (copy numbers detectable per cell). At the same time, the authors do not look past mtDNA. There is substantial evidence that cGAS/STING can be activated by micronuclei or perhaps chromosomal misalignments/chromatin bridges. It has to be tested whether such potential ligands (which do exist in non-malignant cells) are different between the cell lines. To my mind, this is a critical point that requires more work.

2. Although there is clear evidence of cGAS-activation and a role of this pathway, the evidence for a contribution of IFN is not strong. Core components like pIRF3 are in fact down-regulated (Fig. S1), the relative phosphorylation of STAT1 is probably less (Fig. 2), and the secretion of IFN- is minute (from 1 to under 2.5 pg/ml). Again, the finding that mtDNA and IFN upregulate the immunoproteasome (the phenotype of the mut-cells) is not unequivocal evidence that the mutation upregulates the immunoproteasome through mtDNA and IFN. The concentrations of IFN used in these experiments are also presumably much higher than what is secreted (the authors give only IU for the stimulation and weight/vol. for the secretion; the 100 IU/ml used is probably about 5-10,000 fold what the cells secrete (1-2 pg/ml; please convert the IU). The role of IFN could be tested by KD of the receptor. This is perhaps not necessary here but on the basis of the data presented the interpretation of the role of IFN has to be toned down.

3. Related to the last point, the presentation of the stimulatory signature as an IFN-signature seems biased. The text says there is increased phosphorylation of TBK1, and the Western blot in the paper supports this. Quantification of three blots in Fig. S1 however shows substantial variation and basically no difference (apparently up-regulation in only one of three experiments). It is inappropriate to show one positive experiment in the main figure while moving the two negative experiments without further mention to the supplement.

4. The text states that LMP7/beta5-activity is found in mutant MEFs but I cannot see this in the data (Fig. 1c). Please clarify.

5. It is certainly not without interest that IPF-patients have an IFN-signature but this does not seem to show that the mtDNA-cGAS-pathway operates.

6. I fear technical replicates are not up to the required standard. Fig. S2 and Fig. 4A give results from one experiment, and this is not enough. At least some critical aspects have to be confirmed in additional experiments.

7. It is unfortunate that the FACS-blots in Fig. 4c and d use such apparently different settings, or experimental conditions. As the data stand, the effect of cGAS-KD seems rather minor, but this is not really clear because of the different baseline.

Minor:
How stable is the phenotype of the MEFs (Fig. 1)? How old are they/after how many passages were the cells analyzed?

For some reason I have been unable to obtain access to the proteome data set. Please check this. It is not clear from the wording whether the previously obtained data set (Meul et al.) was used or new data were generated.

The legends of Fig. 5c and 5d are swapped.

Fig. 7b: are the results of mtDNA and mtDNA-DNAse statistically significantly different?

Response to the reviewers on the manuscript EMBO Journal

Referee #1:

This study by Wang and colleagues implicates mtDNA stress in the regulation of adaptive immunity. Specifically, the authors propose (from an extensive dataset) that cGAS-STING mediated upregulation of immunoproteasome function (by mtDNA) promotes T-cell activation, and aberrant activation of this process may underlie pathology of idiopathic pulmonary fibrosis. The research is timely, and well carried out/presented. Overall, the data are largely supportive of the authors' model. Nonetheless, I have some concerns that should be addressed below.

1. The authors show in various expts. that mtDNA is sufficient to trigger the effects they see (e.g. upregulation of immunoproteasome activity, CD8 T-cell activation etc). Given that the effects are cGAS-STING dependent, its not surprising that transfection of mtDNA has these effects (transfection of any DNA source might be expected to be the same - though this control is not done in these expts.), what is missing is the demonstration that mtDNA is required for the effects the authors observe - this should be investigated, for instance, does loss of mtDNA in polgAmut/mut cells revert the induction of immunoproteasome components seen in Fig 1a-c, and prevent constitutive activation of cGAS-STING in these cells ? - generation of mtDNA deficient (Rho zero) cells is a relatively straight forward approach to investigate this question. (ethidium bromide)

R1: *We thank the reviewer for his/her positive evaluation of our manuscript and fully agree with the critique that our data do not show that mtDNA is required for the induction of the immunoproteasome. According to the reviewer's suggestion, we performed mtDNA depletion experiments to investigate the loss of mtDNA on immunoproteasome induction in PolgA$^{mut/mut}$ cells. For that we followed a publication by Fernandez-Moreno et al.* (Fernández-Moreno *et al*, 2016)*, who established a protocol for generating Rho-0 cells using Stavudine as a safer option than EtBr. Of note, depletion of mtDNA in PolgA$^{mut/mut}$ mouse embryonic fibroblasts (MEF) severely reduced growth of the cells over the course of the mtDNA depletion treatment even at low doses while wildtype cells continued to grow (Figure 1 for the reviewer). We were thus unable to obtain enough viable mtDNA-depleted PolgA$^{mut/mut}$ cells to test the hypothesis that mtDNA is required for the activation of the immunoproteasome. Figure 1 for the reviewer provides the cell population doubling times for PolgA$^{mut/mut}$ and PolgA$^{wt/wt}$ cells using increasing doses of Stavudine as well as testing for the potential protective effects of uridine as suggested by Fernandez-Moreno et. al. We have mentioned our mtDNA depletion experiments in our revised results part and have toned down our statement on the essential role of mtDNA in immunoproteasome activation.*

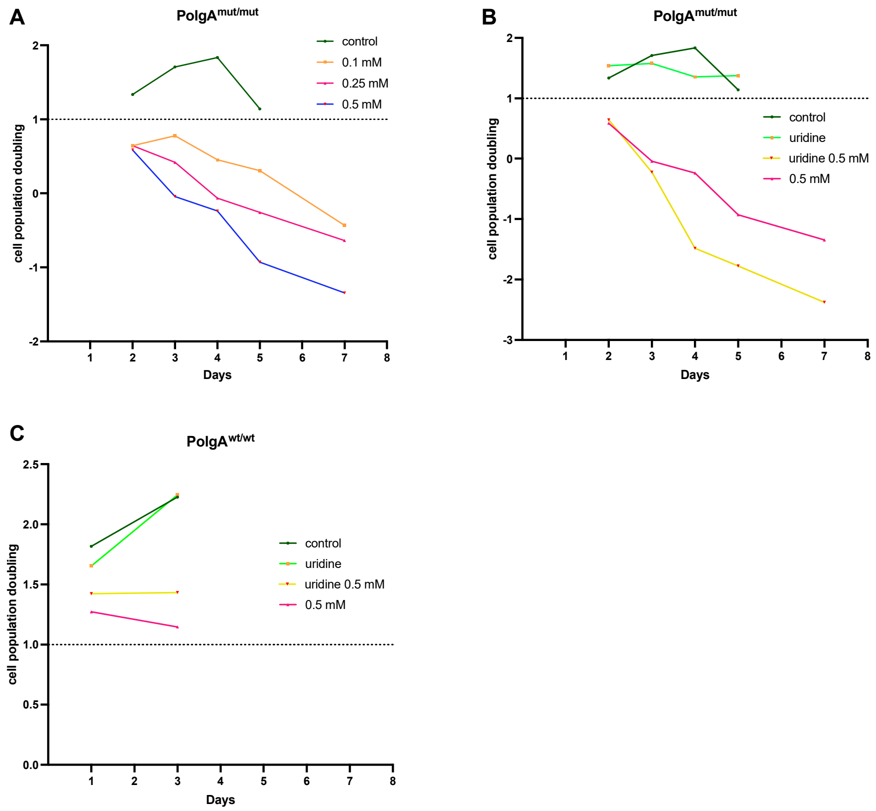

Figure 1 for the Reviewer: *mtDNA depletion in PolgA$^{mut/mut}$ and PolgA$^{wt/wt}$ cells.* ***A****) mtDNA depletion of PolgA$^{mut/mut}$ cells. PolgA$^{mut/mut}$ cells were seeded into 6-well plates at 0.1 million cells/well numbers at day 1 and then treated with different concentrations of Stavudine (D4T, D1413-50MG, Sigma) for several days. Media was changed every other day, and cells were harvested and counted every day for 7 days. (****B****) The effect of uridine was determined by cotreatment of 0.5 mM D4T with 50 mg/ml uridine (U3003, Sigma). (****C****) PolgA$^{wt/wt}$ cells were similarly treated but counted only for three days due to their continuous proliferation. Population doubling time was calculated as described in Meul et al* (Meul *et al*, 2020).

While we cannot prove that mtDNA is required for the induction of the immunoproteasome, our data clearly show that it is sufficient. We also observed cGAS-dependent induction of the immunoproteasome subunit LMP2 by siRNA-mediated partial depletion of TFAM, which causes mtDNA instability, its release into the cytosol, and activation of the cGAS-STING pathway (Bryant *et al*, 2022)*, thereby demonstrating Immunoproteasome activation in a second model of mitochondrial dysfunction. We have included these new data as Figs. 3D, EV3D and EV3E in the revised manuscript.*

According to the reviewer's suggestion, we also performed additional experiments and tested whether double-stranded (ds) DNA activates the immunoproteasome. Indeed, genomic and plasmid DNA both induce the immunoproteasome in a cGAS- and STING-dependent manner (see ***R9*** *for details and Figure 8 of the revised manuscript).*

2. The demonstration that T-cell stimulatory effects are dependent on immunoproteasome upregulation are reliant upon a single inhibitor (Figure 5), given the central message of the

paper centers on immunoproteasome upregulation being relevant for these effects, the authors should address this in orthogonal manner (for instance through shRNA suppression of immunoproteasome sub-units)

R2: *We thank the reviewer for this suggestion and have accordingly performed silencing experiments in PolgA$^{mut/mut}$ cells in order to test the effects on CD8$^+$ T cell activation. We were, however, unable to achieve efficient reduction of immunoproteasome levels in PolgA$^{mut/mut}$ cells upon silencing with LMP7-specific siRNAs as determined by Western blot analysis. This is most probably due to the long half-life of the assembled proteasome complexes which has been estimated between 7 and 12 days* (Khan *et al*, 2001)*. We thus chose to use splenocytes isolated from male C57BL/6 LMP7 Knockout (KO) mice and wildtype controls and transfected them with mtDNA to determine activation of UTY-specific CD8$^+$ T cells. As shown in the revised Figure 5E, wildtype splenocytes responded to mtDNA transfection with a significant activation of CD8$^+$ T cells which was lower in LMP7 KO cells did not. Upon genomic DNA transfection activation of CD8$^+$ T cells was abrogated in LMP7 KO cells. These data thus confirm the crucial role of the immunoproteasome for CD8$^+$ T cell activation in response to intracellular double-stranded DNA sensing. We included these new data into the revised manuscript as revised Figure 5E and 8B.*

3. The title suggests a generality of the findings (i.e. mtDNA stress) extending beyond PolgAmut/mut cells, however only one experiment is done using a different model of mtDNA stress (ND5 patient cells), figure 1 - it would be supportive of a general relevance of the authors' findings to investigate if these cells also upregulate LMP2/7 in a cGAS-STING, mtDNA dependent manner.

R3: *We fully agree with the reviewer's comment to confirm the general relevance of our findings in the human ND5 cells. In accordance with his/her suggestion, we performed new experiments in these cells and tested the effects of cGAS silencing on immunoproteasome activation. We noted, however, that the baseline expression of the immunoproteasome in the newly defrosted ND5 cells were lower at baseline compared to the non-diseased skin fibroblast control (NDSF). We thus tested several frozen vials and passage numbers of the ND5 cells but were unable to obtain data consistent with our previous findings. As we were unable to reproduce our findings on immunoproteasome upregulation in ND5 cells compared to healthy donors and cannot rule out that the previously observed induction of the immunoproteasome in these cells was related to some unknown cell culture conditions, we would like to omit these data from our revised manuscript.*

To confirm our findings in a different model of mtDNA stress, we partially depleted TFAM in wildtype and cGAS KO embryonic fibroblasts and analyzed immunoproteasome induction as already mentioned in response 1. In accordance with our data from the PolgA$^{mut/mut}$ cells, we observed cGAS-dependent induction of the immunoproteasome subunits LMP2 and LMP7 in wildtype and cGAS KO cells. SiRNA-mediated depletion of TFAM is well known to induce mtDNA instability, its release into the cytosol, and activation of the cGAS-STING pathway

(Bryant *et al*, 2022)*. We have included these new data as Figs. 3D, EV3D and EV3E into the revised manuscript.*

Moreover, to prove a general relevance of our findings we tested whether induction of the immunoproteasome is restricted to mtDNA by transfecting dsDNA, such as herring testes or plasmid DNA, into mouse embryonic fibroblasts. We also analyzed the effects of mtDNA and dsDNA transfection in human skin fibroblasts and compared immunoproteasome activation in neonatal controls with primary cells from a newborn patient with a homozygous STAT1 loss-of-function (LOF) mutation and the heterozygous adult fibroblasts of their parents(revised Figs. 6I, EV5E and 8C). Together with additional experiments using LMP7, cGAS and STING KO mouse cells, our experiments confirm that the immunoproteasome is activated by dsDNA in a cGAS/STING/STAT1/immunoproteasome-dependent manner in both human and mouse cells. We included these data into the revised manuscript as a new Figure 8. These experiments demonstrate cGAS/STING-mediated immunoproteasome activation as a general new concept that has not been described before and which has profound consequences for the activation of adaptive immunity. We have accordingly changed the title to „DNA sensing via the cGAS/STING pathway activates the immunoproteasome and adaptive T-cell immunity".

Referee #2:

In this manuscript, Wang et al. determine the effects of mitochondrial stress on MHC I antigen processing and presentation. Using primary mouse and human fibroblasts with mutations in their mtDNA, the authors convincingly show that mitochondrial stress (mtDNA in the cytosol) activates the cGAS/STING pathway, leading to type I interferon production. The type I interferon IFNbeta in turn induces the formation of immunoproteasomes and the expression of other components of the MHC I antigen processing pathway, resulting in enhanced MHC I antigen presentation. This is illustrated by the improved T cell-mediated detection of the H-Y model antigen on primary mouse, male fibroblasts. Similar results are obtained using primary mouse alveolar type 2 epithelial cells (AT2) and human lung fibroblasts, transfected with mtDNA. The authors then continue to analyze single cell sequencing data from a public database, and show the specific activation of the type I IFN response in AT2 cells of patients with idiopathic pulmonary fibrosis (IPF). In addition, analysis of CD8 T cell gene signatures indicates their activation, but also exhaustion. Staining of lung tissue of IPF patients confirms the formation of immunoproteasomes and infiltration of PD-1+ CD8 T cells in the lungs of IPF patients.

Overall, this is a very nice analysis of the effects of mitochondrial dysfunction on antigen processing and T cell activation. While the single steps in this process (effects of mt stress and cytosolic mtDNA leakage on cGAS/STING activation and IFNbeta production, and exposure of cells to interferons on antigen processing) have been described, the linkage of these different steps and their potential role in lung fibrotic disease is entirely new. All

analyses are well-performed, include many different controls and exploit different primary cells of both mice and humans. This is very convincing. A few concerns:

4. Although the analysis of IPF patients is an excellent turn, a T cell and autoimmune-driven pathomechanism for IPF seems just one possibility. Chronic inflammation may also, secondarily, activate CD8 T cell responses. Are observed CD8 T cell responses auto-immune responses? And does mitochondrial stress prime these responses or aggravate the disease phenotype?

R4: *We thank the reviewer for his/her positive evaluation of our manuscript and the excellent questions, which, however, we can only speculate on. It is indeed well known that a chronic cytokine milieu facilitates the expansion of CD8$^+$ T cells, their contraction as well as memory T cell formation* (Haring *et al*, 2006). *Initial priming, however, always involves T cell receptor (TCR) activation by MHC class I antigen presentation and co-stimulation. Our data extracted from the IPF single cell atlas clearly (revised Figure 9B) indicate that the CD8$^+$ T cells are activated, which is supported by the overlapping staining with PD-1, an exhaustion marker that is only turned on upon T cell activation as also recently demonstrated by Sumida et al.* (Sumida *et al*, 2022). *This thus suggests that the T cells at some point of IPF pathogenesis were exposed to antigen/TCR interaction (signal 1), co-stimulation (signal 2) and inflammatory cytokines (signal 3). While we do not know the antigenic specificity of the CD8$^+$ T cells in the IPF tissue, antigen recognition is essential for CD8$^+$ T cell activation. Potential antigenic sources can be viral antigens as observed in chronic infections* (McKinney *et al*, 2015). *As IPF is typically not regarded as a disease of chronic infection, CD8$^+$ T cell activation might rather be caused by molecular mimicry. This concept describes the cross-reactivity of anti-viral cytotoxic CD8$^+$ T cells to autoantigens and environmental bacterial antigens (Oldstone, 1998). The cytotoxic response to these antigens is much weaker compared to the initial viral antigens but causes a persistent low-level activation of autoreactive CD8$^+$ T-cells* (Misko *et al*, 1999) *as for example shown for Psoriasis, type I diabetes and recently for multiple sclerosis and age-dependent neuronal degeneration* (Valdimarsson *et al*, 2009; Girdhar *et al*, 2022; Campisi *et al*, 2022; Zhou *et al*, 2022). *A chronic inflammatory milieu such as in IPF lungs will most probably contribute to expansion of reactive T cell clones and activation of their effector functions. Mitochondrial or genomic DNA stress as well as any other type of stress in lung cells will further aggravate the proinflammatory milieu upon local release of type I interferons and stress-related cytokines such as IL-6, TNFα, or IL-1β by stressed cells. Our extracted scRNA seq data clearly indicated elevated levels of type I interferon signaling in aberrant alveolar epithelial cells of IPF lungs which supports this notion. To further examine the effector function of the CD8$^+$ T cell population present in IPF tissue, we analyzed the gene expression signature for the different effector subtypes. Specifically, we looked for the T cell subsets Tc1 (CTLs), Tc2, Tc9, Tc17, Tc22, and immunosuppressant Tregs in the scRNA seq dataset extracted from the IPF atlas (Figure 2 for*

the reviewer). CD8$^+$ CD3$^+$ T cells were clustered into 7 cell populations. The preliminary analysis suggests an enrichment for the cytotoxic Tc1 population in IPF lungs.

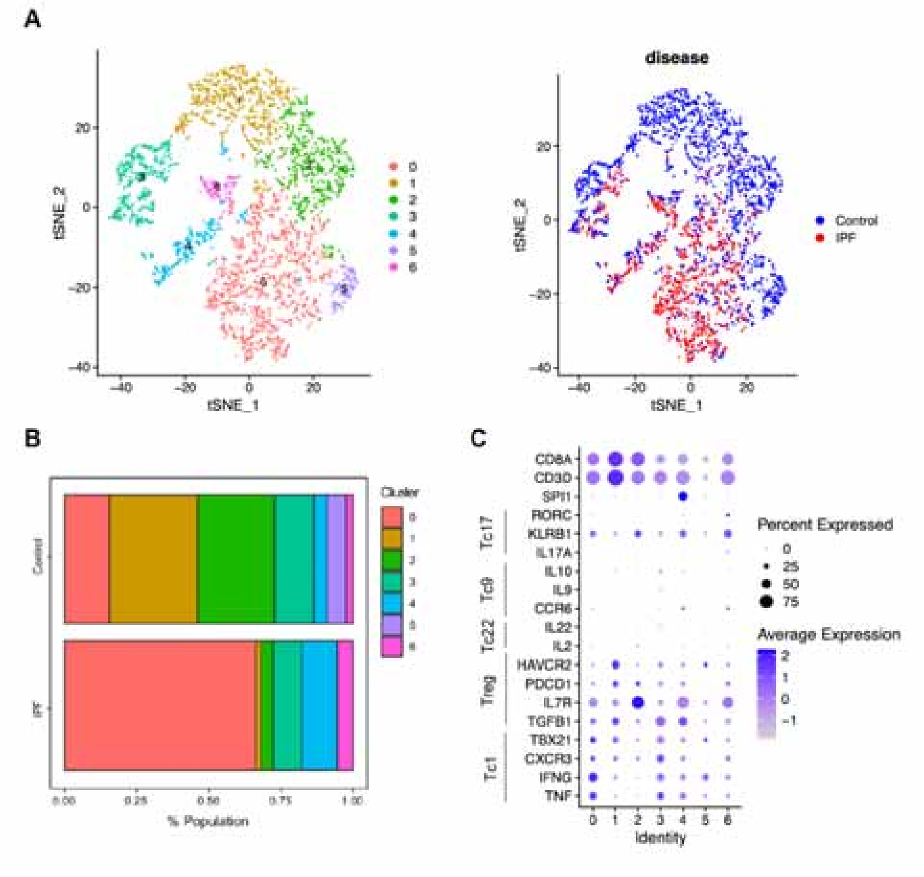

Figure 2 for the reviewer: *Dissecting the effector CD8$^+$ T cell populations in IPF lungs. (**A**) t-SNE analysis of single cells from CD3$^+$CD8$^+$ T cells using the publicly available data set GSE135893. In the left t-SNE map, 7 cell-type clusters are labeled with different colors, in the right map, the clusters are labeled according to cells derived from IPF or donor lungs. (**B**) The bar chart depicts the percentage of cluster populations in IPF and donor lungs. (**C**) DotPlot analysis shows the differentiation markers of CD8$^+$ T cells in the 7 clusters from the t-SNE analysis.*

However, these data should be considered with caution as only very few marker genes were used to define the different T cell populations. We have thus not included them into our revised manuscript but extended our discussion on the concept of autoimmune CD8$^+$ T cells in the pathogenesis of IPF.

5. The authors refer to a well-established role of mitochondrial dysfunction and mtDNA stress in IPF patients (p. 14). Are there any mtDNA mutations associated with IPF? In general or in the analysed cohorts? Is IPF inherited, from mother to child?

***R5**: We thank the reviewer for this important comment. While there is strong evidence for mitochondrial dysfunction in the pathogenesis of IPF (e.g. summarized in* (Bueno *et al*, 2020)*, there is no evidence for lung involvement in primary hereditary mitochondrial DNA-related diseases* (Schon *et al*, 2012)*. In accordance, the here used PolgA$^{mut/mut}$ mice do not develop any lung dysfunction but die due to premature aging and organ failure* (Trifunovic *et al*, 2004)*. We would like to emphasize, however, that mtDNA stress is just one of several ways to induce the adaptive cGAS/STING-mediated type I interferon signaling that we observed here (see revised discussion). In our revised manuscript, we have extended our initial finding on mtDNA to genomic DNA demonstrating a general relevance of DNA-induced immunoproteasome activation. In line with our findings are recent data on silica- and SARS-CoV2-induced lung damage that reported on the key role of the cGAS/STING/type I interferon signaling axis* (Benmerzoug *et al*, 2018; Domizio *et al*, 2022; Sumida *et al*, 2022)*. Of note, activation of cGAS/STING signaling has recently been demonstrated for dysfunctional telomeres* (Abdisalaam *et al*, 2020) *and might link telomere dysfunction, as found in sporadic and hereditary IPF, to development of pulmonary fibrosis. We have included these aspects into our revised discussion.*

6. p. 9 title: This paragraph describes the presentation of a model antigen to reporter T cells, not the activation of autoreactive T cells.

***R6:** We thank the reviewer for this comment. In case of the UTY CD8$^+$ T cell assay, we indeed assay the response of a reporter T cell clone to an endogenous antigen, i.e., the UTY$_{264-254}$ antigen. The response can thus be described as an autoreactive T cell response. This is not true for our second CD8$^+$ T cell assay that utilized a T cell reporter clone specific for an influenza A virus M1 antigen. We have thus corrected our wording for this assay as suggested.*

7. Fig 5 c and d are exchanged

***R7:** We have corrected the figure.*

Referee #3:

Wang et al. investigate the effects of a defect in the mitochondrial DNA-polymerase gamma and arrive at a model where this defect, through generating 'mitochondrial stress', causes the cytosolic presence of mtDNA and the stimulation of cGAS with an associated IFN I-signature. Enhanced expression and function of the immunoproteasome are further found, and enhanced numbers of CD8 T cells, as well as signs of T cell exhaustion, are described in vivo. These are interesting results, and the authors present an intriguing model. There are however a number of points that in my view still require attention:

8. The conclusion that it is mtDNA that drives the cGAS-response and the downstream

effects is in my view insufficiently supported. Despite some issues with data presentation (below) and individual findings, it is convincing that cGAS is activated. However, the evidence that this is the result of mtDNA-release seems inconclusive. Why mitochondrial stress (whatever that means: please discuss why the accumulation of mutations causes stress) should cause the release of mtDNA is at least not obvious. Some increase of cytosolic mtDNA is measured, but it is impossible to tell how much that is because only a relative measure is given (wt vs. mut cells). It is further easily conceivable that mut-cells have higher levels of apoptosis and disintegrating mitochondria, and that this is what is measured (in which case the cell would be dead and presumably not contribute to the response). I appreciate the difficulties in establishing mtDNA-release, but as the data stand this is inconclusive; transfecting mtDNA does not show that mtDNA is responsible. How would the release work? Do mitochondria disintegrate? Release of mtDNA has only been convincingly shown for late stage apoptosis. If changes to mitochondrial integrity are suggested to be the connection between the mutation and the release of mtDNA, this has to be tested by high-resolution microscopy (it can also be attempted to get rid of mtDNA). Cytosolic mtDNA has to be quantified in absolute terms (copy numbers detectable per cell).

R8: *We thank the reviewer for his/her important comments and fully agree that the viability of the mutator cells is key for any experiments on mtDNA stress. In our previous study* (Meul *et al*, 2020)*, where we used the PolgA$^{mut/mut}$ cells for the first time in our lab, we extensively characterized these cells, For the reviewer's convenience, we have summarized these data in a figure for the reviewer (Figure 3 for the reviewer):*

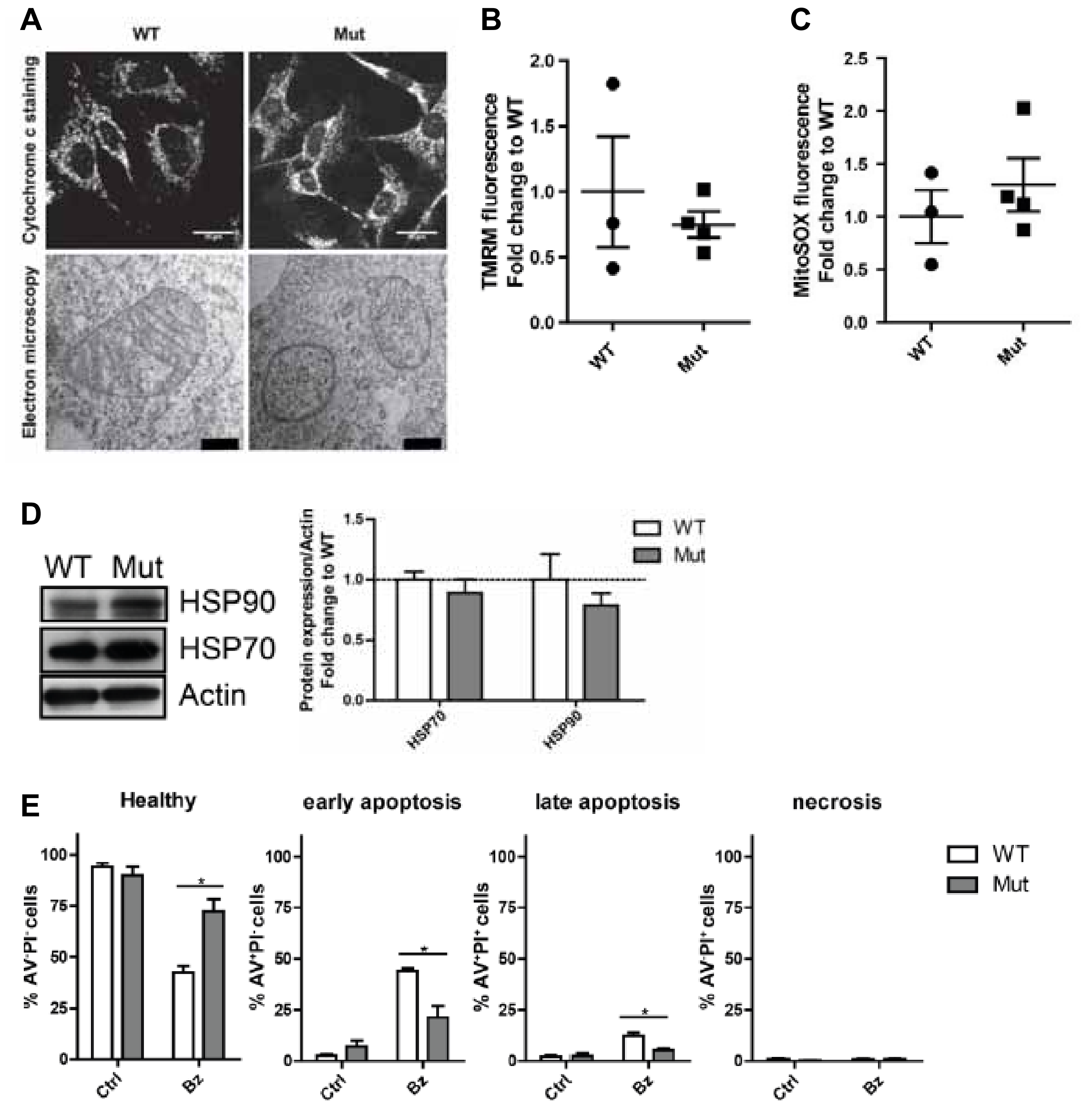

Figure 3 for the reviewer: *PolgA$^{mut/mut}$ cells are fully viable and not overly stressed.* ***A)*** *Representative images of the mitochondrial network in WT (n=3) and Mut (n=4) cells visualized by staining with anti-cytochrome c antibody (upper panel). Scale bar: 25 mm. Lower panel shows representative electron microscopy images of mitochondria in a single WT and Polg$^{mut/mut}$ (Mut) cell line. Scale bar: 1 mm. (Figure 2A from Meul et al.).* ***(B)*** *Mean fluorescence intensity of WT (n=3) and Mut (n=4) MEFs stained with TMRM for assessment of mitochondrial membrane potential measured by flow cytometry analysis.* ***(C)*** *MitoSOX Red for detection of mitochondrial superoxide generation, n=3(WT)-4(Mut)±SEM.* ***(D)****. Western blot analysis of HSP70 and HSP90 expression in WT (n=3) and Mut (n=4) MEFs. Actin was used as a loading control.* ***(E)*** *Quantification of healthy (Annexin V-/PI-), early apoptotic (Annexin V+/PI-), late apoptotic (Annexin V+/PI+), or necrotic (Annexin V-/PI+) cells after 24 h of 25 nM Bortezomib (Bz) or control treatment in WT (n=3) and Mut (n=4) MEFs. Bar graphs show mean+SEM. Significance was determined using student's t-test. *:p< 0.05.*

PolgA$^{mut/mut}$ cells form an intact mitochondrial network as shown by cytochrome C staining with intact mitochondria as evident in electron micrographs (3A). We also confirmed the presence of an intact mitochondrial potential using TMRM staining (3B) and absence of

stress signaling by analyzing MitoSox (3C) and monitoring hsp70 and hsp90 expression (3D). In our previous study, we had also demonstrated that the PolgA$^{mut/mut}$ cells were more resistant towards treatment with the proteasome inhibitor Bortezomib, confirming full viability of the PolgA$^{mut/mut}$ cells by FACS-based analysis of Annexin V (AV) and propidium iodide (PI) staining (3E). From these data we can thus conclude that PolgA$^{mut/mut}$ cells are fully viable, not apoptotic and that their mitochondrial structure and network are not grossly disrupted.

Regarding the reviewer's question how the mtDNA release might work, we can only speculate. It has been shown previously, that the accumulation of mutations in mitochondrial genes result in the expression of mutant mitochondrially encoded proteins which fail to assemble into the larger respiratory chain complexes I and III and IV (Edgar *et al*, 2009). *Accumulation of misfolded mitochondrial proteins has previously shown to result in the activation of mitochondrial unfolded protein response as part of an adaptive stress response* (Münch & Harper, 2016). *As mitochondria remain intact in PolgA$^{mut/mut}$ cells, this rules out release of mtDNA via the previously described BAK/BAX macropores* (McArthur *et al*, 2018). *A very recent study demonstrated release of oxidized mtDNA via mitochondrial permeability transition pores (mPTP) and VDAC channels and subsequent activation of the STING pathway* (Xian *et al*, 2022) *while the cell remains intact. Mitochondrial DNA release has also been demonstrated to take place via mPTP to activate cGAS/STING in ALS* (Yu *et al*, 2020). *Moreover, mitochondrial DNA release and activation of cGAS/STING signaling has been shown as a consequence of mitochondrial instability upon TFAM knockout or silencing but the mechanism was not further resolved* (Bryant *et al*, 2022; West *et al*, 2015). *To confirm our data obtained from the PolgA$^{mut/mut}$ cells, we performed additional experiments and partially silenced TFAM in wildtype and cGAS KO MEFs. We confirmed cGAS-dependent induction of the immunoproteasome and included these new data as Figs. 3D, EV3D and EV3E in the revised manuscript.*

According to the reviewer's suggestion, we also tried to deplete PolgA$^{mut/mut}$ cells from their mitochondrial DNA. This, however, severely affected growth of the cells and we were unable to obtain viable, mtDNA-depleted PolgA$^{mut/mut}$ cells as outlined in more detail in response ***R1*** *to reviewer 1. We confirmed, however, the presence of increased levels of mitochondrial DNA in the cytosol using a combination of cytosolic fractionation and PCR-based absolute amplification as suggested by the reviewer. These data are now included in the revised manuscript as revised Figure 2B and unambiguously demonstrate elevated levels of mtDNA in PolgA$^{mut/mut}$ cells.*

9. At the same time, the authors do not look past mtDNA. There is substantial evidence that cGAS/STING can be activated by micronuclei or perhaps chromosomal misalignments/chromatin bridges. It has to be tested whether such potential ligands (which

do exist in non-malignant cells) are different between the cell lines. To my mind, this is a critical point that requires more work.

R9: *We are grateful to the reviewer for this important comment and have accordingly performed additional experiments to analyze the potential presence of nuclear DNA. For that we performed staining for dsDNA (anti-DNA antibody), co-stained for mitochondria using an anti-hsp60 antibody and the nucleus (DAPI staining). We quantified the signal outside the nucleus and mitochondria network and observed significantly more signal for PolgA$^{mut/mut}$ cells compared to wildtype controls. These results confirm the presence of dsDNA in the cytoplasm outside the mitochondrial network and the nucleus. We have included our data as Supplementary Figure EV1C. While we cannot discriminate between mtDNA and nuclear DNA with this method, we would like to point out that PolgA$^{mut/mut}$ cells are well known for their mitochondrial DNA defect and not for nuclear DNA stress, which would eventually activate p53 and contribute to cell cycle arrest and DNA damage repair. We performed comprehensive RNA seq and proteomic analyses of the PolgA$^{mut/mut}$ cells in our previous study* (Meul *et al*, 2020) *and did not obtain any evidence for nuclear DNA damage or stress signaling. Accordingly, we assume that it is mainly mtDNA that is released from the mitochondria in the PolgA$^{mut/mut}$ cells similar to what was shown by West et al in heterozygous TFAM KO cells (Figure 1A in* (West *et al*, 2015)*.*

10. Although there is clear evidence of cGAS-activation and a role of this pathway, the evidence for a contribution of IFN is not strong. Core components like pIRF3 are in fact down-regulated (Fig. S1), the relative phosphorylation of STAT1 is probably less (Fig. 2), and the secretion of IFN-β is minute (from 1 to under 2.5 pg/ml). Again, the finding that mtDNA and IFN upregulate the immunoproteasome (the phenotype of the mut-cells) is not unequivocal evidence that the mutation upregulates the immunoproteasome through mtDNA and IFN. The concentrations of IFN used in these experiments are also presumably much higher than what is secreted (the authors give only IU for the stimulation and weight/vol. for the secretion; the 100 IU/ml used is probably about 5-10,000 fold what the cells secrete (1-2 pg/ml; please convert the IU). The role of IFN could be tested by KD of the receptor. This is perhaps not necessary here but on the basis of the data presented the interpretation of the role of IFN has to be toned down.

R10: *We thank the reviewer for his/her comments regarding the concentration of IFN-β and the role of the IFN-β signaling pathway. Regarding the concentration of IFN-β in the supernatants of PolgA$^{mut/mut}$ cells, we calculated that the 15.81 pg/ml detected by ELISA corresponds to 18.97 IU/ml recombinant mouse (rm) IFN-β according to the R&D Systems cytokine conversion table (https://www.rndsystems.com/cn/resources/technical-information/unit-conversion-table). The concentration of rmIFN-β we used for treatment was 100 IU/ml, which is thus only 5-fold higher than the concentrations of IFN-β detected in the supernatants of PolgA$^{mut/mut}$ cells. We believe that the amount of IFN-β secreted by PolgA$^{mut/mut}$ cells may thus be sufficient to induce activation of the immunoproteasome. To further corroborate the involvement of the IFN-β/STAT-1 signaling pathway in mtDNA and*

*also genomic DNA-induced activation of the immunoproteasome, we used an antagonizing antibody against Interferon Alpha And Beta Receptor Subunit 1 (IFNAR1) to block IFN-β signaling. The system was first validated by stimulating wildtype MEFS with IFN-β upon pre-treatment with different doses of the antagonizing IFNAR-1 antibody. Preincubation with the antibody effectively blocked IFN-β signaling as demonstrated by inhibition of immunoproteasome induction (revised Figure EV3C). IFNAR-1 signaling was then blocked in PolgA$^{mut/mut}$ cells using the antagonizing IFNAR antibody and effectively inhibited immunoproteasome induction (revised Figure 3C). Moreover, as outlined in response 3 to reviewer 1 (**R3**), we also used primary skin fibroblasts derived from a newborn patient with a homozygous STAT1 loss-of-function (LOF) mutation as well as cells from the heterozygous parents. In the LOF cells, mtDNA and dsDNA transfection failed to induce immunoproteasome expression. We included these data as an additional validation of the type I interferon signaling pathway in human and patient-derived cells into our revised manuscript as Figs. 6I, EV5E and 8C.*

11: Related to the last point, the presentation of the stimulatory signature as an IFN-signature seems biased. The text says there is increased phosphorylation of TBK1, and the Western blot in the paper supports this. Quantification of three blots in Fig. S1 however shows substantial variation and basically no difference (apparently up-regulation in only one of three experiments). It is inappropriate to show one positive experiment in the main figure while moving the two negative experiments without further mention to the supplement.

***R11:** We thank the reviewer for pointing out this variation in TBK1 signaling and apologize for not clearly commenting on the variation. We agree that our statement on elevated TBK1 signaling is not correct and have exchanged the blots for more representative ones as well as toned down our statement. However, we would like to point out that we used three different wildtype and three distinct PolgA$^{mut/mut}$ cell lines that had been generated as immortalized MEFs from different mice. The different experiments and the high variation thus result from the differential effects of accumulating mtDNA mutations in these three different PolgA$^{mut/mut}$ cell lines and also from clonal variation of the wild type cell lines. While this variation made it difficult for us to pick up significant differences in cGAS/STING signaling, the use of different cell lines seemed more appropriate as it reflects true biological variation. Moreover, we would like to stress that the PolgA$^{mut/mut}$ cells display chronic mitochondrial DNA stress which might result in adaptation of signaling pathways. Indeed, acute stimulation of the cGAS/STING pathway by mtDNA transfection reproducibly activated the pathway in a time-dependent manner as shown in Figures 6G and EV5D.*

12. The text states that LMP7/beta5-activity is found in mutant MEFs but I cannot see this in the data (Fig. 1c). Please clarify.

***R12:** We thank the reviewer for this careful observation. The activity-based probe MVB127 was used for the detection of LMP7 and its corresponding beta5 standard subunit as it*

specifically binds to these two active sites. The distinct resolution of these two subunits in the SDS gel and thus quantification of single active sites, however, is hampered as the mouse LMP7 and beta5 proteins are of similar molecular weight and thus do not separate in our gels. For that reason, we refer to these subunits as LMP7/beta5. The signal for this combination of subunits is not altered in PolgA$^{mut/mut}$ cells compared to wildtype as it represents the combination of both, the standard and immunosubunits. We have explained this limitation in the figure legend of the revised version of our manuscript.

13. It is certainly not without interest that IPF-patients have an IFN-signature but this does not seem to show that the mtDNA-cGAS-pathway operates.

R13: *We thank the reviewer for pointing that out and are fully aware of this limitation which we discussed in our revised manuscript. As outlined in response* ***R5*** *to reviewer 2, we believe that this IFN signature might stem from different kinds of DNA or cellular stress signaling such as telomere-related DNA stress* (Grice *et al*, 2015; Lv *et al*, 2022)*, DNA stress* (Benmerzoug *et al*, 2018) *or ER stress* (Studencka-Turski *et al*, 2019)*. Moreover, very recent data from long-COVID19 patients also support the concept of an untimely type I interferon signaling in parenchymal cells upon unresolved virus infection which potentially drives chronic immune activation and fibrosis* (Sumida *et al*, 2022; Domizio *et al*, 2022)*. In our revised manuscript, we extended our discussion with regard to a more general concept of type I IFN in fibrosis and also included recent publications on SARS-CoV2 infection.*

14. I fear technical replicates are not up to the required standard. Fig. S2 and Fig. 4A give results from one experiment, and this is not enough. At least some critical aspects have to be confirmed in additional experiments.

R14: *We thank the reviewer for this careful observation and would like to explain our experiment in more detail: RNA sequencing was performed with one wildtype and one PolgA$^{mut/mut}$ cell line instead of using three different lines as for most of the other experiments. The n of 5, however, is not a technical replicate of the same RNA but all replicates represent independent experiments, i.e., culturing and harvesting of the cells as well as separate RNA preparations and RNA sequencing of each sample. As such they represent biological and not technical replicates. The reason for this type of analysis was that we performed a parallel stimulation experiment of each wildtype and PolgA$^{mut/mut}$ cell line in 5 independent experiments unrelated to this study. We apologize for not explaining this experimental setup properly and have added more details on the replicates in the figure legend of the revised manuscript. The activation of an MHC class I signature in PolgA$^{mut/mut}$ cells was also confirmed by proteomic analysis (Figure 2A). The STAT1 signature was more clearly detected on the RNA than on the protein level probably because of deeper coverage.*

15. It is unfortunate that the FACS-blots in Fig. 4c and d use such apparently different

settings, or experimental conditions. As the data stand, the effect of cGAS-KD seems rather minor, but this is not really clear because of the different baseline.

R15: *We agree with the reviewer that the two experiments are difficult to compare. In Figure 4c, we silenced cGAS by siRNA transfection and compared it to non-sense scrambled siRNA transfection. The reason for this comparison is that the transfection procedure and introduction of any RNA might trigger a type I interferon response resulting in cGAS activation. Indeed, we observed minor upregulation of cGAS under conditions of non-sense scambled siRNA transfection compared to untransfected controls (see Western blot for cGAS in Figure EV3B) while immunoproteasome or STAT1 expression were not altered (Figure 3B). Compared to the scrambled siRNA control, silencing of cGAS decreased H2k^{b} surface expression in PolgA$^{mut/mut}$ cell lines by 50%. In Figure 4D, we used wildtype MEFs instead and transfected them with mtDNA or DNase I pre-treated mtDNA. As a control, we used cells that underwent the same transfection procedure but without adding DNA. We now corrected the labeling of the Y-axis to "fold over Ctrl" to make the different settings more clear. The baseline cannot be compared in these two experiments as we used PolgA$^{mut/mut}$ cells in Figure 4C but wildtype MEFs in Figure 4D.*

16. How stable is the phenotype of the MEFs (Fig. 1)? How old are they/after how many passages were the cells analyzed?

R16: *The MEFs were used before passage 15 and at different passage numbers. PolgA$^{mut/mut}$ cells are growing slower than wildtype MEFs* (Meul *et al*, 2020) *and this became more evident at passage numbers higher than 15. It is important to note, that all cell lines are immortalized. The phenotype of the cells regarding the activation of the immunoproteasome is very stable and the experiments were easily reproduced by different scientists in my new lab at the Research Center Borstel.*

17. For some reason I have been unable to obtain access to the proteome data set. Please check this. It is not clear from the wording whether the previously obtained data set (Meul et al.) was used or new data were generated.

R17*. We apologize for not stating clearly that the RNA seq and proteomics data were generated in the course of our previous study* (Meul *et al*, 2020). *They are already published and are all publicly available. The proteomics data set can be found under the following link:*

http://proteomecentral.proteomexchange.org/cgi/GetDataset?ID=PXD019695

18. The legends of Fig. 5c and 5d are swapped.

R18: *We thank the reviewer for pointing out this mistake and have corrected it accordingly.*

19. Fig. 7b: are the results of mtDNA and mtDNA-DNAse statistically significantly different?

R19. *We tested the significance and obtained a p value of 0.0085 using One-way ANOVA testing and added this information to the figure legend.*

References

Abdisalaam S, Bhattacharya S, Mukherjee S, Sinha D, Srinivasan K, Zhu M, Akbay EA, Sadek HA, Shay JW & Asaithamby A (2020) Dysfunctional telomeres trigger cellular senescence mediated by cyclic GMP-AMP synthase. *Journal of Biological Chemistry* 295: 11144–11160

Benmerzoug S, Rose S, Bounab B, Gosset D, Duneau L, Chenuet P, Mollet L, Le Bert M, Lambers C, Geleff S, *et al* (2018) STING-dependent sensing of self-DNA drives silica-induced lung inflammation. *Nat Commun* 9: 5226

Bryant JD, Lei Y, VanPortfliet JJ, Winters AD & West AP (2022) Assessing Mitochondrial DNA Release into the Cytosol and Subsequent Activation of Innate Immune-related Pathways in Mammalian Cells. *Current Protocols* 2

Bueno M, Calyeca J, Rojas M & Mora AL (2020) Mitochondria dysfunction and metabolic reprogramming as drivers of idiopathic pulmonary fibrosis. *Redox Biology*: 101509

Campisi L, Chizari S, Ho JSY, Gromova A, Arnold FJ, Mosca L, Mei X, Fstkchyan Y, Torre D, Beharry C, *et al* (2022) Clonally expanded CD8 T cells characterize amyotrophic lateral sclerosis-4. *Nature* 606: 945–952

Domizio JD, Gulen MF, Saidoune F, Thacker VV, Yatim A, Sharma K, Nass T, Guenova E, Schaller M, Conrad C, *et al* (2022) The cGAS–STING pathway drives type I IFN immunopathology in COVID-19. *Nature* 603: 145–151

Edgar D, Shabalina I, Camara Y, Wredenberg A, Calvaruso MA, Nijtmans L, Nedergaard J, Cannon B, Larsson N-GG & Trifunovic A (2009) Random Point Mutations with Major Effects on Protein-Coding Genes Are the Driving Force behind Premature Aging in mtDNA Mutator Mice. *Cell Metabolism* 10: 131–138

Fernández-Moreno M, Hermida-Gómez T, Gallardo ME, Dalmao-Fernández A, Rego-Pérez I, Garesse R & Blanco FJ (2016) Generating Rho-0 Cells Using Mesenchymal Stem Cell Lines. *PLoS ONE* 11: e0164199

Girdhar K, Huang Q, Chow I-T, Vatanen T, Brady C, Raisingani A, Autissier P, Atkinson MA, Kwok WW, Kahn CR, *et al* (2022) A gut microbial peptide and molecular mimicry in the pathogenesis of type 1 diabetes. *Proc Natl Acad Sci USA* 119: e2120028119

Grice GL, Lobb IT, Weekes MP, Gygi SP, Antrobus R & Nathan JA (2015) The Proteasome Distinguishes between Heterotypic and Homotypic Lysine-11-Linked Polyubiquitin Chains. *Cell Reports*: 1–9

Haring JS, Badovinac VP & Harty JT (2006) Inflaming the CD8+ T Cell Response. *Immunity* 25: 19–29

Khan S, van den Broek M, Schwarz K, de Giuli R, Diener P a & Groettrup M (2001) Immunoproteasomes largely replace constitutive proteasomes during an antiviral and antibacterial immune response in the liver. *Journal of immunology (Baltimore, Md : 1950)* 167: 6859–68

Lv N, Zhao Y, Liu X, Ye L, Liang Z, Kang Y, Dong Y, Wang W, Kolliputi N & Shi L (2022) Dysfunctional telomeres through mitostress-induced cGAS/STING activation to aggravate immune senescence and viral pneumonia. *Aging Cell* 21

McArthur K, Whitehead LW, Heddleston JM, Li L, Padman BS, Oorschot V, Geoghegan ND, Chappaz S, Davidson S, San Chin H, *et al* (2018) BAK/BAX macropores facilitate mitochondrial herniation and mtDNA efflux during apoptosis. *Science* 359: eaao6047

McKinney EF, Lee JC, Jayne DRW, Lyons PA & Smith KGC (2015) T-cell exhaustion, co-stimulation and clinical outcome in autoimmunity and infection. *Nature* 523: 612–616

Meul T, Berschneider K, Schmitt S, Mayr CH, Mattner LF, Schiller HB, Yazgili AS, Wang X, Lukas C, Schlesser C, *et al* (2020) Mitochondrial Regulation of the 26S Proteasome. *Cell*

reports 32: 108059
Misko IS, Cross SM, Khanna R, Elliott SL, Schmidt C, Pye SJ & Silins SL (1999) Crossreactive recognition of viral, self, and bacterial peptide ligands by human class I-restricted cytotoxic T lymphocyte clonotypes: Implications for molecular mimicry in autoimmune disease. *Proc Natl Acad Sci USA* 96: 2279–2284
Münch C & Harper JW (2016) Mitochondrial unfolded protein response controls matrix pre-RNA processing and translation. *Nature* 534: 710–713
Oldstone MBA (1998) Molecular mimicry and immune-mediated diseases. *FASEB j* 12: 1255–1265
Schon E a, DiMauro S & Hirano M (2012) Human mitochondrial DNA: roles of inherited and somatic mutations. *Nature reviews Genetics* 13: 878–90
Studencka-Turski M, Çetin G, Junker H, Ebstein F & Krüger E (2019) Molecular Insight Into the IRE1α-Mediated Type I Interferon Response Induced by Proteasome Impairment in Myeloid Cells of the Brain. *Frontiers in Immunology* 10: 1–18
Sumida TS, Dulberg S, Schupp JC, Lincoln MR, Stillwell HA, Axisa P-P, Comi M, Unterman A, Kaminski N, Madi A, *et al* (2022) Type I interferon transcriptional network regulates expression of coinhibitory receptors in human T cells. *Nat Immunol* 23: 632–642
Trifunovic A, Wredenberg A, Falkenberg M, Spelbrink JN, Rovio AT, Bruder CECE, Bohlooly-Y M, Gldlöf S, Oldfors A, Wibom R, *et al* (2004) Premature ageing in mice expressing defective mitochondrial DNA polymerase. *Nature* 429: 417–423
Valdimarsson H, Thorleifsdottir RH, Sigurdardottir SL, Gudjonsson JE & Johnston A (2009) Psoriasis – as an autoimmune disease caused by molecular mimicry. *Trends in Immunology* 30: 494–501
West AP, Khoury-Hanold W, Staron M, Tal MC, Pineda CM, Lang SM, Bestwick M, Duguay BA, Raimundo N, MacDuff DA, *et al* (2015) Mitochondrial DNA stress primes the antiviral innate immune response. *Nature* 520: 553–557
Xian H, Watari K, Sanchez-Lopez E, Offenberger J, Onyuru J, Sampath H, Ying W, Hoffman HM, Shadel GS & Karin M (2022) Oxidized DNA fragments exit mitochondria via mPTP- and VDAC-dependent channels to activate NLRP3 inflammasome and interferon signaling. *Immunity* 55: 1370-1385.e8
Yu C-H, Davidson S, Harapas CR, Hilton JB, Mlodzianoski MJ, Laohamonthonkul P, Louis C, Low RRJ, Moecking J, De Nardo D, *et al* (2020) TDP-43 Triggers Mitochondrial DNA Release via mPTP to Activate cGAS/STING in ALS. *Cell* 183: 636-649.e18
Zhou L, Kong G, Palmisano I, Cencioni MT, Danzi M, De Virgiliis F, Chadwick JS, Crawford G, Yu Z, De Winter F, *et al* (2022) Reversible CD8 T cell–neuron cross-talk causes aging-dependent neuronal regenerative decline. *Science* 376: eabd5926

Dear Silke,

Thank you for submitting your revised manuscript to The EMBO Journal.

Your revision has now been seen by the original referees and their comments are provided below. As you can see from the comments, the referees appreciate the introduced changes and support publication here.

Referee #3 has a few remaining comments. Regarding the point " there is simply not more pIRF3 in the blot in Fig. 1D. Please remove this statement from the text" (I think the referee is referring to Fig. 2D). I have looked at the blot and I agree with the referee that it is not obvious that there is a difference in pIRF3. Please look at this again.

When you submit the revised manuscript will you also take care of the following points:

- Emails to the following bounced: Isis Fernandez, Kai Guo, Laylan Bramasole, Melanie Koenigshoff, Qianjiang Hu, Valeria Viteri

- The funding info for National Natural Science Foundation of China (82201448) is missing in the online submission system.

- You can only have 5 keywords

- COI needs to be renamed as "DISCLOSURE AND COMPETING INTERESTS STATEMENT"

- Please remove the Authors Contributions from the manuscript. The 'Author Contributions' section is replaced by the CRediT contributor roles taxonomy to specify the contributions of each author in the journal submission system. Please use the free text box in the 'author information' section of the manuscript submisssion system to provide more detailed descriptions (e.g., 'X provided intracellular Ca++ measurements in fig Y')

- Please complete the 1st page of the the author checklist

- Datasets should be renamed to Table EV1-EV3, as they are labeled in the legends

- Supplementary Table S1 and 2 should be re-labeled as Tables EV4 & 5

- I would also like to ask you to provide source data for the main figures. please upload one file/folder (zipped) per figure labelled Source Data figure 1 etc.

- Please upload a synopsis text => a summary statement plus 3-5 bullet points describing the key findings of the

- We also need a synopsis image => 550 wide by [200-400]

- Our publisher has also done their pre-publication check on your manuscript. When you log into the manuscript submission system you will see the file "Data Edited Manuscript file". Please take a look at the word file and the comments regarding the figure legends and respond to the issues.

- Each main and EV figure should be uploaded as one complete figure

- Please also fix the orders of the manuscript section

That should be all - if you have any questions just contact us.

Best Karin

Karin Dumstrei, PhD
Senior Editor
The EMBO Journal

Instructions for preparing your revised manuscript:

Use the link below to submit your revision:

https://emboj.msubmit.net/cgi-bin/main.plex

Referee #1:

The authors have comprehensively addressed all comments that I made.

Referee #2:

All my prior concerns were adequately addressed in the revised version of this manuscript and rebuttal.

Referee #3:

Mitochondrial stress and the activation of the cGAS/STING system are increasingly recognized as important events in pathophysiology. This study goes way beyond current knowledge in providing evidence that this pathway can regulate the immunoproteasome and may contribute to aberrant CD8-T cell activation and lung fibrosis. During revision, the authors have removed ambiguities, and they provide additional data, making the study convincing, solidly substantiating their conclusions. I have only a few minor points where the text has some inaccuracies that should be edited as follows:

I said this in the first round: there is simply not more pIRF3 in the blot in Fig. 1D. Please remove this statement from the text.

The text refers to silencing of cGAS or STING (Fig. 4C) but only cGAS is in the figure. Please correct in the text.

'Partially abrogated' (with reference to Fig. 5D) does not seem to make sense. 'Reduced' or similar would seem to describe the effect.

Response to reviewers EMBOJ-2022-110597R1

We would like to thank the referees for their careful revision and have addressed all remaining issues below.

Referee #3:

Mitochondrial stress and the activation of the cGAS/STING system are increasingly recognized as important events in pathophysiology. This study goes way beyond current knowledge in providing evidence that this pathway can regulate the immunoproteasome and may contribute to aberrant CD8-T cell activation and lung fibrosis. During revision, the authors have removed ambiguities, and they provide additional data, making the study convincing, solidly substantiating their conclusions. I have only a few minor points where the text has some inaccuracies that should be edited as follows:

1. I said this in the first round: there is simply not more pIRF3 in the blot in Fig. 1D. Please remove this statement from the text.
R1: We apologize for having overlooked this statement upon revision and have accordingly revised the statement. The paragraph now reads: *"….While we were unable to detect significant changes in the expression of cGAS and STING in the three distinct PolgA$^{mut/mut}$ cell lines compared to our three wildtype lines due to high clonal variation, levels were slightly higher in PolgA$^{mut/mut}$ cells. The same applied to the levels of phosphorylated - and thus activated - NFκB p65. Expression levels of IκBα were slightly lower in PolgA$^{mut/mut}$ cells (Figs. 2D and EV1D)…."*

2. The text refers to silencing of cGAS or STING (Fig. 4C) but only cGAS is in the figure. Please correct in the text.
R2: We thank the reviewer for pointing out this mistake and have accordingly corrected it.

3. 'Partially abrogated' (with reference to Fig. 5D) does not seem to make sense. 'Reduced' or similar would seem to describe the effect.
R3: We have changed the sentence to: *"Activation of CD8+ reporter T cells was reduced in PolgAmut/mut cells upon immunoproteasome inhibition (Fig. 5D)."*

2nd Revision - Editorial Decision 20th Feb 2023

Dear Silke,

Thank you for submitting the revised manuscript. I have now had a chance to look at the changes and all looks good.

I am therefore very pleased to accept the MS for publication here.

Congratulations on a nice study

Best Karin

Karin Dumstrei, PhD
Senior Editor
The EMBO Journal

Please note that it is EMBO Journal policy for the transcript of the editorial process (containing referee reports and your response letter) to be published as an online supplement to each paper. If you do NOT want this, you will need to inform the Editorial Office via email immediately. More information is available here:
https://www.embopress.org/page/journal/14602075/authorguide#transparentprocess

Your manuscript will be processed for publication in the journal by EMBO Press. Manuscripts in the PDF and electronic editions of The EMBO Journal will be copy edited, and you will be provided with page proofs prior to publication. Please note that supplementary information is not included in the proofs.

You will be contacted by Wiley Author Services to complete licensing and payment information. The required 'Page Charges Authorization Form' is available here: https://www.embopress.org/pb-assets/embo-site/tej_apc.pdf - please download and complete the form and return to embopressproduction@wiley.com

EMBO Press participates in many Publish and Read agreements that allow authors to publish Open Access with reduced/no publication charges. Check your eligibility: https://authorservices.wiley.com/author-resources/Journal-Authors/open-access/affiliation-policies-payments/index.html

Should you be planning a Press Release on your article, please get in contact with embojournal@wiley.com as early as possible, in order to coordinate publication and release dates.

If you have any questions, please do not hesitate to call or email the Editorial Office. Thank you for your contribution to The EMBO Journal.

** Click here to be directed to your login page: https://emboj.msubmit.net

EMBO Press Author Checklist

Corresponding Author Name: Silke Meiners	
Journal Submitted to: EMBO Journal	
EMBOJ-2022-110597R	

USEFUL LINKS FOR COMPLETING THIS FORM
The EMBO Journal - Author Guidelines
EMBO Reports - Author Guidelines
Molecular Systems Biology - Author Guidelines
EMBO Molecular Medicine - Author Guidelines

Reporting Checklist for Life Science Articles (updated January
This checklist is adapted from Materials Design Analysis Reporting (MDAR) Checklist for Authors. MDAR establishes a minimum set of requirements in transparent reporting in the life sciences (see Statement of Task: 10.31222/osf.io/9sm4x). Please follow the journal's guidelines in preparing your
Please note that a copy of this checklist will be published alongside your article.

Abridged guidelines for figures
1. Data
The data shown in figures should satisfy the following conditions:
- the data were obtained and processed according to the field's best practice and are presented to reflect the results of the experiments in an accurate and unbiased manner.
- ideally, figure panels should include only measurements that are directly comparable to each other and obtained with the same assay.
- plots include clearly labeled error bars for independent experiments and sample sizes. Unless justified, error bars should not be shown for technical
- if n<5, the individual data points from each experiment should be plotted. Any statistical test employed should be justified.
- Source Data should be included to report the data underlying figures according to the guidelines set out in the authorship guidelines on Data

2. Captions
Each figure caption should contain the following information, for each panel where they are relevant:
- a specification of the experimental system investigated (eg cell line, species name).
- the assay(s) and method(s) used to carry out the reported observations and measurements.
- an explicit mention of the biological and chemical entity(ies) that are being measured.
- an explicit mention of the biological and chemical entity(ies) that are altered/varied/perturbed in a controlled manner.
- the exact sample size (n) for each experimental group/condition, given as a number, not a range;
- a description of the sample collection allowing the reader to understand whether the samples represent technical or biological replicates (including how many animals, litters, cultures, etc.).
- a statement of how many times the experiment shown was independently replicated in the laboratory.
- definitions of statistical methods and measures:
  - common tests, such as t-test (please specify whether paired vs. unpaired), simple χ2 tests, Wilcoxon and Mann-Whitney tests, can be unambiguously identified by name only, but more complex techniques should be described in the methods section;
  - are tests one-sided or two-sided?
  - are there adjustments for multiple comparisons?
  - exact statistical test results, e.g., P values = x but not P values < x;
  - definition of 'center values' as median or average;
  - definition of error bars as s.d. or s.e.m.

Please complete ALL of the questions below.
Select "Not Applicable" only when the requested information is not relevant for your study.

Materials

Newly Created Materials	Information included in the manuscript?	In which section is the information available? (Reagents and Tools Table, Materials and Methods, Figures, Data Availability Section)
New materials and reagents need to be available; do any restrictions apply?	Not Applicable	Material and Methods

Antibodies	Information included in the manuscript?	In which section is the information available? (Reagents and Tools Table, Materials and Methods, Figures, Data Availability Section)
For **antibodies** provide the following information:<br>- Commercial antibodies: RRID (if possible) or supplier name, catalogue number and or/clone number<br>- Non-commercial: RRID or citation	Yes	Materials and Methods

DNA and RNA sequences	Information included in the manuscript?	In which section is the information available? (Reagents and Tools Table, Materials and Methods, Figures, Data Availability Section)
**Short novel DNA or RNA including primers, probes**: provide the sequences.	Yes	Table EV4 and Table EV5

Cell materials	Information included in the manuscript?	In which section is the information available? (Reagents and Tools Table, Materials and Methods, Figures, Data Availability Section)
**Cell lines:** Provide species information, strain. Provide accession number in repository **OR** supplier name, catalog number, clone number, and/**OR** RRID.	Yes	Materials and Methods
**Primary cultures:** Provide species, strain, sex of origin, genetic modification status.	Yes	Materials and Methods
Report if the cell lines were recently **authenticated** (e.g., by STR profiling) and tested for mycoplasma contamination.	Not Applicable	

Experimental animals	Information included in the manuscript?	In which section is the information available? (Reagents and Tools Table, Materials and Methods, Figures, Data Availability Section)
**Laboratory animals or Model organisms:** Provide species, strain, sex, age, genetic modification status. Provide accession number in repository **OR** supplier name, catalog number, clone number, **OR** RRID.	Yes	Materials and Methods
**Animal observed in or captured from the field:** Provide species, sex, and age where possible**.**	Not Applicable	
Please detail **housing and husbandry conditions**.	Not Applicable	

Plants and microbes	Information included in the manuscript?	In which section is the information available? (Reagents and Tools Table, Materials and Methods, Figures, Data Availability Section)
**Plants:** provide species and strain, ecotype and cultivar where relevant, unique accession number if available, and source (including location for collected wild specimens).	Not Applicable	
**Microbes:** provide species and strain, unique accession number if available, and source.	Not Applicable	

Human research participants	Information included in the manuscript?	In which section is the information available? (Reagents and Tools Table, Materials and Methods, Figures, Data Availability Section)
If collected and within the bounds of privacy constraints report on age, sex and gender or ethnicity for all study participants.	Not Applicable	

Core facilities	Information included in the manuscript?	In which section is the information available? (Reagents and Tools Table, Materials and Methods, Figures, Data Availability Section)
If your work benefited from core facilities, was their service mentioned in the acknowledgments section?	Not Applicable	

Design

Study protocol	Information included in the manuscript?	In which section is the information available? (Reagents and Tools Table, Materials and Methods, Figures, Data Availability Section)
If study protocol has been **pre-registered, provide DOI in the manuscript**. For clinical trials, provide the trial registration number **OR** cite DOI.	Not Applicable	
Report the **clinical trial registration number** (at ClinicalTrials.gov or equivalent), where applicable.	Not Applicable	

Laboratory protocol	Information included in the manuscript?	In which section is the information available? (Reagents and Tools Table, Materials and Methods, Figures, Data Availability Section)
Provide DOI OR other citation details if **external detailed step-by-step protocols** are available.	Not Applicable	

Experimental study design and statistics	Information included in the manuscript?	In which section is the information available? (Reagents and Tools Table, Materials and Methods, Figures, Data Availability Section)
Include a statement about **sample size** estimate even if no statistical methods were used.	Yes	Materials and Methods and Figure legends
Were any steps taken to minimize the effects of subjective bias when allocating animals/samples to treatment (e.g. **randomization procedure**)? If yes, have they been described?	Not Applicable	
Include a statement about **blinding** even if no blinding was done.	Not Applicable	
Describe **inclusion/exclusion criteria** if samples or animals were excluded from the analysis. Were the criteria pre-established?<br><br>If sample or data points were omitted from analysis, report if this was due to attrition or intentional exclusion and provide justification.	Not Applicable	
For every figure, are **statistical tests** justified as appropriate? Do the data meet the assumptions of the tests (e.g., normal distribution)? Describe any methods used to assess it. Is there an estimate of variation within each group of data? Is the variance similar between the groups that are being statistically compared?	Yes	Materials and methods and Figure Legends

Sample definition and in-laboratory replication	Information included in the manuscript?	In which section is the information available? (Reagents and Tools Table, Materials and Methods, Figures, Data Availability Section)
In the figure legends: state number of times the experiment was **replicated** in laboratory.	Yes	Figure legends
In the figure legends: define whether data describe **technical or biological replicates**.	Yes	figure legends

Ethics

Ethics	Information included in the manuscript?	In which section is the information available? (Reagents and Tools Table, Materials and Methods, Figures, Data Availability Section)
Studies involving **human participants**: State details of **authority granting ethics approval** (IRB or equivalent committee(s), provide reference number for approval.	Not Applicable	
Studies involving **human participants**: Include a statement confirming that **informed consent** was obtained from all subjects and that the experiments conformed to the principles set out in the WMA Declaration of Helsinki and the Department of Health and Human Services Belmont Report.	Not Applicable	
Studies involving **human participants:** For publication of **patient photos**, include a statement confirming that consent to publish was obtained.	Not Applicable	
Studies involving experimental **animals**: State details of **authority granting ethics approva**l (IRB or equivalent committee(s), provide reference number for approval. Include a statement of compliance with ethical regulations.	Not Applicable	
Studies involving **specimen and field samples:** State if relevant **permits** obtained, provide details of authority approving study; if none were required, explain why.	Not Applicable	

Dual Use Research of Concern (DURC)	Information included in the manuscript?	In which section is the information available? (Reagents and Tools Table, Materials and Methods, Figures, Data Availability Section)
Could your study fall under dual use research restrictions? Please check biosecurity documents and list of **select agents and toxins** (CDC): https://www.selectagents.gov/sat/list.htm	Not Applicable	
If you used a select agent, is the security level of the lab appropriate and reported in the manuscript?	Not Applicable	
If a study is subject to dual use research of concern regulations, is the name of the **authority granting approval and reference number** for the regulatory approval provided in the manuscript?	Not Applicable	

Reporting

The MDAR framework recommends adoption of discipline-specific guidelines, established and endorsed through community initiatives. Journals have their own policy about requiring specific guidelines and recommendations to complement MDAR.

Adherence to community standards	Information included in the manuscript?	In which section is the information available? (Reagents and Tools Table, Materials and Methods, Figures, Data Availability Section)
State if relevant guidelines or checklists (e.g., **ICMJE, MIBBI, ARRIVE, PRISMA**) have been followed or provided.	Not Applicable	
For **tumor marker prognostic studies,** we recommend that you follow the **REMARK** reporting guidelines (see link list at top right). See author guidelines, under 'Reporting Guidelines'. Please confirm you have followed these guidelines.	Not Applicable	
For **phase II and III randomized controlled trials**, please refer to the **CONSORT** flow diagram (see link list at top right) and submit the CONSORT checklist (see link list at top right) with your submission. See author guidelines, under 'Reporting Guidelines'. Please confirm you have submitted this list.	Not Applicable	

Data Availability

Data availability	Information included in the manuscript?	In which section is the information available? (Reagents and Tools Table, Materials and Methods, Figures, Data Availability Section)
Have **primary datasets** been deposited according to the journal's guidelines (see 'Data Deposition' section) and the respective accession numbers provided in the Data Availability Section?	Yes	proteomics data: ID=PXD019695
Were **human clinical and genomic datasets** deposited in a public access-controlled repository in accordance to ethical obligations to the patients and to the applicable consent agreement?	Not Applicable	
Are **computational models** that are central and integral to a study available without restrictions in a machine-readable form? Were the relevant accession numbers or links provided?	Not Applicable	
If publicly available data were reused, provide the respective **data citations in the reference list**.	Yes	
